# Gene clustering and copy number variation in alkaloid metabolic pathways of opium poppy

**DOI:** 10.1038/s41467-020-15040-2

**Published:** 2020-03-04

**Authors:** Qiushi Li, Sukanya Ramasamy, Pooja Singh, Jillian M. Hagel, Sonja M. Dunemann, Xue Chen, Rongji Chen, Lisa Yu, Joseph E. Tucker, Peter J. Facchini, Sam Yeaman

**Affiliations:** 1 Department of Biological Sciences, University of Calgary, Calgary, Alberta T2N 1N4 Canada; 2 Institute of Chinese Materia Medica, China Academy of Chinese Medical Sciences, Beijing, China; 3 Willow Biosciences Inc., 3655 36 Street N.W., Calgary, Alberta T2L 1Y8 Canada; 4 Department of Ecosystem and Public Health, University of Calgary, Calgary, Alberta T2N 1N4 Canada; 5 Department of Biochemistry and Molecular Biology, University of Calgary, Calgary, Alberta T2N 1N4 Canada

**Keywords:** Genome evolution, Gene expression profiling, Structural variation, Secondary metabolism

## Abstract

Genes in plant secondary metabolic pathways enable biosynthesis of a range of medically and industrially important compounds, and are often clustered on chromosomes. Here, we study genomic clustering in the benzylisoquinoline alkaloid (BIA) pathway in opium poppy (*Papaver somniferum*), exploring relationships between gene expression, copy number variation, and metabolite production. We use Hi-C to improve the existing draft genome assembly, yielding chromosome-scale scaffolds that include 35 previously unanchored BIA genes. We find that co-expression of BIA genes increases within clusters and identify candidates with unknown function based on clustering and covariation in expression and alkaloid production. Copy number variation in critical BIA genes correlates with stark differences in alkaloid production, linking noscapine production with an 11-gene deletion, and increased thebaine/decreased morphine production with deletion of a T6ODM cluster. Our results show that the opium poppy genome is still dynamically evolving in ways that contribute to medically and industrially important phenotypes.

## Abstract (web summary)

The opium poppy has been a source of painkilling drugs synthesized by the benzylisoquinoline alkaloid pathway. Here, the authors report an improved genome assembly and reveal gene clustering and copy number variation in alkaloid metabolic pathways.

## Introduction

**Fig. 1:**
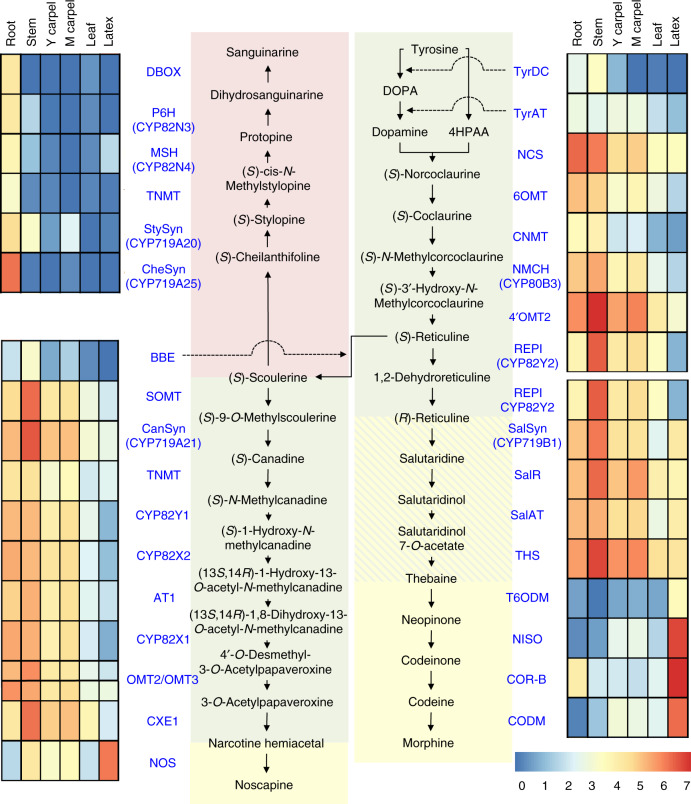
Genes involved in BIA biosynthesis in six tissues of the opium poppy. Cellular localization of gene-expression activity (log_10_ scale) in sieve elements (olive-green), laticifers (yellow), both sieve elements and laticifers (yellow with diagonal olive-green lines), and roots (red) is shown. Gene names are in blue text (aliases are in brackets) and their alkaloid products are in black text (final products are in bold). YC young capsule, MC mature capsule. Source data are provided as a Source Data file.

The opium poppy has been a double-edged sword for human civilization: the pharmaceutical products of its alkaloid biosynthetic pathways provide incalculable benefits for pain relief, addiction treatment^1^, and potentially cancer therapy^2^, but have simultaneously driven historic and current epidemics in opioid addiction and overdose deaths^3^. As a product of artificial selection and natural variation within the species, different plant lineages show striking variation in alkaloid content^4^. For example, some opium poppy cultivars produce high levels of morphine and codeine, whereas others instead accumulate copious amounts of thebaine and oripavine^5^. Decades-long effort has established the basic pathways leading to opiates and other benzylisoquinoline (BIA) alkaloids (Fig. 1; Supplementary Fig. 1). However, very little is known regarding the genetic and biochemical regulation of BIA biosynthesis, and basic elements of pathway functionality remain elusive. For example, while the morphine pathway operates across at least two cell types^6^, mechanisms of intermediate shuttling between cells are unexplored and transport proteins need to be identified. Further, how opium poppy chaperones and stores such abundant quantities of cytotoxic and highly hydrophobic alkaloids in an aqueous cellular environment is unknown. Understanding how the architecture and regulation of the genome contributes to phenotypic variation is critical to answering these fundamental questions. Genome-guided elucidation of plant-based mechanisms is essential for reproducing these metabolic pathways in alternative systems using synthetic biology methods, and provides fascinating insights into plant evolution.

Clustering of genes in metabolic pathways has been found in the genome of opium poppy^7,8^, and is a common feature in plant genomes^9^, including the terpene pathway in tomato^10^, phytocassane in rice^11^, and DIMBOA in maize^12,13^, but it is unclear why such clustering evolves. Genes situated in close proximity on a chromosome tend to share similar patterns of expression^14,15^, and may reside within the same topological associated domain^16^, so such architectures may have evolved to optimize and coordinate gene expression. Also, if there is variation in ecological niche among populations, clustering of loci can evolve to reduce the rate at which recombination breaks up coadapted complexes of alleles^17^. Given the clustering found in many different plant genomes, and particularly for the secondary metabolic pathways, it is important to understand how such pathways are organized and regulated.

A recently published assembly and analysis of the opium poppy genome revealed that a whole genome duplication event occurred about 7.8 million years ago in the poppy lineage, which may have facilitated some of the rearrangements and genome evolution that occurred in this species^8^. This assembly (hereafter Guo assembly) revealed clustering of five genes involved in alkaloid production adjacent to the previously described noscapine cluster^7^, and examined various hypotheses for their evolution. This analysis also revealed several paralogous and co-localized copies of T6ODM and CODM, which are both involved in the production of codeine and morphine, and used a gene co-expression analysis to identify a number of other putative gene clusters that are likely unrelated to alkaloid production. However, many genes that are critically important for BIA metabolism were not included on chromosome-scale scaffolds in this preliminary assembly, including NISO, THS, and NCS^18,19^, limiting our ability to comprehensively assess the extent of clustering of these genes.

Here, we use Hi–C to improve the existing assembly, and explore patterns of clustering, gene expression, and copy number variation (CNV) in the BIA pathway genes. In particular, we focus our attention on the PR10 and MLP genes, which are highly expressed in opium poppy latex^6,19^, but have not been comprehensively studied at a functional scale. To explore how BIA genes are regulated and parse the effect of clustering and pathway membership, we use a combination of RNAseq and assays of alkaloid concentration across developmental timepoints and plant tissues. Finally, we resequence the genomes of nine other opium poppy accessions with a range of alkaloid production profiles and examine patterns of BIA gene CNV and potential effects on alkaloid production. Taken together, these analyses show that the genes involved with alkaloid synthesis in opium poppy are dynamically evolving, and identify a large number of clustered genes with unknown function that are closely related to pathway genes of known function. These genes of unknown function represent viable candidates for evaluation as enzymes, transporters, chaperones, regulatory proteins, and other components required for in planta pathway functionality. Moreover, positive identification of functional parts continues to furnish synthetic biology projects with additional tools to effectively assemble biosynthetic pathways in alternative systems such as microbes. Genome-aided discovery has proven to be an effective strategy toward increasing product yields, particularly for opiate production in yeast fermentation systems^18,20^.

## Results

### Building an improved assembly by Hi–C rescaffolding

Our effort to improve the genome assembly of *Papaver somniferum* was based on re-scaffolding the recently published high-quality draft assembly^8^ using a de novo Hi–C dataset (see section “Methods”). After Hi–C scaffolding, contigs clustered into 11 chromosome-scale scaffolds (hereafter, cScafs, Supplementary Fig. 2). The genome size of our final assembly remains basically the same as that of the Guo assembly (2.71 Gb), but the number of sequences mapped onto cScafs is substantially increased: 820 input contigs were clustered into cScafs, accounting for 97.7% of the total length of the Guo assembly (Supplementary Table 1). Our assembly placed 122 contigs/scaffolds (~420.9 Mb) onto cScafs that were previously unanchored in the Guo assembly, representing a 15.4% increase in chromosome-scale scaffolded genome size (Supplementary Fig. 3). There is broad agreement between the two assemblies in terms of the synteny of contigs, with only 2.8% of contigs placed into different chromosome-scale scaffolds in the two assemblies (Supplementary Fig. 4). Interestingly, in some cases an off-diagonal signature in the Hi–C contact map is apparent among chromosomes that Guo et al. previously identified as having segments derived from the same ancestral chromosome, due to the whole genome duplication (Supplementary Fig. 5).

To further assess assembly quality, we compared the proportion of successfully mapping mate-pair reads, which increased from 75% of reads in the Guo assembly to 85% in our assembly, with fewer reads having their mate mapped to a different chromosome (Supplementary Table 2). The broad agreement between assemblies constructed with very different technologies (Hi–C vs. linkage map) suggests there are likely few errors in the coarse assembly. By contrast, there are many places where the two assemblies differ in terms of the ordering and orientation of contigs within the cScafs (Supplementary Fig. 4), suggesting further work is required to refine the assembly at this finer scale. Such differences could arise because of true differences in the genome architecture between the accessions sequenced, or because of bioinformatic errors in one or both drafts. We further evaluated the accuracy of these assemblies by mapping ~10× PacBio reads from two libraries: the HN1 cultivar used by Guo et al. and the PS7 cultivar used in our Hi–C assembly (Supplementary Method 1). We then counted the number of scaffolded-gaps with >1 read mapping across the gap (mapping quality >10). Reads from both cultivars showed similar patterns, with ~25% of scaffold joins in our Hi–C assembly having mapped reads, compared to ~4.7% of scaffold joins in the Guo et al. assembly (Supplementary Table 3). Taken together, these analyses show that the Hi-C scaffolding has improved both assembly quality and contiguity, but that it remains a draft quality genome.

The BIA pathways of interest included 42 well-described biosynthetic genes (Supplementary Fig. 1), as well as 16 genes with unknown function from the PR10 gene family that encode the abundant major latex proteins (MLPs) found in the alkaloid-rich opium poppy latex (Supplementary Data 1; Supplementary Fig. 1). We used a combination of gmap (v2017-06-20)^21^ and BLAST^22^ (v2.5.0+) to map these genes to our new assembly, and identified a total of 109 mappings, with all 58 core BIA genes and 51 of their close paralogs placed on cScafs (Supplementary Data 2). The 51 paralogs include 12 likely pseudogenes, but we retain these in subsequent analysis of clustering, as they may have previously contributed to BIA synthesis and are therefore of interest from an evolutionary perspective. By comparison, using the same approach only 46 of the core BIA genes and 24 of their close paralogs were placed on chromosome-scale scaffolds in the Guo assembly, while the remaining 39 were found on smaller unscaffolded contigs.

### Chromosomal clustering of benzylisoquinoline pathway genes

To assess spatial organization of the BIA pathway genes on chromosomes, we used a simple heuristic to identify clusters as any cases where neighboring BIA genes were situated <*X* bp away from each other (regardless of the distribution of non-BIA genes), and examined the effect of varying this *X* cutoff on the amount of clustering. While this approach is simpler than algorithms such as plantiSMASH^23^, which incorporates information from gene-expression data to identify clusters, it allows us to focus directly on the chromosomal arrangement of genes to identify clusters. We can then explore the effect of clustering on patterns of gene expression, which would otherwise be confounded by the cluster designations if expression data was used to delineate them.

**Fig. 2:**
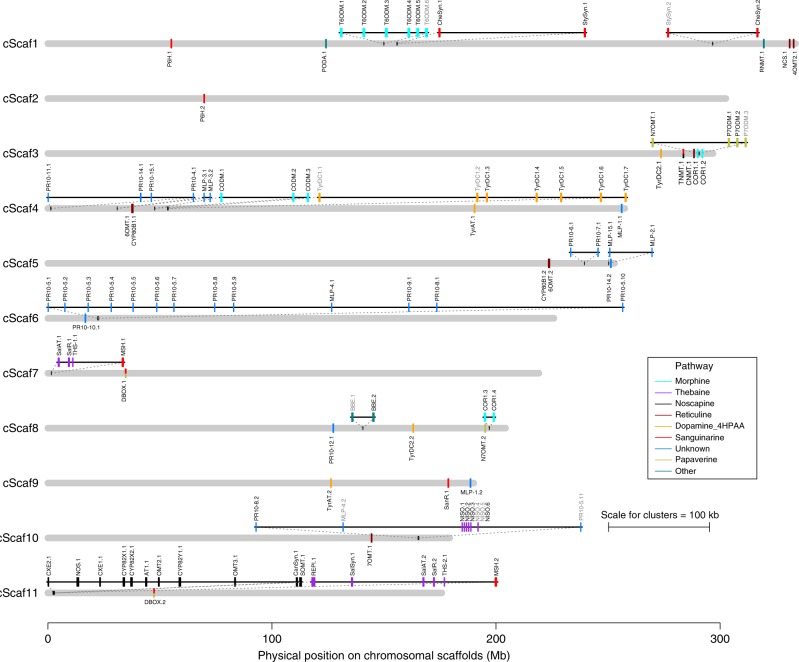
Genomic organization of the BIA pathway genes and their paralogs. Genes and paralogs are colored according to the portion of the pathway in which they reside, with putative pseudogenes indicated by grey text. Note that TNMT and DBOX are shared between two pathways, as indicated by their joint coloring. Relationship between cScafs and *P. somniferum* chromosomes as defined by Guo et al. is shown in Supplementary Table 12. Source data are provided as a Source Data file.

**Fig. 3:**
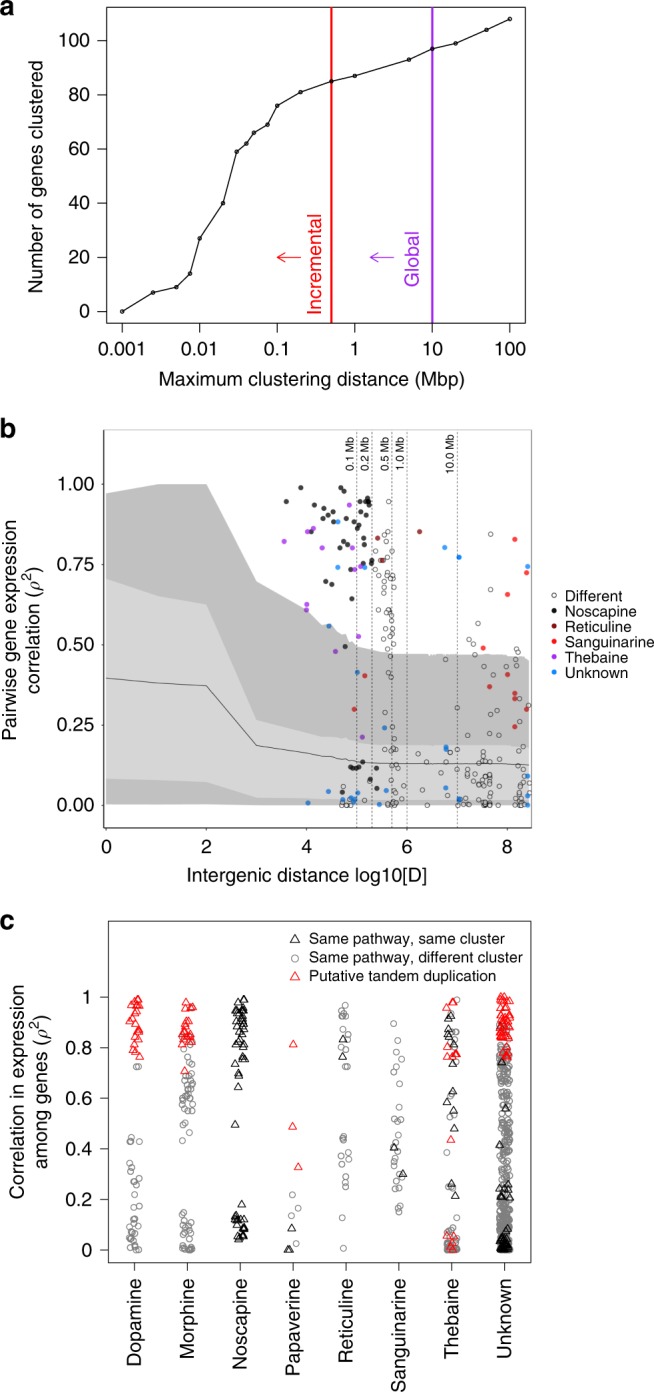
Clustering and gene co-expression of BIA pathway genes. The incremental and global tests find significant clustering of BIA genes (**a**). Gene coexpression decays with distance among genes, but is higher for BIA pathway genes (**b**) and higher for genes that are tandemly duplicated or in the same pathway (**c**). Panel (**b**) shows that pairwise gene coexpression between non-BIA pathway genes decreases more rapidly than for BIA genes in the same pathway and even BIA genes in different pathways. In (**b**) the dark gray regions represent the 75th–95th and 5th–25th percentiles, the light gray regions represent the 25th–75th percentiles, and the black solid line represents the 50th percentile. Source data are provided as a Source Data file.

Chromosomal clustering of pathway genes is striking: 70% of the 109 BIA genes and paralogs are situated in clusters separated by at most 100 kb (Fig. 2), often in large arrays with few interspersed non-pathway genes. Using a more permissive definition for clustering, as many as 89% of all BIA genes are contained within clusters when the maximum nearest-neighbor distance is 10 Mb, with a total distance of clusters spanning only 2.3% of the 11 cScafs (Fig. 3a; Supplementary Table 4). The greatest increase in the number of clustered genes occurs with cutoffs between 10 and 100 kb (Fig. 3a; Supplementary Table 4), showing that most of the BIA genes are clustered within relatively small regions of the genome. The observed clustering is statistically significant up to nearest-neighbor distances of 500 kb under the conservative incremental test, and up to distances of 10 Mb under the less conservative global test (Fig. 3a; Supplementary Table 4; see section “Methods”). In both cases, this clustering remains significant when potential tandem duplicates are excluded (Supplementary Table 5). Unless otherwise specified, we use the <500 kb clustering distance for all subsequent analyses.

Some clusters appear to have arisen through extensive tandem duplication, such as the groups of multiple copies of T6ODM, NISO, and PR10-5, where each copy also has associated duplications of flanking TEs (Supplementary Fig. 6; see below). Other PR10 and MLP genes also appear to have evolved by tandem duplication (Fig. 2), and similar patterns have been previously reported in grape, where most PR10 genes are found on a single chromosome with many tandem duplicates^24^. In addition to T6ODM, the other genes in the morphine pathway (CODM and COR1) also occur in tightly clustered arrays with one or more of their paralogs, as does TyrDC1 (Fig. 2). In other cases, however, there are clusters that contain genes from several families, which precludes tandem duplication as the primary means of cluster evolution. The previously identified noscapine and thebaine clusters^7,8^ are clear examples of tightly linked arrays of genes from different families, but we also find more loosely linked clusters across greater chromosomal distances that are composed of genes from several different families. For example, our assembly reveals that cScaf1 harbors two genes from different families in the reticuline pathway (NCS and 4’OMT2) separated by <2 Mb (which were on unplaced contigs in the Guo assembly), while cScaf3 has a large loosely linked cluster spanning <8.5 Mb with 8 genes from three different families, including TNMT, CNMT, COR1, N7OMT, and three copies of P7ODM (7 of which span ~3.7 Mb). Our assembly also places a previously unmapped large tandem array of seven copies of TyrDC1 within 6 Mb of a tandem array of CODM on cScaf4, and the previously unmapped cluster of the baine paralogs (SalR, SalAT, and THS) onto cScaf7 (Supplementary Fig. 3).

Interestingly, when likely tandem duplicates are excluded the proportion of genes residing in clusters identified at a scale of <500 kb differs significantly among sub-pathways (*χ*^2^ test, d*f* = 1, simulated *p* value = 0.0005), with 11/13 of the genes in the noscapine subpathway residing in clusters, 8/9 genes in thebaine, 6/13 genes in sanguinarine, 0/4 genes in morphine, and 0/5 genes in dopamine 4HPAA (Supplementary Table 6). Reticuline has 0/8 genes clustered at <100 kb, 4/8 genes clustered at <500 kb, and 7/8 clustered at <5 Mb, showing it is more loosely clustered than the thebaine and noscapine pathway genes. Genes in the morphine pathway do not appear in clusters with other BIA genes until distances of 5–10 Mb (Supplementary Table 6), and at this scale, they are co-localized with a number of BIA genes from other pathways but do not tend to co-localize together (Fig. 2; Supplementary Table 7). Perhaps most provocatively, for the 22 pathway genes with unknown function, many of which are the latex-expressed PR10/MLP genes, 73% reside in clusters at distances of <500 kb, providing a tantalizing target in the search for factors that increase the biosynthetic production of medicinally important alkaloids. Indeed, the recent characterization of the gene encoding thebaine synthase^18^ was guided in part by localization within a cluster of known biosynthetic genes. Taken together, our assembly reveals that significant nonrandom spatial distribution of BIA genes occurs across broad spatial scales on opium poppy chromosomes. Whereas Guo et al.^8^ did not identify clustering for many of the important BIA genes (i.e., COR, TNMT, CODM, and T6ODM), our less restrictive definition of clustering identifies some interesting patterns of co-localization across distances of 5–10 Mb, which often yield groupings across multiple pathways.

### Patterns of expression in BIA pathway genes

To investigate variation in gene expression during ontogeny and in different tissues of the opium poppy, we sequenced transcriptome data from seven developmental timepoints and six tissue types (*n* = 13). Several interesting patterns emerged regarding the biosynthesis and accumulation of BIAs, with clear upregulation of sanguinarine genes exclusively in the roots and upregulation of the final steps of morphine and noscapine pathways in the latex (Fig. 1, Supplementary Figs. 7, 8). For the purposes of this study, stem tissue was cut and washed free of laticifer content prior to analysis, to permit examination of gene expression (or metabolite content) in non-latex vasculature^6^. This approach, together with knowledge of previous cell-specific localization studies^25^ made it possible to map early segments of the opiate pathway to sieve elements or other non-latex vasculature and later steps to laticifers. For example, we find that genes leading up to and including REPI in the morphine pathway are highly expressed in stem but not latex, while genes including, and downstream of T6ODM are primarily expressed in the latex (Fig. 1). Our results suggest that transport of alkaloid intermediates between R-reticuline and thebaine from sieve elements to laticifers is sporadic and likely dependent on alkaloid availability. Gene expression patterns (Fig. 1) and the abundance of thebaine in latex (Supplementary Fig. 9) suggest that thebaine could be an important transport point for the pathway, although cell-to-cell shuttling has yet to be established using more direct means in planta.

The tight co-expression of several genes of unknown function (MLP2, MLP15, PR10-4, and PR10-5) with key morphine and thebaine genes expressed in the latex advocates for their importance in BIA biosynthesis (Supplementary Figs. 7, 8). It is commonly observed that co-expression is elevated above background levels for genes situated close together on chromosomes, likely due to some combination of the effects of chromatin neighborhood and sharing of *cis*-regulatory machinery^14,26^. In non-BIA genes in opium poppy, we observe elevated co-expression among neighboring genes at distances of <0.1 Mb, with the closest genes exhibiting mean *ρ*^2^ = 0.37, and average co-expression levels decaying to the background genomic level of co-expression of *ρ*^2^ = 0.13 beyond 0.1 Mb (Fig. 3b). However, this effect of spatial proximity is very mild, explaining only *r*^2^ < 0.032 of the variation in co-expression at distances of <0.1 Mb (with lower *r*^2^ at greater distances; see section “Methods”).

We find that BIA genes situated within the same cluster tend to have high levels of co-expression, often far above the expectation given the distance between them (Fig. 3b). This is not particularly surprising, as genes in the same pathway are expected to be co-regulated for physiological reasons, and genes in the same pathway but different clusters often also had high levels of co-expression (Fig. 3b; Supplementary Figs. 8, 10). Averaging across pathways and clusters and excluding comparisons between putative tandem duplicates, mean rates of co-expression were: *ρ*^2^ = 0.42 for BIA genes in the same pathway and cluster, *ρ*^2^ = 0.32 for BIA genes in the same pathway but different clusters, and *ρ*^2^ = 0.20 for BIA genes in different clusters and pathways. Expanding our analysis to the non-BIA genes present in the BIA clusters, levels of co-expression were low, with *ρ*^2^ = 0.24 across all comparisons.

If natural selection for gene co-regulation drives cluster evolution^27^, we would expect high co-expression among all BIA genes within a cluster. The thebaine pathway provides an example consistent with this: its genes are found in three separate clusters and co-expression is high within each cluster, but not necessarily high among clusters, with the tandemly duplicated NISO genes showing different patterns of expression than the other two clusters (Supplementary Fig. 11a). However, there are other examples that deviate from this expectation: while most genes in the noscapine cluster are highly co-expressed, the expression patterns of CXE2 and NOS are very different and uncorrelated with each other (Supplementary Fig. 11b). More strikingly, a cluster of seven genes from different pathways situated within 4 Mb of each on cScaf3 shows hardly any co-expression (Supplementary Fig. 11c). Thus, while co-expression is commonly elevated within BIA gene clusters, especially when they are very close together on a chromosome, this is not a universal pattern and low co-expression is also commonly observed within clusters (Fig. 3c).

BIA genes that are putative tandem duplicates had particularly high levels of co-expression, with *ρ*^2^ = 0.83, although there were some notable exceptions in the thebaine and noscapine clusters (Fig. 3c). By contrast, other non-BIA tandem duplicates (from anywhere in the genome, separated by <500 kb) tended to have lower levels of co-expression (*ρ*^2^ = 0.66), and non-BIA gene duplicates located more distantly (>500 kb) had even lower levels of co-expression (*ρ*^2^ = 0.46; Supplementary Fig. 12). This heightened conservation of expression pattern among BIA tandem duplicates suggests their evolution may have been driven to boost the overall production of mRNA and downstream proteins in these pathways, as there is little evidence for the changes in expression expected with sub- or neo-functionalization^28^. Alternatively, the BIA-gene duplications may have arisen more recently and had less time to diverge in their patterns of gene expression. Similar expansion of gene copy number has been found in the cannabinoid pathways in *Cannabis sativa*, where it is also thought to aid increased alkaloid production^29^.

### Covariation between expression and metabolite production

**Fig. 4:**
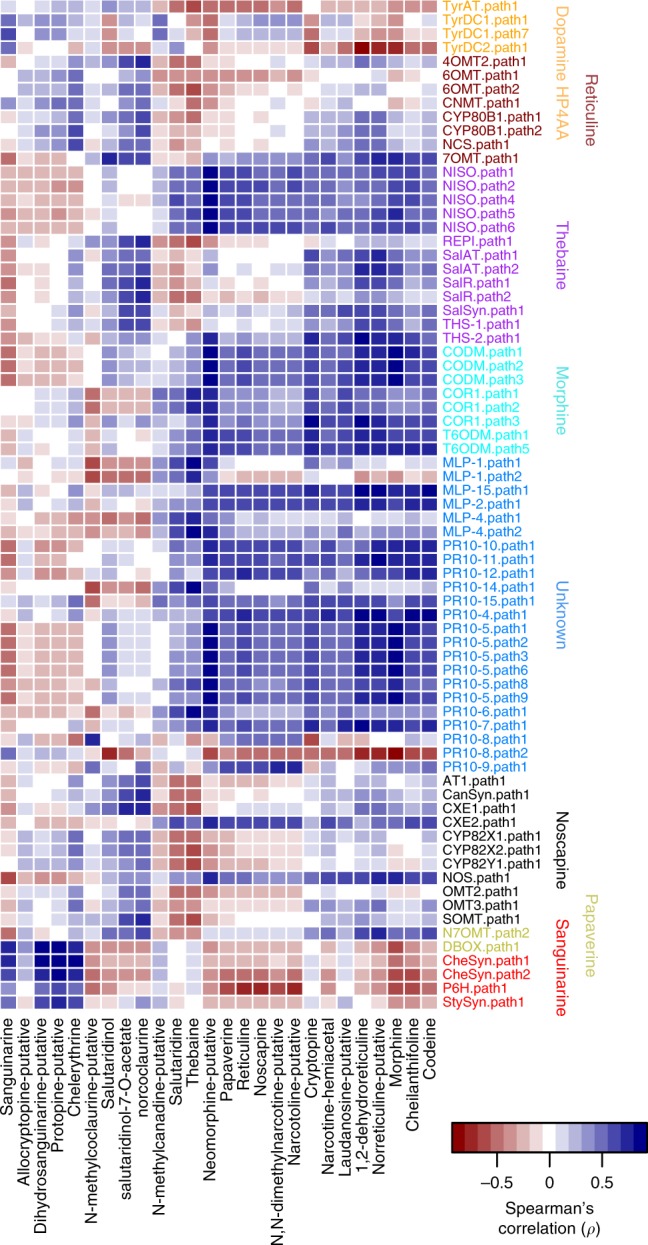
Covariation between gene expression and alkaloid concentration across tissues and timepoints. Relationships are shown for the main alkaloids of interest and all pathway genes with detectable expression (>0.1 TPM) in at least five libraries. Colors for pathway genes correspond to the coloring in Fig. 2. Source data are provided as a Source Data file.

To explore how patterns of expression in BIA genes covary with alkaloid concentrations, we used a mass spectrometer to assess the amount of each compound in the same tissues and timepoints used for gene expression analysis. As expected^30^, concentrations of morphine, codeine, noscapine increased dramatically in latex, and thebaine production was maximized in early seedling stages while concentration of other compounds increased in concentration at later stages (Supplementary Figs. 13, 14). Interestingly, gene expression and alkaloid concentration covary across tissues and timepoints in broadly similar ways for most pathways of interest (Fig. 4). Morphine alkaloids in particular are very strongly positively covarying with the genes in the morphine and thebaine pathways that lead to their production, but also with a large number of genes with unknown function (PR10 and MLP). Of special note are the PR10–14 and MLP-1 genes, which covary very strongly with thebaine production, and the PR10-5, MLP15, and MLP2 genes, which covary strongly with the end products of the morphine pathway. These genes therefore represent tantalizing candidates for future study that may prove to have important roles driving flux through these pathways. Covariation between pathway genes and pathway end products is also very strong for the sanguinarine pathway, but much less consistent for genes in the noscapine and papaverine pathways (Fig. 4). Given the history of artificial selection for increased production of morphine alkaloids, these patterns likely vary considerably among varieties, which can vary considerably in their production of alkaloids (see below).

### CNV among cultivars

**Fig. 5:**
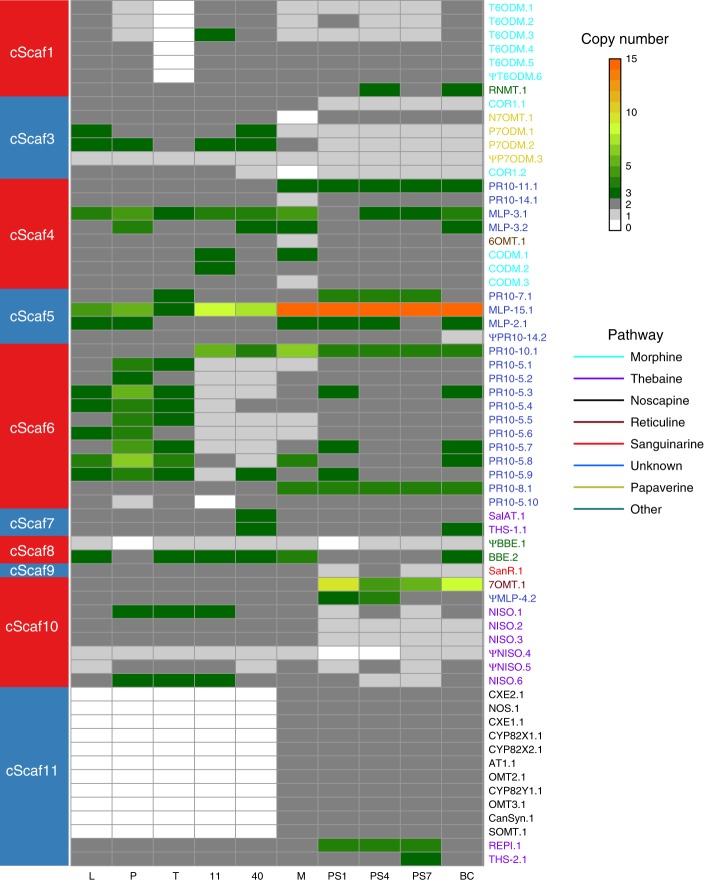
Copy number variation in the BIA pathway genes. CNVs were identified across ten opium poppy cultivars, and are colored according to pathway and ordered by cScaf. Copy number of two corresponds to diploidy, which is the state of the draft assembly, while 0 is a complete deletion. Colors for pathway genes correspond to the coloring in Fig. 2. Relationship between cScafs and *P. somniferum* chromosomes as defined by Guo et al. is shown in Supplementary Table 12. Source data are provided as a Source Data file.

To examine the genetic variation among different agriculturally bred cultivars of opium poppy, we sequenced the genomes of 10 cultivars at depths ranging from 16.8× to 26.1× (Supplementary Table 8), and identified regions harboring CNV. Our analyses revealed that the opium poppy genome is dynamically evolving, with CNV detected in 63 out of the 109 BIA genes and their paralogs (Fig. 5). Broadly, pathway genes within clusters were about three times more likely to exhibit CNV than those outside of clusters (clusters identified at a scale of <1 Mb, 66.7% vs. 22.7%; *χ*^2^ test, d*f* = 1, *p* value = 0.000193). The high CNV trend persists when excluding the genes from the noscapine cluster (61.8% vs. 22.7%; *χ*^2^ test, d*f* = 1, *p* value = 0.001206) and the significance is insensitive to the scale of clustering distance (Supplementary Table 9).

**Fig. 6:**
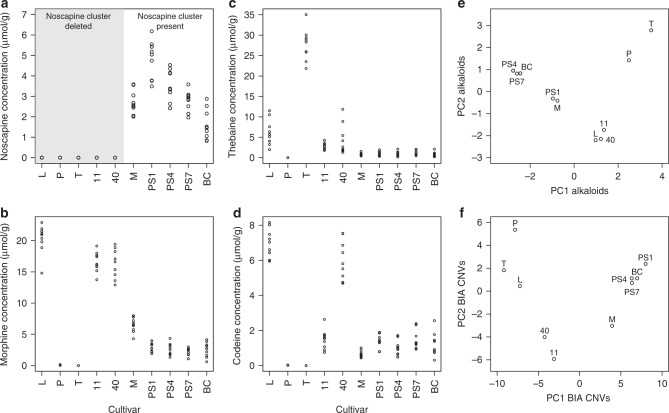
Alkaloid production and copy number variation of pathway genes in opium poppy. Alkaloid content measured in stem tissues of nine replicate plants from each of the ten sequenced cultivars for noscapine (**a**), morphine (**b**), thebaine (**c**), and codeine (**d**). Principal Components Analysis reveals strong similarities between cultivars in their profiles of alkaloid production (**e**) and copy number variation (**f**). Source data are provided as a Source Data file.

Cultivars with similar CNV profiles in their BIA genes (Fig. 6f) also tended to have similar profiles in their alkaloid production (Fig. 6e; Mantel test on distance matrix: *r* = 0.75, *p* value = 0.002). This similarity was not observed between the CNV profiles for non-BIA genes (Supplementary Fig. 15) and either the alkaloids (Mantel test, *r* = −0.090, *p* value = 0.662) or BIA gene CNVs (*r* = −0.074, *p* value = 0.657), illustrating that these similarities do not arise simply from patterns of relatedness among cultivars. At the individual gene level, strong covariation was observed between CNVs and the alkaloids produced by the genes involved. Most prominently, the entire noscapine pathway containing 11 biosynthetic genes is deleted in five of the 10 cultivars (L, P, T, 11 and 40; Fig. 5), which is linked to lack of noscapine production in these cultivars (Fig. 6a). MLP-15 is massively duplicated across all cultivars, with fifteen copies observed in cultivars M, PS1, PS4, PS7, and BC (Fig. 5), which is reflected in its extreme expression profile in the latex (Supplementary Figs. 7, 8). As these varieties also produce high levels of noscapine (Fig. 6a), this gene may play an important role in noscapine production, which is consistent with the positive relation observed between MLP-15 expression and noscapine production (Fig. 4). Similarly, the entire cluster of tandemly duplicated copies of T6ODM is deleted from the T cultivar (Fig. 5), which is likely responsible for the lack of any production of morphine and codeine, despite very high concentrations of thebaine (Fig. 6b–d; Supplementary Fig. 1). Taken together, these patterns of covariation between CNVs in BIA genes and alkaloid production suggest that the same mechanisms that originally lead to the evolution of this highly clustered architecture are still operating in the opium poppy genome.

### Transposable element content

Transposable elements (TEs) typically make up a significant part of plant genomes and are often involved in rearrangements, either through direct movement of DNA during transposition or ectopic recombination^31,32^. Like many plant genomes, a large fraction of the opium poppy genome (68.3%) is composed of TEs, made up largely of LTR and unknown elements, but with small proportions of DNA, Heiltron, and LINE/SINE elements (Supplementary Fig. 16). In 5 of the 18 BIA gene clusters there was significant enrichment for at least one class of TE, with the most common being DNA/hAt and LINE/L1 transposons (Supplementary Table 10), but also including helitrons, which are sometimes involved in TE-mediated gene movement^33^. Several TE types show evidence of a recent burst of activity characterized by a peak of many insertions with little sequence divergence (Supplementary Fig. 17), which was observed for helitron, DNA/hAT-Tag1, DNA/MuLE-MuDR, and LINE/L1-Tx1 elements. However, in most cases, the mean age of TEs within clusters was either older or nonsignificantly different from the overall background age in the genome (Supplementary Table 11).

TEs may have played an important role in the evolution of tandem duplications, as several genes present in multiple closely linked copies also have flanking TEs that are similarly duplicated. This is seen with: hAT-TAG1 and L1 fragments flanking copies of T6ODM; DNA/MULE-MuDr, LTR/Copia, and DNA/PIF-Harbinger flanking copies of PR10-5, LTR/Copia flanking copies of NISO and LINE/L1, RC/Helitron flanking copies of TyrDC1 (Supplementary Fig. 6). The formation of these direct duplications may have been mediated by TEs through ectopic recombination or other mechanisms such as reversed-ends transposition in the case of hAT-Tag^34^. Overall, TE-mediated gene movement does not seem to provide a general explanation for the evolution of these clusters, given the heterogeneity among clusters in their TE composition and similarity in age-class distribution. However, as TEs are common throughout the genome, they may have facilitated cluster evolution through ectopic recombination, particularly where tandem duplications have occurred, and the lack of statistical enrichment of TEs in some clusters does not mean they were evolutionarily unimportant.

## Discussion

Clustering of genes in the BIA metabolic pathways of opium poppy is heterogeneous: the thebaine and noscapine pathways are highly clustered, whereas the morphine pathway shows no clustering beyond its extensive tandem duplications, and the sanguinarine pathway has only minimal clustering (Fig. 2). Our results show that the genomic architecture of these pathways is still evolving, likely through some combination of natural and artificial selection. A deletion of the entire noscapine pathway is found in five cultivars lacking noscapine, while a deletion of the tandemly duplicated cluster of T6ODM genes (in the morphine pathway) is found in a cultivar lacking morphine and codeine but with strongly increased thebaine production (Figs. 5, 6). We also find dramatic increases in copy number for the MLP15 and 7OMT genes in some cultivars, and more moderate variation in PR10’s and other pathway genes (Fig. 5). Given that tandem duplicates in the BIA pathway genes tend to have highly conserved co-expression patterns (as opposed to sub- or neo-functionalization), it seems likely that duplications provide the means for increasing flux through these important pathways.

Despite extensive evidence for clustering, the reasons for its evolution remain obscure: gene co-expression is elevated within clusters of BIA pathway genes (Fig. 3c) but co-expression is also elevated among nonclustered pathway genes as well (Fig. 3c) and there are prominent exceptions where clustered genes show uncorrelated patterns of expression (Supplementary Fig. 11c). Outside of the BIA genes, co-expression is only elevated at very small spatial scales (<100 kb; Fig. 3b), suggesting that this mechanism may apply to some of the most tightly linked clusters, but is unlikely to facilitate the evolution of clusters at broader scales. An alternative explanation is that clusters evolve to reduce the rate of recombination between genes involved in local adaptation^17^. If there are trade-offs between plant growth and the production of defensive BIA compounds, spatial variation in herbivory could drive local adaptation^35^, with BIA production favored in areas of high herbivory and disfavored elsewhere. This mechanism could apply across large chromosomal scales, as the map distance (in cm) over which clustering is favored is proportional to the strength of selection (*s*) on the locally adapted alleles^17,36^. It could potentially explain the evolution of more loosely linked clusters, such as the grouping of eight genes from multiple pathways on cScaf3 that spans 8.5 Mb (~2.9% of the chromosome), as this would require comparatively weak selection coefficients. However, given that the benefits of clustering for recombination suppression occur over relatively large distances^17^, it seems unlikely that this would yield a cluster as tightly linked as the noscapine cluster. Future study of variation among landraces of opium poppy inhabiting different environments could yield insights into the potential importance of local adaptation in driving genome evolution.

Regardless of the reasons for the evolution of gene clustering, it has now been found so commonly in plant secondary metabolism^9^ that it provides a useful starting point in the discovery of novel, unforeseen genes contributing to these pathways of medical and industrial importance. In some cases, genome-guided approaches have identified essential parts within pathways that were already considered completely elucidated at the biochemical level. For example, while the discovery of REPI in 2015 was heralded as the final required catalytic component of opiate biosynthesis^37–39^, gene cluster evidence in poppy allowed the subsequent discovery of both THS^18^ and a purine uptake permease-like gene encoding an alkaloid-specific transporter^20^. Inclusion of THS in yeast engineered to produce opiates from simple precursors boosted thebaine yields by >20-fold^18^, whereas inclusion of permease-like transporters elevated thebaine yields from salutaridine feed by nearly 80-fold in engineered strains^20^. Examples of how gene clustering facilitates discovery of components essential for synthetic biology extends past opiates to noscapine alkaloids. First reporting of a noscapine cluster in opium poppy^7^ pinpointed the involvement of several catalysts, whose roles were later established^40^ enabling noscapine production by fermentation in engineered *Saccharomyces cerevisiae*^41^. Beyond physical co-clustering, co-expression, evolutionary co-occurrence, and epigenetic co-regulation have all been used to identify pathway components in a suite of plant pathways, enabling functional reconstitution of natural product biosynthesis in alternative production systems^42^.

Our findings provide an important preliminary characterization of the likely massive pool of variation in functionally important genes in agricultural and landrace cultivars of opium poppy. Understanding how variation in gene expression, copy number, and protein sequence contribute to pharmaceutical alkaloid biosynthesis in opium poppy is critical for the development of synthetic biology and other industrial applications to produce existing and novel drugs, and eventually to reduce the cost and increase accessibility of key drugs to combat the current opioid epidemic. Improved assembly and analysis of the opium poppy genome provides an important step towards this goal, and suggests that many genes of unknown function may reside within clusters of known alkaloid biosynthetic genes.

## Methods

### Plant cultivation

Opium poppy (*Papaver somniferum*) plants were cultivated in climate-controlled chambers under a 16 h photoperiod provided by a combination of Cool White fluorescent and incandescent lighting, and at a temperature of 20 °C/18 °C (light/dark). The opium poppy cultivars used were high-thebaine (T), Przemko (P), Marianne (M), Louisiana (L), and Bea’s Choice (BC)^4,43,44^. Additional cultivars were gifts provided by Prairie Plant Systems Inc. (now CanniMed Therapeutics Inc., owned by Aurora Cannabis Inc., www.auroramj.com). All research involving controlled materials was performed with appropriate government approvals.

### Selection of seedling and mature plant organs

Dry seeds of *P. somniferum* (cultivar PS7) were germinated under sterile conditions on wet filter paper (Whatman no. 1). A sufficient number of seedlings representing seven different stages of development were collected for (1) alkaloid metabolite analysis; and (2) RNA extraction for the purpose of establishing transcriptome libraries. Seeds imbibed with water for 24 h represented Stage 1; seedlings with visible radicals (after ~4 days) represented Stage 2; seedlings with visible cotyledons and root (~7 days) represented Stage 3; seedlings exhibiting first pair of leaves (~10 days) represented Stage 4; seedlings with 2 pairs of leaves (~14 days) represented Stage 5; seedlings with 3 pairs of leaves (~21 days) represented Stage 6; and seedlings left to mature 7 days beyond Stage 6 represented Stage 7 (Supplementary Fig. 9). In addition to seedlings, organs of mature opium poppy plants (cultivar PS7) were harvested as follows. Plants were cultivated in soil under standard greenhouse conditions for ~3 months to maturity, i.e., anthesis, at which time organs were harvested. Both tap root and branch roots were washed and collected. For stem, 5 cm lengths extending from young capsules were collected and sliced into ~1 mm sections and incubated in water for 1 h to remove latex^6^. Young and mature capsules were harvested immediately at anthesis, and five days following, respectively. Latex, which represents the cytoplasm of specialized laticifer cells, was collected as exudate emerging from lanced young capsules. Genomic and metabolite analyses of nine additional *P. somniferum* cultivars, along with cultivar PS7, were carried out using mature plants and Stage 7 seedlings, respectively. Tissue selection and extractions were performed using the same protocol as for cultivar PS7.

### Genomic DNA and total RNA extraction

One gram of frozen opium poppy stems from cultivar PS7 was ground to a fine powder and extracted using 6 mL of prewarmed (65 °C) cetyltrimethyl ammonium bromide (CTAB) buffer (2% wt/vol CTAB, 100 mM Tris-HCl, pH 8.0, 2 M NaCl, 25 mM EDTA, pH 8.0, 1% wt/vol) PVP, 2% (vol/vol) 2-mercaptoethanol). Incubation was performed at 65 °C for 30 min and vortexing was done occasionally followed by two extractions with an equal volume of chloroform/isoamyl alcohol (24:1, vol/vol). Genomic DNA was precipitated from the upper aqueous phase in a clean tube and equal volume of −20 °C isopropanol was added. The mixture was incubated at 4 °C for 30 min, and then centrifuged at 13,000*g* for 20 min at 4 °C. The DNA pellet was dissolved in 2 mL of TE buffer (100 mM Tris-HCl, pH 8.0, 25 mM EDTA) and treated with 5 μL of RNase A (10 mg/mL) at 37 °C for 20 min, and the mixture was extracted again using an equal volume of chloroform/isoamyl alcohol (24:1, vol/vol). The addition of (i) sodium acetate, pH 5.3, at a final concentration of 0.3 M and (ii) an equal volume of cold (−20 °C) isopropanol resulted in the precipitation of genomic DNA in the extracted upper aqueous phase. The mixture was incubated at 4 °C for 20 min and then centrifuged at 13,000*g* for 20 min at 4 °C. Three rinses of the DNA pellet were done with 70% (vol/vol) ethanol, which was then dried in a vacuum desiccator and finally dissolved in TE buffer. Total RNA was extracted using CTAB^45^ from finely ground plant tissues using a TissueLyser (Qiagen).

### Shotgun genome and transcriptome sequencing

For the cultivar PS7, we prepared one Illumina Truseq Paired-End (PE) library using the ~420 bp size-selected genomic DNA and one Illumina Nextera Mate-Pair (MP) library using the 7–10 kb jumps of genomic DNA for the purposes of identifying CNVs and assessing the potency of different anchoring approaches. These two libraries were sequenced on the Illumina HiSeq 2000 as 2 × 100 bp reads. For the other nine cultivars (L, P, T, 11, 40, M, PS1, PS4 and BC), polymerase chain reaction (PCR)-free Illumina Truseq PE libraries were separately prepared with 480–620 bp insert-sizes and were sequenced on the Illumina HiSeq X as 2 × 150 bp reads. Stranded libraries were constructed with the Illumina Truseq Kit for each of the mRNA samples representing seven seedling stages and tissues from stem, root, leaf, mature capsule, and young capsule. These 12 libraries were sent for 2 × 125 bp sequencing on the Illumina HiSeq 2500 platform. To further investigate the gene expression profile in the latex sample, three stranded libraries were prepared following the same protocol and were sequenced as 2 × 100 bp reads on the Illumina HiSeq 2000 platform. All the shotgun library preparation and sequencing were performed at the McGill University and Genome Quebec Innovation Centre.

### Estimation of the genome size and sequencing depth

We used the Kmergenie^46^ (v 1.7048) to screen the appropriate K-mer to establish the kmer_freq_hash table for each of the 10 cultivars. The hybrid mode of GCE (v.1.0.0)^47^ was used in estimating the genome size following a Bayes model based method. The depth of resequencing was then calculated with the total base amount and the estimated genome size of each cultivar.

### Hi–C rescaffolding and positioning of the BIA genes

Chromatin conformation capture data of PS7 was generated using a Phase Genomics Proximo Hi-C Plant Kit. Following the manufacturer’s instructions, a formaldehyde solution was used to treat intact leaf cells and cause crosslinking, followed by digestion using the Sau3AI restriction enzyme, and proximity ligation with biotinylated nucleotides. The resulting chimeric molecules are not necessarily close to each other in the true genome, but represent regions of chromosome that were close together in the nucleus. Finally, molecules were pulled down with streptavidin beads and used to prepare libraries for Illumina sequencing. Sequencing was performed on an Illumina NextSeq 500. A total of 236,766,981 PE80 read pairs were then aligned to the split sequences of the Guo assembly using BWA-MEM^48^ (v0.7.12) with the -5SP and -t 8 options specified, and all other options default. SAMBLASTER^49^ (v0.1.24) was used to flag PCR duplicates, which were later excluded from analysis. Alignments were then filtered with samtools^50^ (v1.5, with htslib 1.5) using the -F 2304 filtering flag to remove non-primary and secondary alignments.

Phase Genomics’ Proximo Hi–C genome scaffolding platform was used to create cScafs from the input assembly as described in Bickhart et al.^51^. The sequences of the Guo assembly were broken into segments at gaps of 100 Ns, representing the inter-scaffold gaps of unknown size, and at large gaps of ≥1000 Ns. The resulting split genome was comprised of 35,732 resulting segments, which were considered as contigs in the subsequent processing. The lengths of these contigs varied from 132 bp to 38.3 Mb, with the N50 length and the N50 number of the contigs of 7.6 Mb and 104, which provides a draft assembly with sufficient contiguity for making high confidence Hi-C based proximity-guided rescaffolding. As in the LACHESIS method^52^ (v2017), this process calculates how commonly different areas of the genome were ligated together during the Hi–C process, normalized by the density of Sau3AI restriction sites on each contig. It then constructs scaffolds that optimize the expected contact frequency patterns. We performed approximately 150,000 separate runs of the Proximo protocol to optimize the scaffold construction and concordance with Hi–C data.The automatic scaffolding result was manually reviewed in the Juicebox Assembly Tool (v1.9.1)^53,54^ described in the 3D de novo assembly pipeline^55^ for visual identification and interactive correction of the remaining errors in the Hi–C contact map. Final genome assembly was generated with an open source script juicebox_assemly_converter.py of juicebox_scripts [https://github.com/phasegenomics/juicebox_scripts]. See Supplementary Method 2 for further details. Relationship between cScafs and *P. somniferum* chromosomes as defined by Guo et al. is shown in Supplementary Table 12.

We positioned the 58 benzylisoquinoline alkaloid (BIA) pathway genes and their close paralogs in our improved genome assembly and the Guo assembly by referring to their previously characterized transcripts and protein sequences. Similarly, the cDNA mapping tool gmap^21^ was used to align the CDS sequences to the genomes to generate gene structures. The best hits of the gmap result were selected to obtain the full-length gene for the blastn^22^ (evalue 1e−10) against the genome to identify duplicates more sensitively. The ≥90% identity and ≥90% coverage filtering were applied to both the gmap and blastn results, which were subsequently merged into a list of 109 entries by bedtools^56^ (v2.27.1) intersection module. To identify the putative pseudogenes, the amino acid sequences of the 58 genes were used to predict the gene model of the 109 entries by GTH^57^ (v1.7.0). All the 109 genes were manually checked using BioEdit^58^ (v7.1) for coding-sequence decay, such as frameshifts and premature stop codons that result in the occurring of pseudogenes.

### Testing enrichment of pathway genes within clusters

We used a simple algorithm to identify clusters as any arrays where at least two BIA pathway genes resided within *X* bp of each other, regardless of whether other non-BIA genes were interspersed, and examined the effect of a range of choices for *X* on the amount of clustering. We use two approaches to assess significance of the observed clustering: a less conservative global test that considers clustering independently at a range of spatial scales, and a more conservative incremental test, where tests are conducted across successively larger spatial scales, and the contribution of genes to clustering at smaller scales is disregarded for each successively larger scale.

For the global test: for all 109 BIA genes, the number of genes per scaffold was retained and a new start position was drawn at random for each gene. The clustering algorithm was run on this permuted set and clusters were identified as before, and the number of genes occurring in clusters was recorded. Empirical *p* values were calculated as the number of replicates where the number of genes occurring in clusters in the permuted set was greater than the observed number of genes in clusters for the given value of *X*. To test whether clustering was also enriched independently of tandem duplications, we curated a reduced set of pathway genes that included only nontandemly duplicated genes. We then reran cluster identification algorithm and the same modified permutation test on this reduced set.

For the incremental test: for all 109 BIA genes, we begin by identifying all genes that are clustered at a small nearest-neighbor distance (1 kb). We then permute the locations of the remaining nonclustered genes within each cScaf and calculate how many would be clustered given the next largest nearest-neighbor distance (5 kb). We run this permutation 10,000 times and calculate the *p* value as the proportion of replicates where the number of clustered genes is greater than the observed number of clustered genes at 5 kb. This process is then repeated for each successively greater nearest-neighbor distance (1 kb, 5 kb, 10 kb, 50 kb, 100 kb, 500 kb, 1 Mb, 5 Mb, 10 Mb, 50 Mb, and 100 Mb), each time ignoring the clustering of genes that were clustered at the previous distance and testing only the remaining non-clustered genes. This method is more conservative than the global method because each successive test of clustering across broader distances does not leverage clustering at the previous distances, but its power depends critically on the spacing of the intervals.

### Testing enrichment of TEs in clusters

A TE library was created following the basic Maker protocol for repeat library construction [http://weatherby.genetics.utah.edu/MAKER/wiki/index.php/Repeat_Library_-Construction-Basic] based on the Guo assembly. TEs were identified by RepeatMasker^59^ (v.4.0.7), and mapped to the draft genome by Maker-P^60^ (v2.3.10). To examine whether each cluster showed enrichment of any given class of TE, we counted all classes of TE present within the cluster, and then for each class, randomly sampled a window of the genome of the same size as the cluster and counted the number of TE occurrences within the window. We repeated this random window sampling 10,000 times for each class in each focal cluster, and generated the empirical *p* value as the proportion of replicates when the number of TEs exceeded the number observed in the focal cluster. We then performed a Bonferroni adjustment on the cutoff for statistical significance within each cluster, dividing 0.05 by the number of tests in the cluster.

We used the RepeatMasker packages calcDivergenceFromAlign.pl and createRepeatLandscape.pl to estimate Kimura distances and to create repeat landscapes for the whole genome and for enriched TE classes. We used SEDEF^61^ (v.1.1-23) to identify large tandem duplications in clusters. Some of the resulting duplication annotations were fragmented as a result of masked interspersed repeats. We numbered duplicated fragments based on their order along the chromosome. Fragments that mapped to each other received the same number. We combined and extended fragments manually to reconstruct complete tandem repeats.

### Identification of CNVs

To understand the extent of BIA pathway genes’ CNVs, we employed the Read-depth (RD) method for protein-coding genes CNV genotyping across the 10 *P. somniferum* cultivars. Firstly, the raw shotgun genome sequencing data for each cultivar was processed with fastp^62^ (v0.19.5) to get the high-quality FASTQ files with -q 20 -l 51 options specified. The trimmed and filtered reads were aligned to the improved Poppy draft genome using BWA-MEM^48^ with default parameters, then were sorted with samtools^50^ (v1.3.1). PCR duplicates and Illumina HiSeq X specific duplicates were marked by the Picard’s Mark Duplicates module. CNVs were identified with the reads mapping density according to the result of statistical analysis performed by the CNVnator^63^ (v0.3.2), which separated the whole genome into nonoverlapping 1000 bp blocks and utilizes the count of mapped reads within each block as the RD signal.

### Transcriptome profiling

To determine the transcriptome profile of the BIA pathway genes, we used Illumina RNAseq data from individual libraries constructed from six tissue types (root, stem, leaf, young carpal, mature carpal, and latex) and seven developmental stages (S1–S7). As these RNAseq libraries represent a wide range of developmental timepoints and tissues, and have high depth of coverage (average of 59.2 Mb reads per library), they should have sufficient power to identify genes with detectable expression except for cases that are very specialized to particular timepoints or tissues. Raw RNA-seq reads were checked for quality with FastQC^64^ (v 0.11.7). Illumina sequencing adapters were trimmed and low quality reads (Phred33 < 30) were discarded using Trimmomatic^65^ (v0.38). The trimmed and filtered paired reads were mapped against our assembled Poppy genome using Hisat2^66^ (v2.1.0) with default parameters with the addition of the–dta parameter, which optimizes output for transcript assemblers. Mapped reads were sorted using samtools^50^ (v1.3.1) and only uniquely mapped reads were retained to avoid paralog-related multimapping issues. Coverage of genes annotated in the draft genome were quantified using the Stringtie^66^ (v1.3.5) and raw gene counts were normalized to TPM (transcript counts per million) using a perl script [https://raw.githubusercontent.com/griffithlab/rnaseq_tutorial/master/scripts/-stringtie_expression_matrix.pl]. No novel transcripts were annotated. Further details are provided in Supplementary Method 3.

### Analysis of gene co-expression patterns

To investigate the genome-wide relationship between gene co-expression and distance and contrast it with BIA pathway genes we calculated the pairwise distance (*D*) and expression correlation (Spearman’s rho) for linked genes on the 11 main scaffolds using an in house R script (genedecay.R). *D* was defined as the distance between the transcription start sites of two genes. Putative tandem duplicates of BIA pathway genes were removed from this analysis as they would generate spurious strong negative correlations between expression and distance because duplicates are located in close proximity and may have shared gene expression patterns due to shared ancestry. Spearman’s ρ was plotted as a function of the binned intergenic distance, which was log_10_ transformed for plotting. In addition, the extent of co-expression amongst unlinked BIA pathway genes was analyzed and compared to the background level of co-expression between unlinked genes. To make this analysis computationally feasible, the background was calculated by randomly sampling 100 unique genes from each of the 11 scaffolds. To assess patterns of gene co-expression within and among pathways, and within and among clusters, we used the <500 kb cluster designation and the pathways as indicated in Fig. 2, excluding genes that contribute to more than one pathway (DBOX, TNMT, and BBE). To assess patterns of co-expression among putatively duplicated non-BIA genes (both in tandem and genome-wide), we used the mapping of the genes identified by Guo et al.^8^ described above to identify cases where the same gene mapped to multiple locations and considered each of these mappings as a putative duplicate. We then calculated co-expression between genes as above using Spearman’s ρ on the log10 transformation of expression counts for all genes with >0.1 TPM in at least 5 of the 14 libraries.

### Metabolite analysis

To ensure sufficient tissue was extracted, particularly for early stages of development when seedlings were very small, up to 15 individuals were separated into 1 of 3 replicate sample pools, with each replicate pool consisting of ~100 mg fresh weight in pre-weighed 1.5-mL tubes. For root, stem, leaf, capsule, and latex of mature plants, sufficient material allowed the use of one individual per replicate, totaling 6 biological replicates (nine replicates of the stem tissues were used for the comparison of alkaloid content across 10 cultivars). Each replicate was extracted with 1200 mL acetonitrile (ACN) and sedimented (10,000*g*) to remove insoluble matter. Insoluble matter was extracted twice more with 500 mL ACN, sedimented, and supernatants were removed and pooled in a fresh tube. Insoluble matter was retained for the purposes of obtaining the dry weight of each sample, enabling normalization of alkaloid quantity per dry mass. Specifically, the insoluble fraction was heated at 60 °C under vacuum for 3 days for complete desiccation and weighed. Subtracting the predetermined Eppendorf tube weight from the tube and dry pellet weight yielded the tissue dry weight. To ensure a narrow dynamic range of metabolites for facile MS-based quantification, this dry weight was also used to quantitatively adjust the supernatant concentrations between replicates, such that an equivalent amount (in terms of dry matter) was injected for MS analysis for all samples. Following this adjustment, 500 µl from each ACN extract was diluted 1:100 with ACN, and a subsequent 500 mL was diluted 1:1000 with ACN. Undiluted (1:1) and diluted (1:100 and 1:1000) fractions were all analyzed by high-resolution LC-ESI-LTQ-Orbitrap-XL mass spectrometry to ensure quantification of target alkaloids was performed within linear range concentrations, as indicated by standard curves. High-resolution LC-ESI-LTQ-Orbitrap-XL mass spectrometry (MS) was performed following the method described by Morris et al.^67^, with liquid chromatography carried out using an UltiMate 3000 HPLC (Thermo Fisher Scientific) equipped with a Poroshell 120 SB-C18 column (Agilent Technologies) in place of an Accela HPLC system (Thermo Fisher Scientific) equipped with a Zorbax C18 column (Agilent Technologies). Details are as follows: fractionation was performed on five microliters of undiluted (1:1) or diluted (1:100 or 1:1000) at a flow rate of 0.5 mL min^−1^ and a gradient of Solvent A (10 mM ammonium acetate, pH 5.5, 5% ACN) and Solvent B (100% ACN). Fractionation used 100–80% Solvent A over 5 min, 80–50% over 3 min, 50–0% over 3 min, isocratic at 0% for 2 min, 0–100% over 0.1 min, and isocratic at 100% for 1.9 min. The total run time was 15 min but data were collected for only 10 min. Positive-ion mode was used to run Heated ESI source and interface conditions with the following conditions: vaporizer temperature, 400 °C; source voltage, 3 kV; sheath gas, 60 au, auxiliary gas, 20 au; capillary temperature, 380 °C; capillary voltage, 6 V; tube lens, 45 V. Three scan events were performed in data-dependent LTQ-Orbitrap-XL (Thermo Scientific) instrumentation using parallel detection mode. High-resolution FTMS from 150 to 450 *m*/*z* with ion injection time of 500 ms and scan time of approximately 1.5 s was used for the first scan. The second and third scans (approximately 0.5 s each) collected CID spectra in the ion trap, where the parent ions represented the first- and second-most abundant alkaloid masses, respectively, as determined by fast Fourier transform preview using a parent ion mass list corresponding to exact masses of known alkaloids. Dynamic-exclusion and reject-ion-mass-list features were enabled. External and internal calibration procedures ensured <2 ppm error. The Quan Browser feature of Thermo X-Calibur (v3.1) was employed for automated peak identification and quantification. Raw files (.raw) were quantified in batches according to the manual *Xcalibur: Getting Productive* (Thermo Fisher Scientific). Package XCMS was run with R to use the following functions and parameters: centWave [ppm = 2.0, peakwidth = c(10,60), prefilter = c(35,000), sleep = 0], retcor and obiwarp [plottype = c(“deviation”], group [bw = 5, minfrac = 0.5, mzwid = 0.015], fillPeaks. Mass lists were visualized by writing data to tables in Microsoft Excel. Resulting mass lists were manually inspected for exact *m/z* and retention times matching those of authentic standards. Matches were confirmed by inspection of corresponding CID data for authentic standards collected during LTQ analysis. Standard curves for target alkaloids at various concentrations were processed alongside experimental samples, enabling quantification.

### Statistics and reproducibility

In all cases, the above tests were only conducted once and the data that is reported above constitutes the only attempt made in each case, with the exception of experiments used to quantify metabolites for Supplementary Fig. 9, which were conducted twice with similar outcomes (only one result was reported here).

### Reporting summary

Further information on research design is available in the Nature Research Reporting Summary linked to this article.

## Supplementary information

Supplementary Information

Peer Review

Reporting Summary

Description of Additional Supplementary Files

Supplementary Data 1

Supplementary Data 2

## Data Availability

A reporting summary for this article is available as a Supplementary Information file. Data supporting the findings of this work are available within the paper and its Supplementary Information files. The datasets generated and analyzed during the current study are available from the corresponding author upon request. Raw sequence data, including the Hi-C-seq, PacBio validation, RNA-seq and the re-sequencing for 10 cultivars are available in the NCBI Sequence Read Archive under BioProject PRJNA508405 [https://www.ncbi.nlm.nih.gov/bioproject/PRJNA508405]. A total of 11 cScafs are available in the NCBI Genbank with WGS accession numbers of CP048211-CP04821 [https://www.ncbi.nlm.nih.gov/nuccore?term = CP048211:CP048221%5BACCN%5D]. Other data, including alkaloid content, RNA-seq read counts and Hi-C contact matrices, which are necessary to generate all of the figures, tables, and datapoints are available on Dryad archive (10.5061/dryad.m0cfxpnz9). The source data underlying Figs. 1–6, Supplementary Figs. 3, 4, and 7–17, as well as Supplementary Table 3 are provided as a Source Data file.

## Source data

Source Data file

## Code availability

All scripts to generate the figures and tables in the manuscript are included in the Dryad archive (10.5061/dryad.m0cfxpnz9).
