# Author Correction: Gene clustering and copy number variation in alkaloid metabolic pathways of opium poppy

**DOI:** 10.1038/s41467-020-16467-3

**Published:** 2020-06-04

**Authors:** Qiushi Li, Sukanya Ramasamy, Pooja Singh, Jillian M. Hagel, Sonja M. Dunemann, Xue Chen, Rongji Chen, Lisa Yu, Joseph E. Tucker, Peter J. Facchini, Sam Yeaman

**Affiliations:** 1 Department of Biological Sciences, University of Calgary, Calgary, Alberta T2N 1N4 Canada; 2 Institute of Chinese Materia Medica, China Academy of Chinese Medical Sciences, Beijing, China; 3 Willow Biosciences Inc., 3655 36 Street N.W., Calgary, Alberta T2L 1Y8 Canada; 4 Department of Ecosystem and Public Health, University of Calgary, Calgary, Alberta T2N 1N4 Canada; 5 Department of Biochemistry and Molecular Biology, University of Calgary, Calgary, Alberta T2N 1N4 Canada

**Keywords:** Genome evolution, Gene expression profiling, Structural variation, Secondary metabolism

Correction to: *Nature Communications* 10.1038/s41467-020-15040-2, published online 04 March 2020.

In the original version of this Article, chromosome numbering was based on the size of the assembled scaffolds. This chromosome-numbering system is different from the existing linkage map-based assignment (Guo et al., *Science* 362, 343–347 (2018)). To maintain the established convention and avoid confusion, the following changes have been made to the original Article and its Supplementary Information:

1. A new Supplementary Table 12 has been added to the Supplementary Information file, to clarify the relationship between cScaf number and Guo et al. chromosome number. Supplementary Table 12 is cited in the main text Methods section “Hi-C re-scaffolding and positioning of the BIA genes”.

2. The statement “Relationship between cScafs and *P. somniferum* chromosomes as defined by Guo et al. is shown in Supplementary Table 12” has been added to the legends of Figs. 2 and 5, Supplementary Figs. 3, 4, 5 and 11, Supplementary Tables 10 and 11 as well as Supplementary Data 2.

3. In the Methods section, the sentence “Relationship between cScafs and *P. somniferum* chromosomes as defined by Guo et al. is shown in Supplementary Table 12” was added to the sentence “See Supplementary Method 2 for further details”.

4. In Supplementary Fig. 4, *x*- and *y*-axes of the 11 displays have been relabelled as “Guo Chromosome #” and “Hi-C cScaf #”, respectively, according to the relationship between cScafs and *P. somniferum* chromosomes defined by Guo et al.

5. In Supplementary Fig. 5, all five labels of “Hi-C Chr #” inside the display have been changed to “Hi-C cScaf #”; in addition, the *x*-axis label of “Hi-C chromosome numbering” has been changed to “Hi-C cScaf numbering”.

In addition, in the original version of this Article, colour coding for cellular localizations of the two biosynthetic pathways in Fig. 1 was omitted from the display, but specified in the legend.

The above points have been corrected in both the PDF and HTML versions of the Article. Corrected Supplementary Information files, Description of Additional Supplementary Files and Supplementary Data 2 have been added to the HTML version of the Article.

