# Supplementary material for: Gene clustering and copy number variation in alkaloid metabolic pathways of opium poppy
Source: Nat Commun. 2020 Mar 4;11:1190. doi: 10.1038/s41467-020-15040-2 (PMC7055283; doi:10.1038/s41467-020-15040-2)
Supplement: Supplementary file 1 — Supplementary Information [file 41467_2020_15040_MOESM1_ESM.pdf]

Gene clustering and copy number variation in alkaloid metabolic pathways of opium poppy

Li *et al.*

Supplementary Method 1. PacBio raw reads mapping

PacBio raw reads (SRR7668135 - SRR7668143) of HN1 (SAMN08604995) were downloaded from the NCBI-SRA. For PS7 in this study, PacBio RSII sequencing of long insert-size libraries was carried out on 58 SMRT cells. After filtering out the reads shorter than 1kb, 31.6 Gb PacBio reads for HN1 (read-length up to 98.3 kb, reads N50 of 12.1 kb) and 30.1 Gb PacBio reads for PS7 (read-length up to 67.1 kb, reads N50 of 10.7 kb) were used in the mapping to the joins using minimap2[1], with present option "map-pb" optimized for the long noisy reads alignment.

Supplementary Method 2. Coordinate transformation of protein-coding genes

The protein-coding genes annotation of the Poppy genome reported by Guo *et al.*[2] was transformed to the Hi-C improved genome assembly following the GEAN[3] pipeline of "Lift over reference genome annotation to a *de novo* assembly genome using CDS sequence mapping" in the manual. Splice-aware model of minimap2[1] was used for aligning the primary transcript CDS sequence to the new genome assembly.

Supplementary Method 3. Gene expression

PCA analysis of normalised gene expression counts from 100,709 genes from all the 13 samples grouped the two early developmental stages, S1 and S2, together (Supplementary Fig. 17). The mid to late developmental stages (S3-S7) grouped together with PC1 separating the stages according to increasing developmental maturity. The mature capsule and the latex samples exhibit very similar gene expression patterns, as expected, while the root is distinct from all the other samples. Similarities in gene expression of the 109 annotated BIA pathway genes from the 13 above-mentioned RNAseq samples was studied using unsupervised hierarchical clustering of the gene expression counts (Supplementary Fig. 6). The genes were divided by expression

patterns into two major groups with several sub-groups: (1) (a) genes involved in reticuline, noscapine, and thebaine synthesis which were expressed at moderate to high levels (3 < log10[exp] < 6) across most samples and (b) genes involved in the morphine and unknown (MLP/PR10) pathways that were highly expressed in the latex sample (log10[exp] > 6); (2) (a) genes involved in sanguinarine expressed almost exclusively in the roots, with little expression in the stems (b) genes involved in dopamine synthesis were expressed in the later developmental stages (Supplementary Fig. 3-7) as well as the root and stem and (c) the remaining subclusters were a papavarine, thebaine and morphine genes that were highly expressed in the latex sample. Interestingly, several of the MLP and PR10 genes in unknown pathways clustered tightly with genes involved in morphine and thebaine production, further implicating their role in morphine biosynthesis and presenting potential candidates for functional studies.

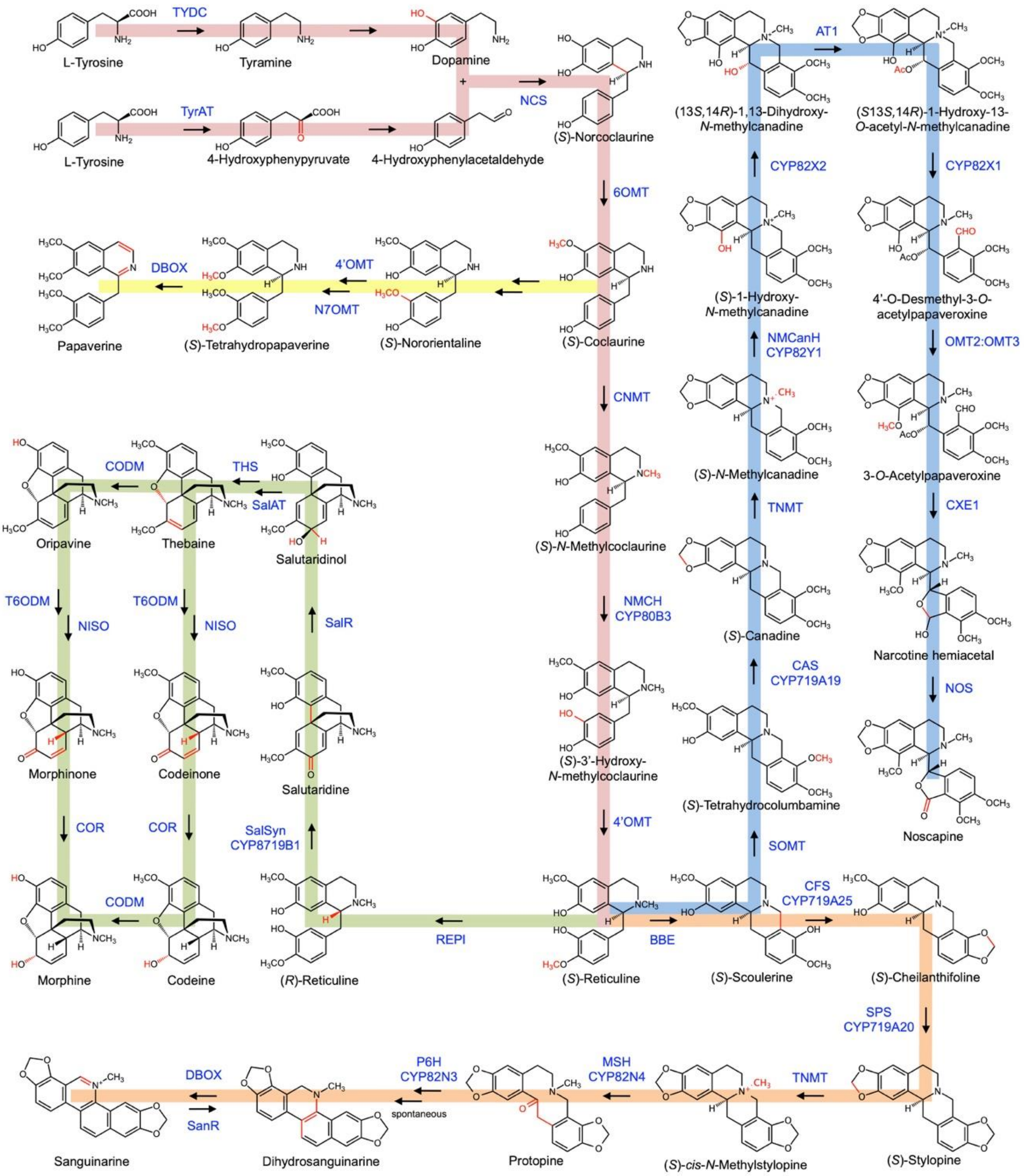

Supplementary Figure 1. Biosynthetic pathways leading to major benzylisoquinoline alkaloids accumulating in opium poppy. Pathways: *pink* 1-benzylisoquinoline "core" pathway; *green*, thebaine (promorphinan) and morphinan; *yellow*, papaverine; *blue*, noscapine (phthalideisoquinoline); *orange*, sanguinarine (benzo[*c*]phenanthridine). Enzymes for which corresponding genes have been isolated from opium poppy are shown in *blue*. Chemical conversions catalyzed by each enzyme are shown in *red*. *TYDC* tyrosine/DOPA decarboxylase, TyrAT tyrosine

aminotransferase, *NCS* norcoclaurine synthase, *6OMT* norcoclaurine 6-*O*-methyltransferase, *CNMT* coclaurine *N*-methyltransferase, *NMCH* *N*-methylcoclaurine 3′-hydroxylase (CYP80B3), *4′OMT2* 3′-hydroxyl-*N*-methylcoclaurine 4′-*O*-methyltransferase (isoform 2), *BBE* berberine bridge enzyme, *SOMT1* scoulerine 9-*O*-methyltransferase, *CAS* canadine synthase (CYP719A19), *TNMT* tetrahydroprotoberberine *N*-methyltransferase, *NMCanH* *N*-methylcanadine 1-hydroxylase (*CYP82Y1)*, *AT1* acetyltransferase 1, *OMT2:OMT3* 4'-*O*-desmethyl-3-*O*-acetylpapaveroxine *O*-methyltransfase heterodimer; *CXE1* carboxylesterase 1, *NOS* noscapine synthase, *CFS* cheilanthifoline synthase (CYP719A25), *SPS* stylopine synthase (CYP719A20), *MSH* *N*-methylstylopine 14-hydroxylase (CYP82N4), *P6H* protopine 6-hydroxylase (CYP82N3), *DBOX* dihydrosanguinarine oxidase, *SanR* sanguinarine reductase, REPI reticuline epimerase, *SalSyn* salutaridine synthase (CYP719B1), *SalR* salutaridine reductase, *SalAT* salutaridinol 7-*O*-acetyltransferase, THS thebaine synthase, *T6ODM* thebaine 6-*O*-demethylase, *NISO* neopinone isomerase, *COR* codeinone reductase, *CODM* codeine *O*-demethylase, *N7OMT* norreticuline 7-*O*-methyltransferase, *3′OHase* uncharacterized 3′-hydroxylase, *3′OMT* uncharacterized 3′-*O*-methyltransferase.

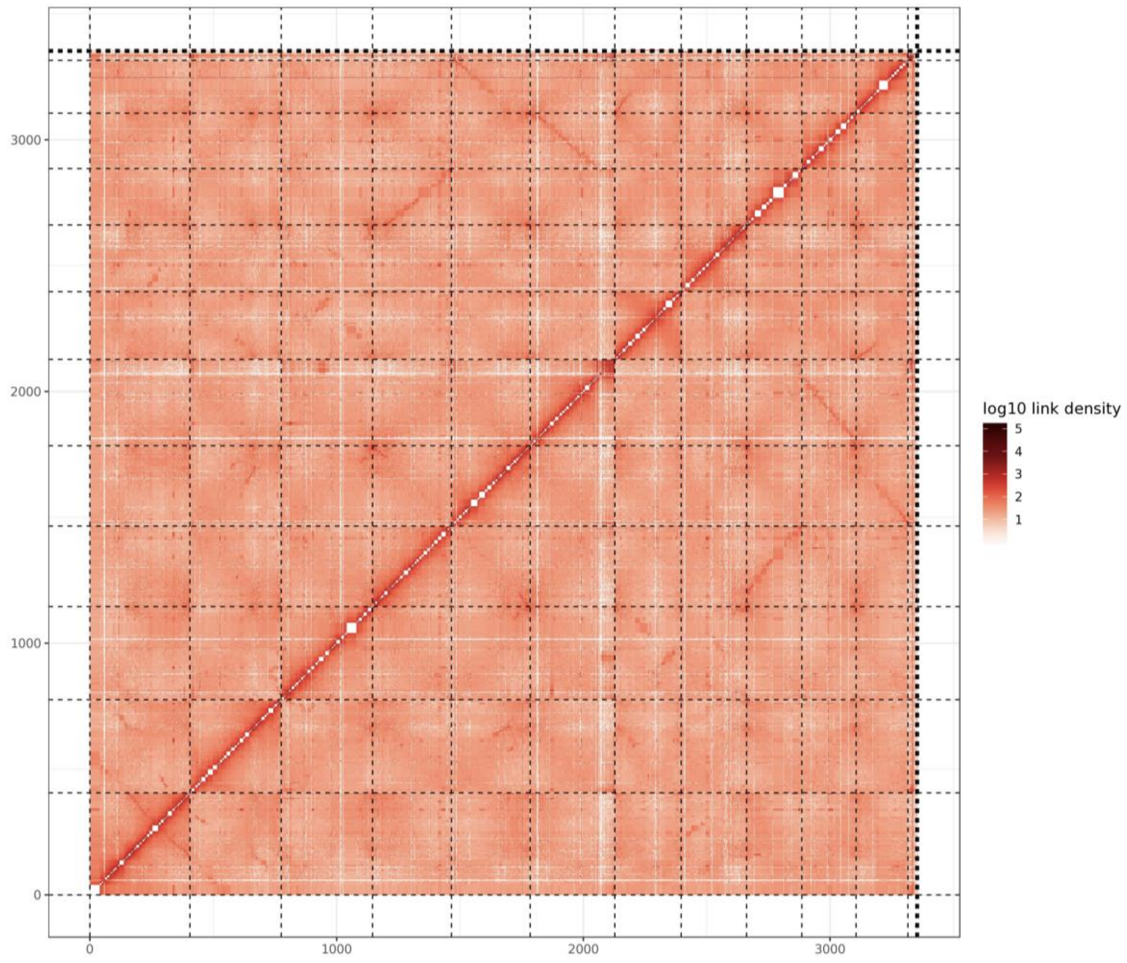

Supplementary Figure 2. Post-scaffolding Hi-C contact heatmap. Each x/y coordinate corresponds to 922,623bp of sequence, with the log10 of the Hi-C linkage density between regions indicated by an (x, y) coordinate pair displayed in red. Signal within contigs is masked to emphasize the Hi-C signal used to generate the scaffolds. Dashed lines indicate the boundaries between scaffolds. Three very short scaffolds (<1 Mb each) are visually compressed at the end of the figure, but are distinct in the underlying assembly.

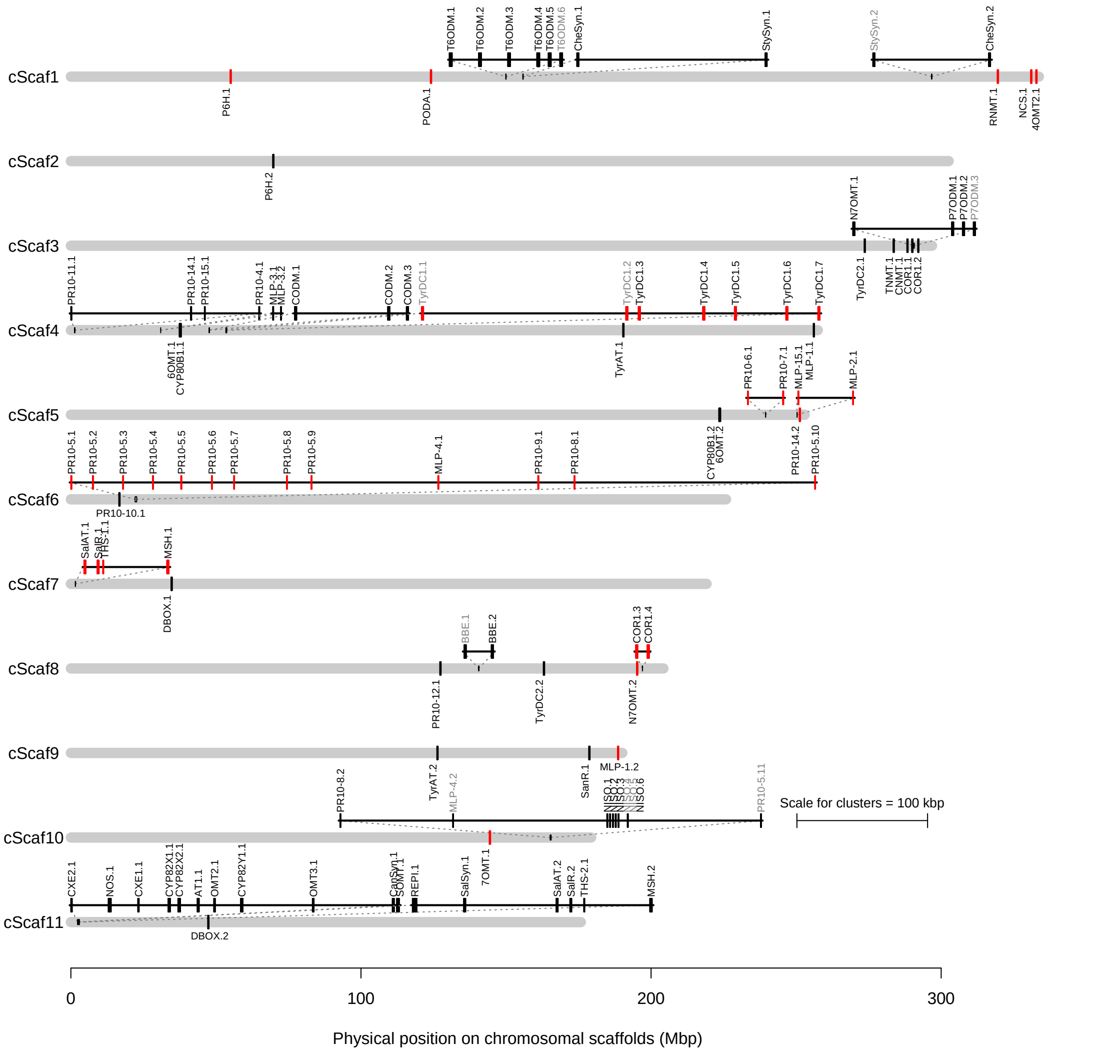

Supplementary Figure 3. Genomic organization of the benzylisoquinalone alkaloid pathway genes showing contigs that were previously unmapped but placed on cScafs by Hi-C. Genes successfully mapped to chromosome-scale scaffolds in the Guo *et al*.[2] genome are shown in black, with genes on previously unmapped contigs but mapped with Hi-C shown in red. Relationship between cScafs and *P. somniferum* chromosomes as defined by Guo *et al*.[2] is shown in Supplementary Table 12. Source data are provided as a Source Data file.

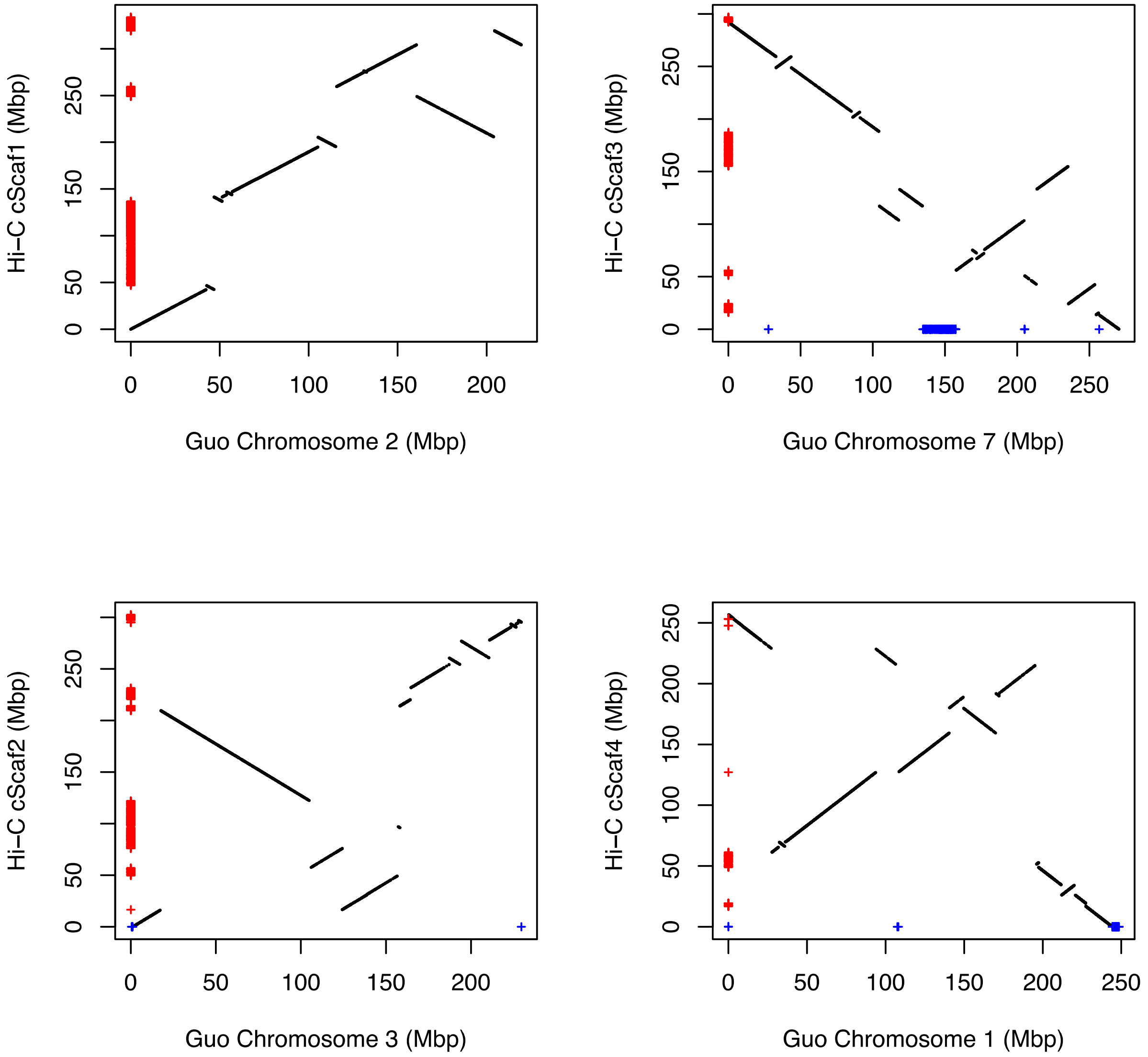

Hi−C cScaf1 (Mbp)
0
50
150
250
0
50
100
150
200
Guo Chromosome 2 (Mbp)
Hi−C cScaf3 (Mbp)
0
50
150
250
0
50
100
150
200
250
Guo Chromosome 7 (Mbp)
Hi−C cScaf2 (Mbp)
0
50
150
250
0
50
100
150
200
Guo Chromosome 3 (Mbp)
Hi−C cScaf4 (Mbp)
0
50
100
150
200
250
0
50
100
150
200
250
Guo Chromosome 1 (Mbp)

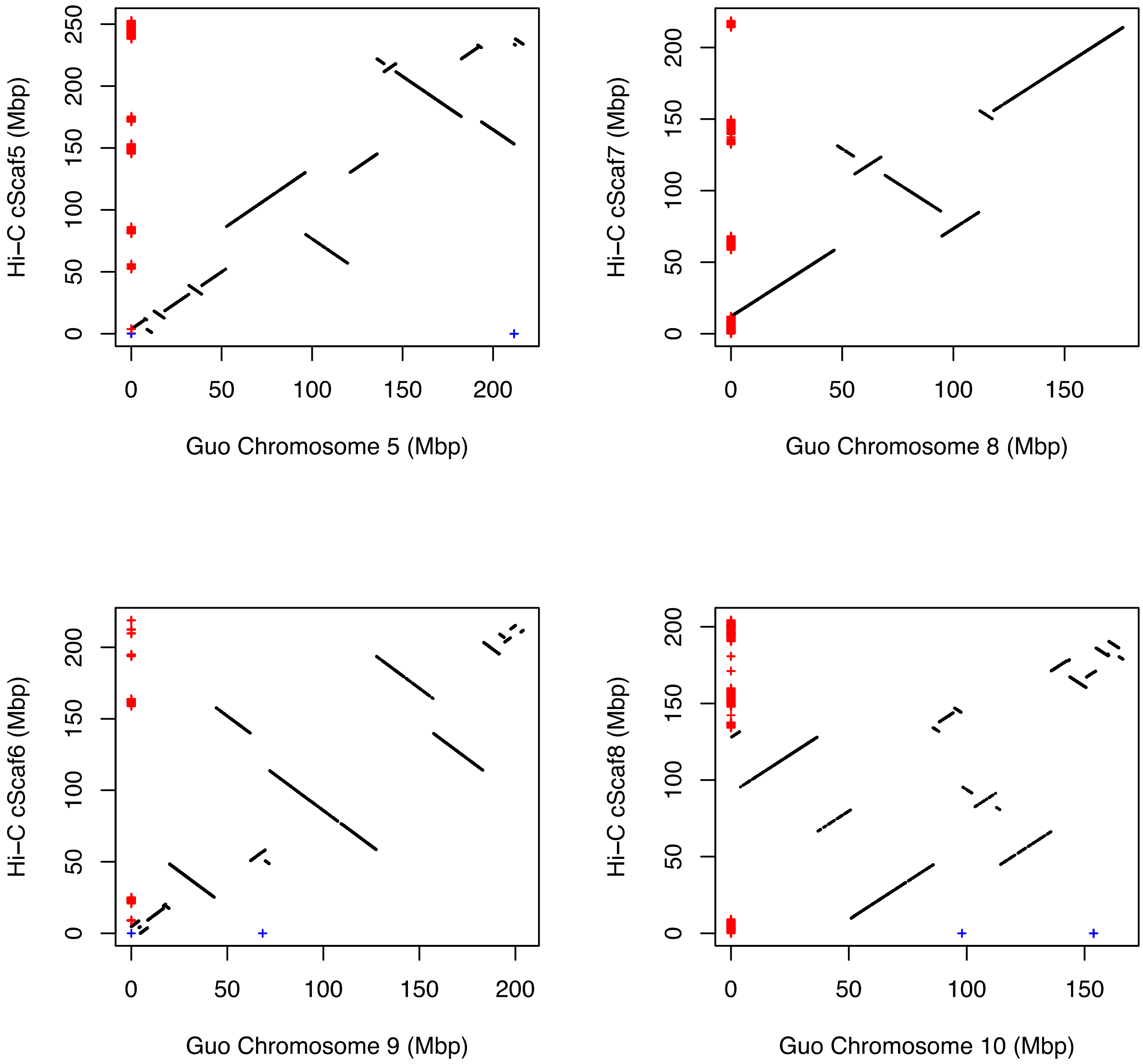

Hi−C cScaf5 (Mbp)
0
50
100
150
200
250
0
50
100
150
200
Guo Chromosome 5 (Mbp)
Hi−C cScaf7 (Mbp)
0
50
100
150
200
0
50
100
150
Guo Chromosome 8 (Mbp)
Hi−C cScaf6 (Mbp)
0
50
100
150
200
0
50
100
150
200
Guo Chromosome 9 (Mbp)
Hi−C cScaf8 (Mbp)
0
50
100
150
200
0
50
100
150
Guo Chromosome 10 (Mbp)

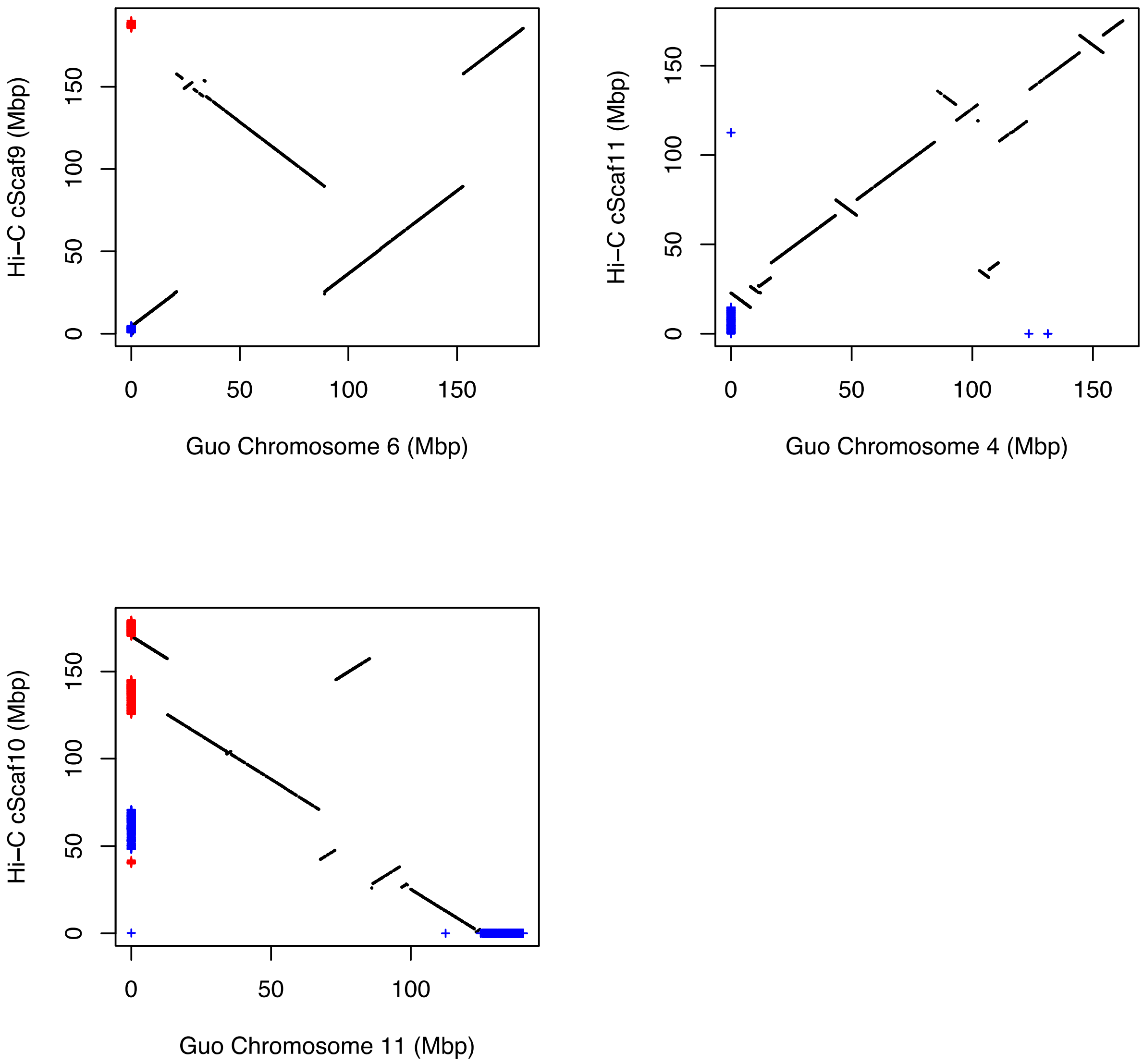

Supplementary Figure 4. Correspondence between the Guo *et al*.[2] assembly and the Hi-C assembly constructed here. Each dot represents a case where a given gene mapped to both genomes with 99% identity and 99% coverage. Black indicates cases where the gene was on the same chromosome-scale scaffold in each assembly, red indicates cases where genes were on an unscaffolded contig in the Guo assembly, but were scaffolded in the Hi-C assembly, and blue indicates cases where the gene was scaffolded in both assemblies but on different chromosome-scale scaffolds. Relationship between cScafs and *P. somniferum* chromosomes as defined by Guo *et al*.[2] is shown in Supplementary Table 12. Source data are provided as a Source Data file.

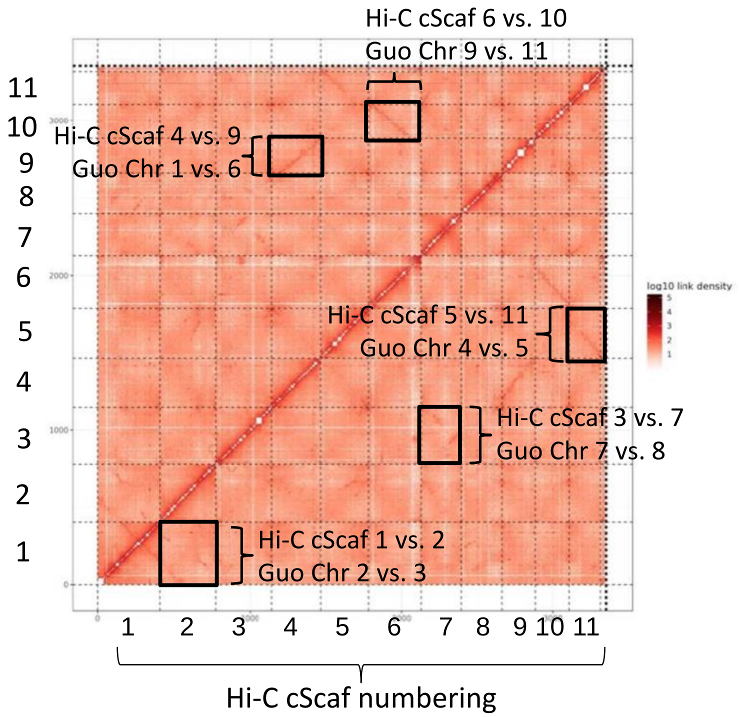

Supplementary Figure 5. Signals consistent with whole genome duplication in Hi-C data. Off diagonal correspondence is evident between some chromosomes, which correspond to the pairs of chromosomes that were identified by Guo *et al.*[2] as having been derived from an ancestral chromosome that underwent a whole-genome duplication, as per their Supplementary Fig. 11. Relationship between cScafs and *P. somniferum* chromosomes as defined by Guo *et al*.[2] is shown in Supplementary Table 12.

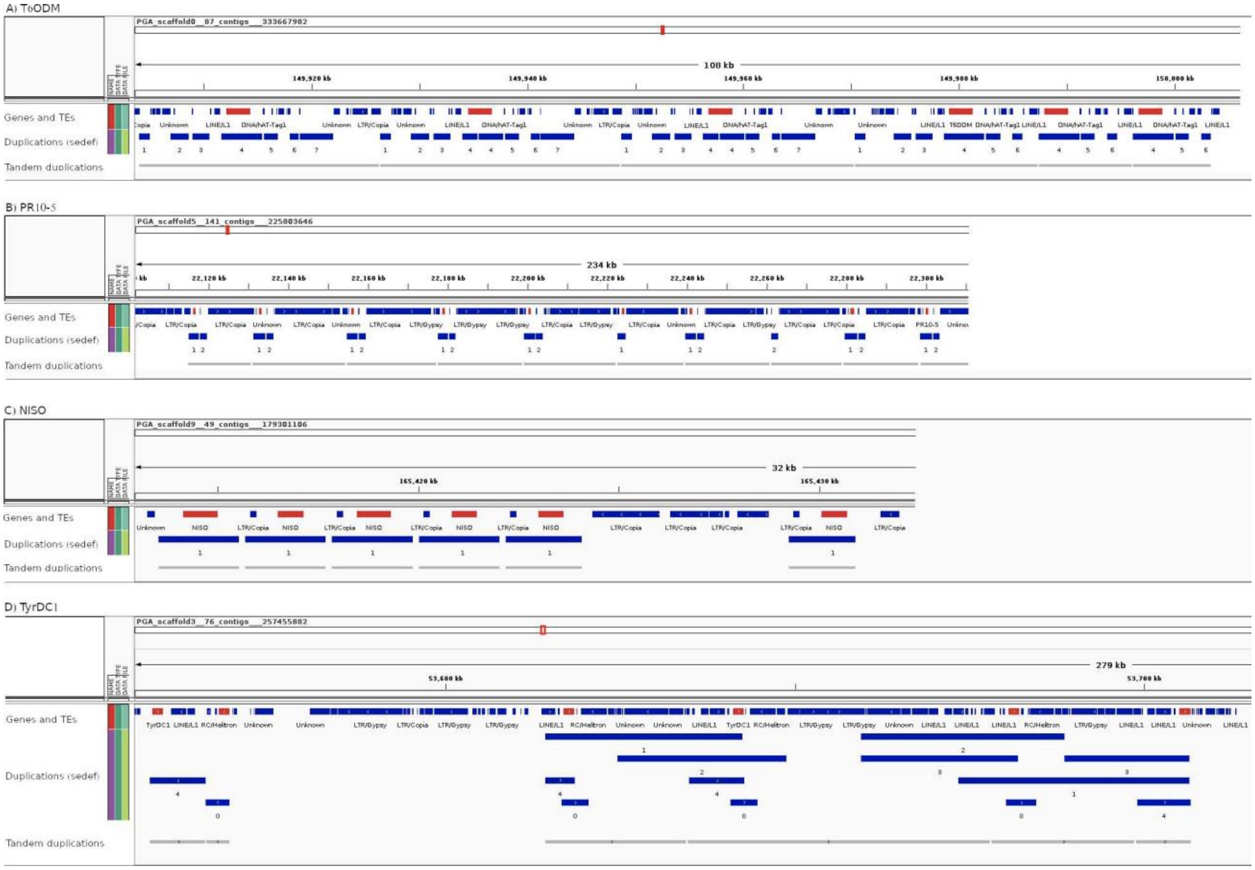

Supplementary Figure 6. Repetitive regions containing major BIA pathway genes. Tandem duplications are shown for A) T6ODM, B) PR10-5, C) NISO and D) TyrDC1. Red: Genes, blue: TEs and duplication annotation results from SEDEF, grey: putative complete direct duplications.

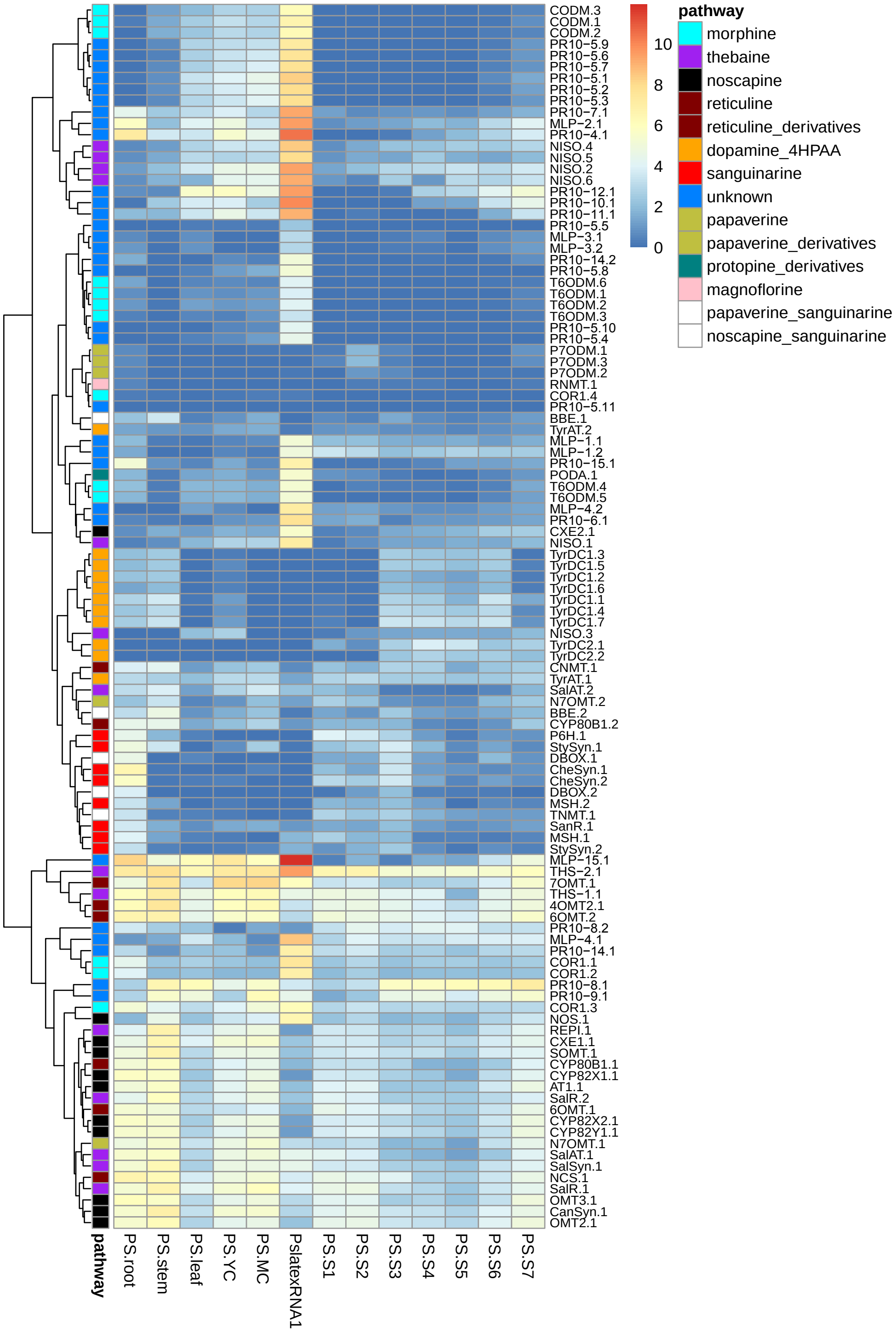

Supplementary Figure 7. Heatmap of gene expression of BIA pathway genes across six tissues and seven developmental stages. The heatmap colours range from 0 (blue) to 12 (red) of log1p[gene expression]. Source data are provided as a Source Data file.

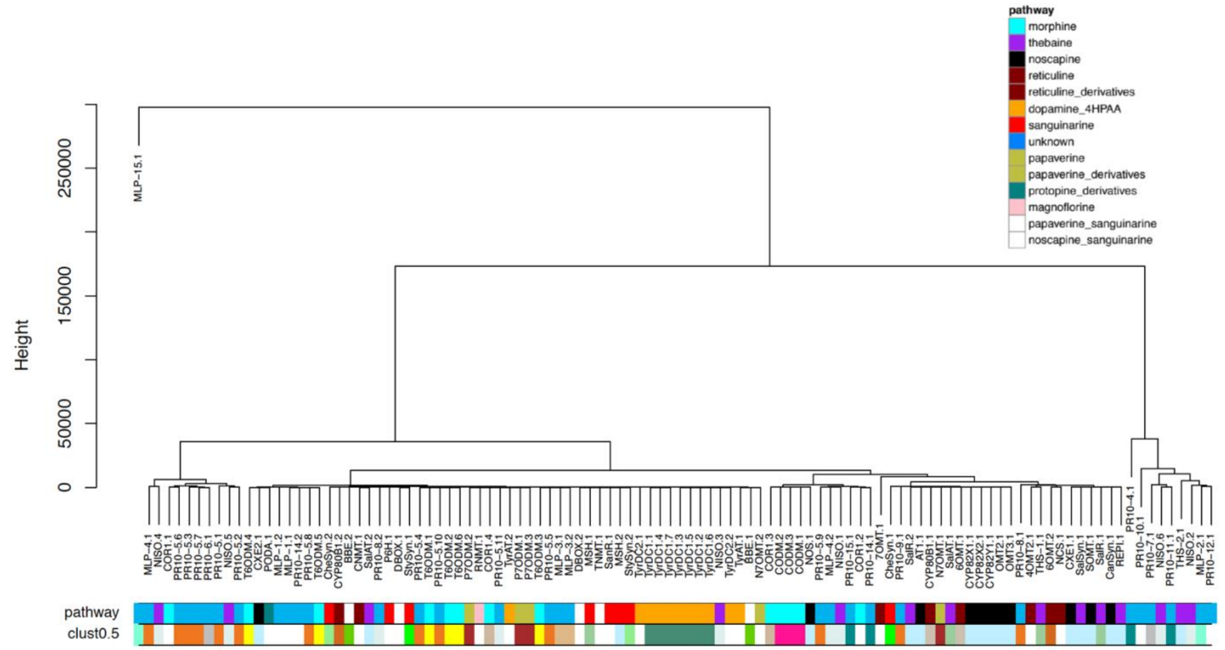

Supplementary Figure 8. Clustering dendrogram of gene expression of BIA pathway genes annotated with pathway membership and cluster allocation at 0.5 Mb clustering distance. Colours corresponding to pathways are depicted in the figure legend and clusters at clust0.5 are random. See Supplementary Table 6 for more information on clust0.5. Source data are provided as a Source Data file.

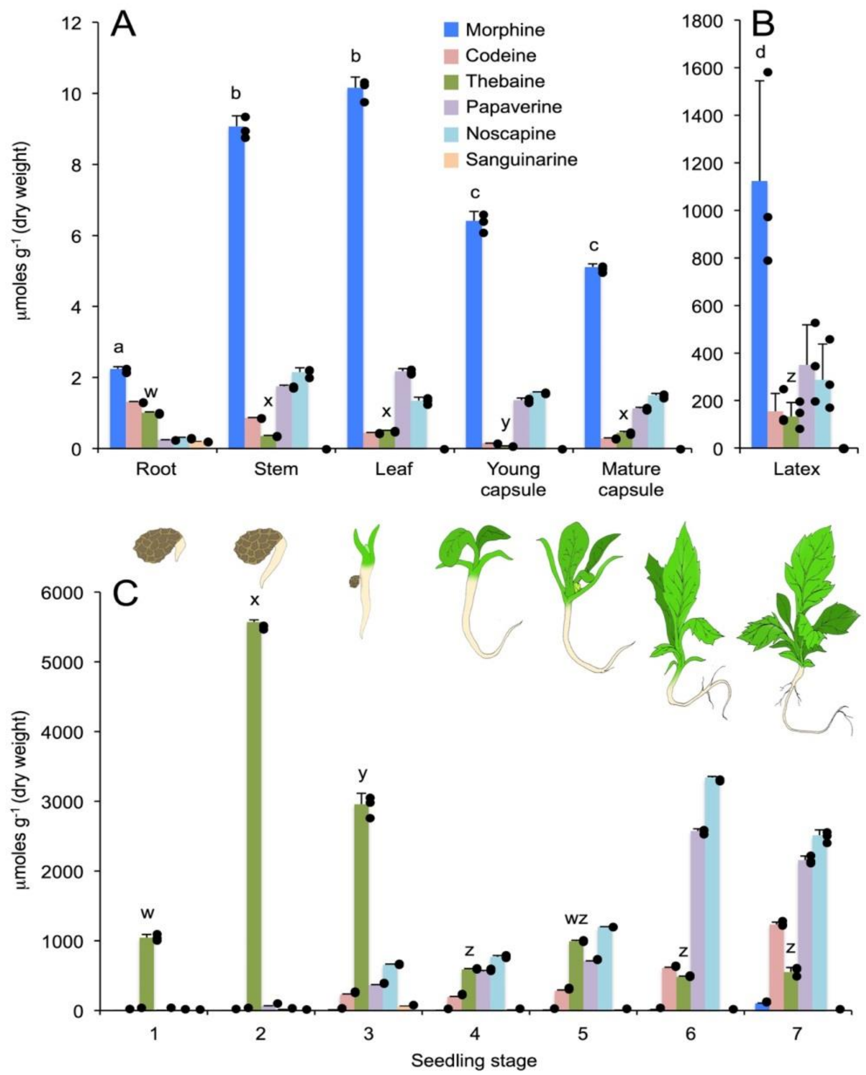

Supplementary Figure 9. Alkaloid quantities per dry weight of various plant components. (A-B) Content of six major alkaloids in various organs and in the latex of opium poppy. For plant organs (A) and latex (B), n=3 biologically independent samples were analyzed over two independent experiments producing similar results (only one experiment is included in the plot). Latex alkaloid content is shown separately owing to the substantial difference in y-axis scale compared to organs. (C) Profiles of six alkaloids in seven consecutive developmental stages of opium poppy seedlings. Seedling stages are numbered 1-7 on x-axis, with schematic diagrams shown above each stage. For seedlings, n=3 biologically independent samples were analyzed over two independent experiments producing similar results (only one experiment is included in the plot). In all cases, bars represent mean values +/- standard deviation, and individual data points are plotted to the right of each corresponding bar. Different letters above bars indicate significant differences ($P < 0.5$) as determined using an unpaired, two tailed Student t-test. For clarity, statistical analyses are shown only for morphine (letters a-d) and thebaine (letters w-z). Illustrations by Jill Hagel. Source data are provided as a Source Data file.

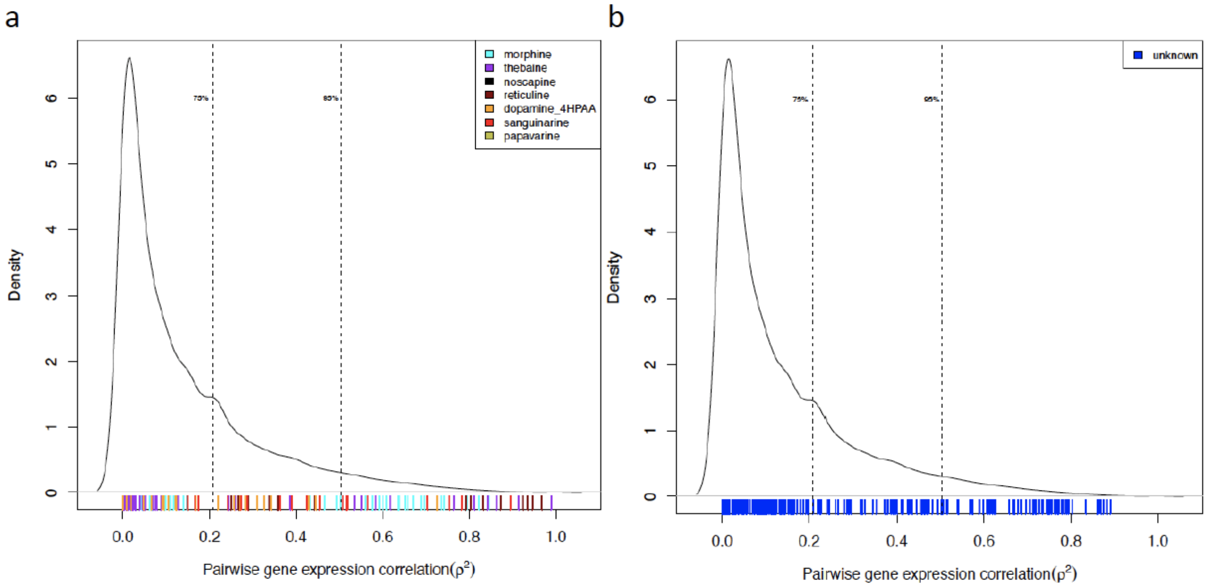

Supplementary Figure 10. Gene expression correlation of unlinked genes in the opium poppy transcriptome. (A) Density of correlation between unlinked non-BIA genes compared to unlinked genes in known BIA pathways and (B) genes in unknown pathways. Source data are provided as a Source Data file.

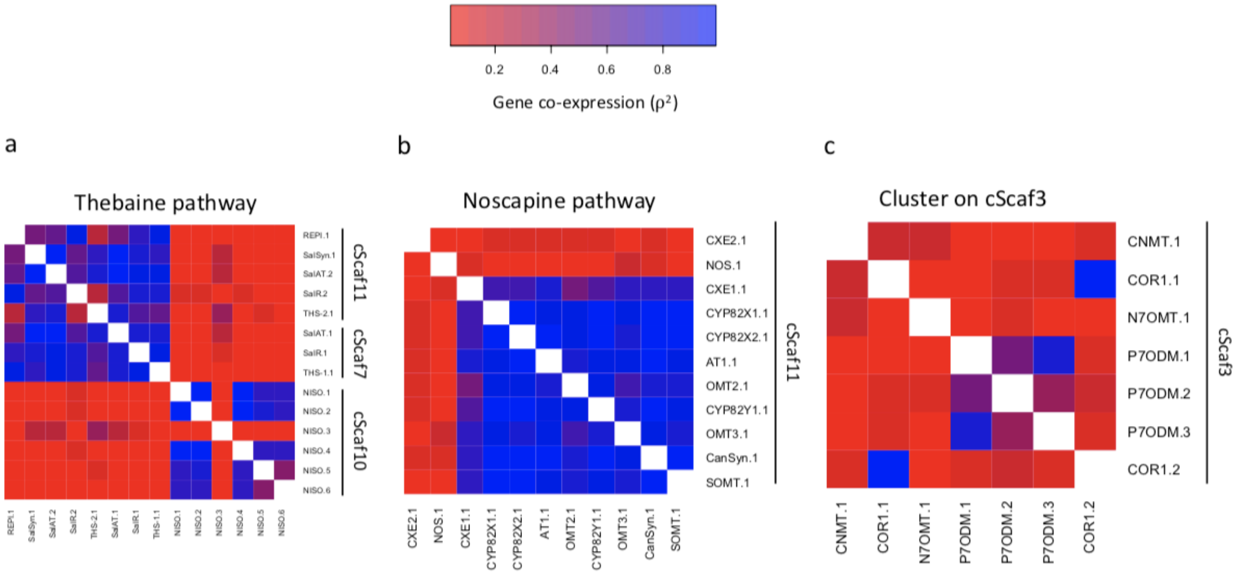

Supplementary Figure 11. Co-expression among the genes in the thebaine and noscapine pathways and in the cluster of BIA genes on cScaf 3. BIA genes shown for the thebaine (A) and noscapine (B) pathways, and a cluster of genes on cScaf3 (C). Shading of each cell represents the Spearman's correlation between TPM expression counts across the six tissues and seven developmental stages. Relationship between cScafs and *P. somniferum* chromosomes as defined by Guo *et al*.[2] is shown in Supplementary Table 12. Source data are provided as a Source Data file.

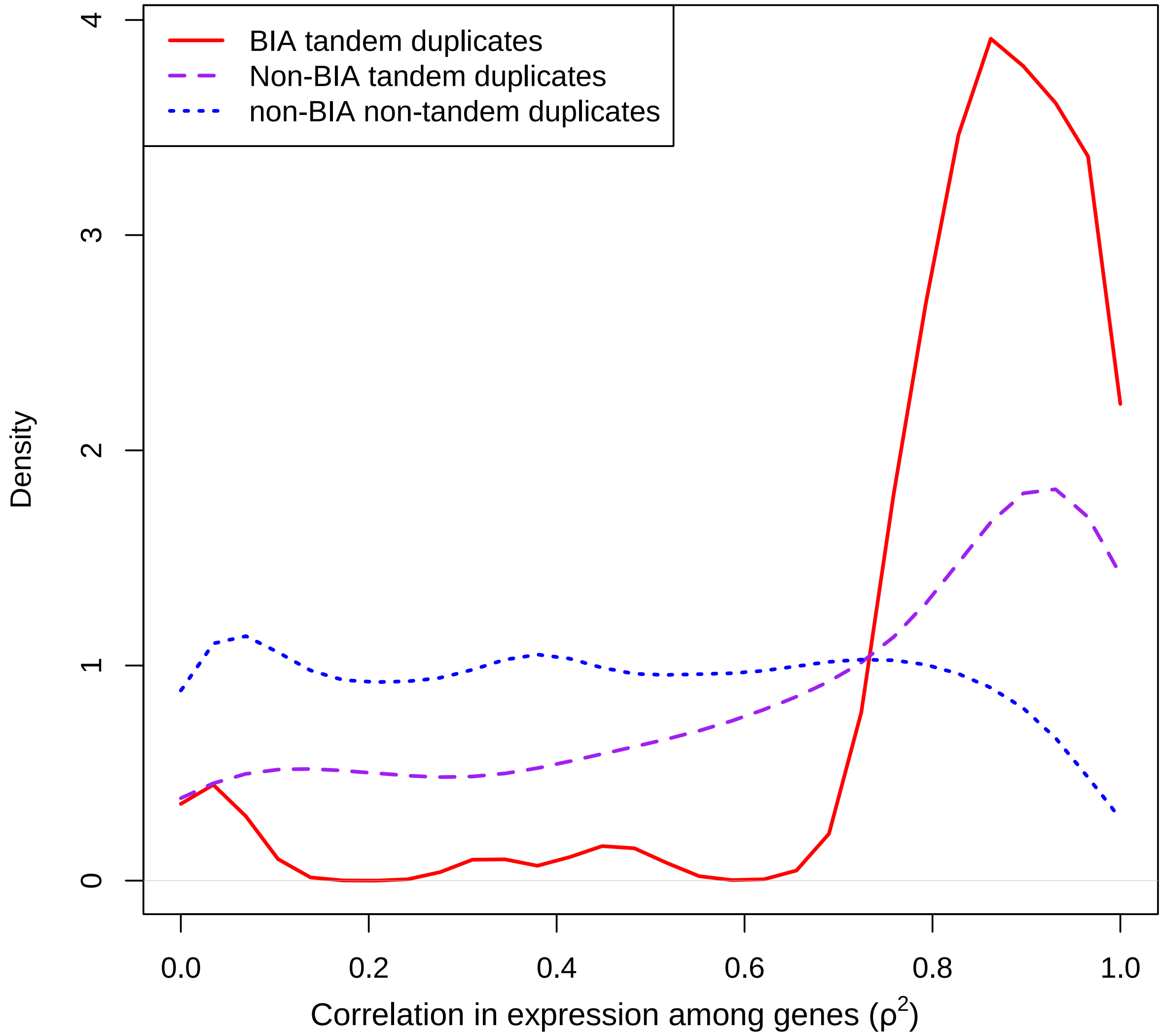

Supplementary Figure 12. Patterns of co-expression among BIA and non-BIA tandem duplicates. For comparison, values are also shown for co-expression for duplicated genes localized in different regions of the genome (either >500 kb or on different chromosomes). Co-expression is measured as Spearman's correlation between TPM expression counts across the six tissues and seven developmental stages. Source data are provided as a Source Data file.

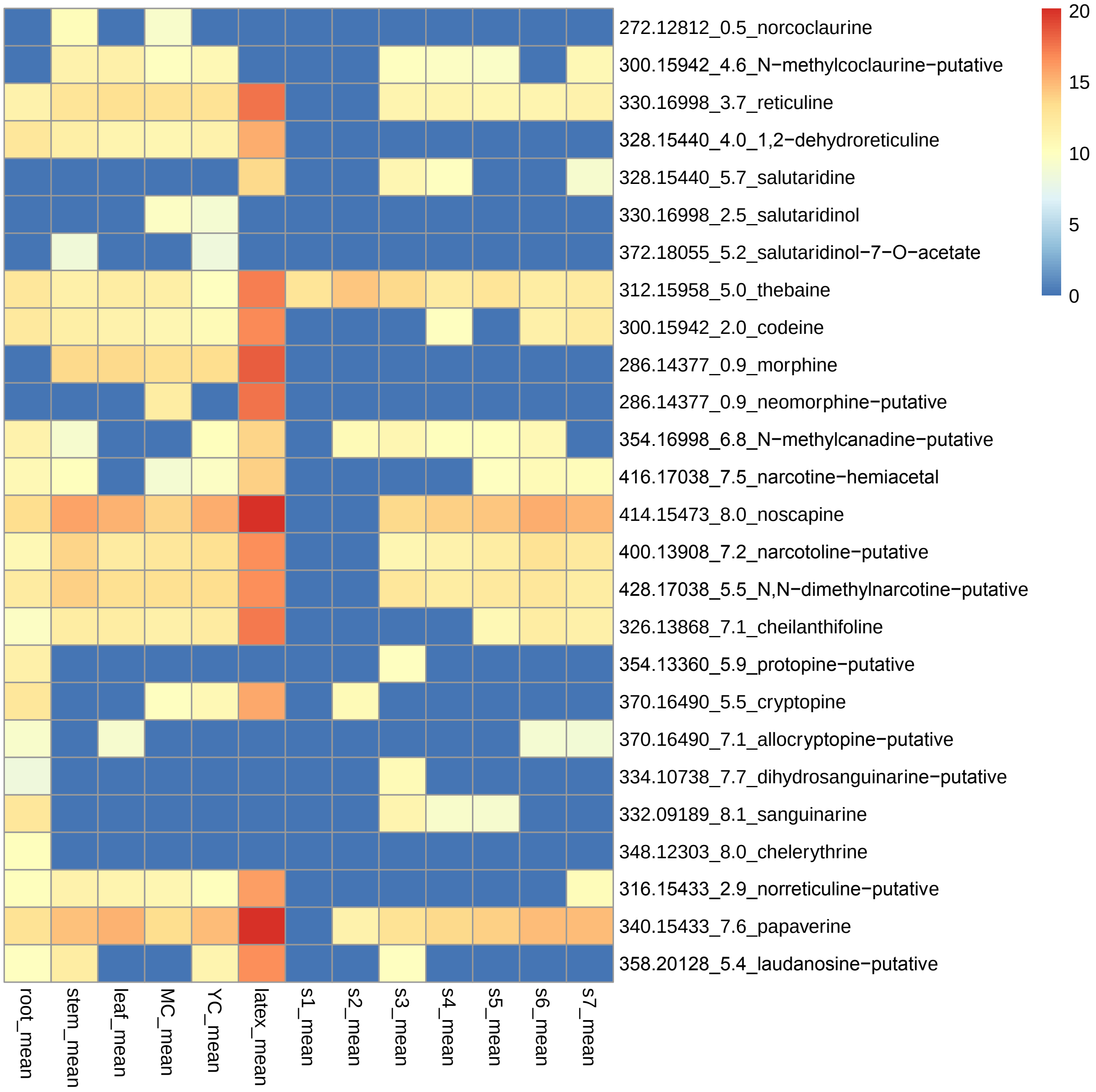

Supplementary Figure 13. Heatmap of known alkaloid abundances from six tissues and seven developmental stages quantified from Orbitrap analysis. The heatmap colours range from 0 (blue) to 20 (red) of log1p[alkaloid content]. Source data are provided as a Source Data file.

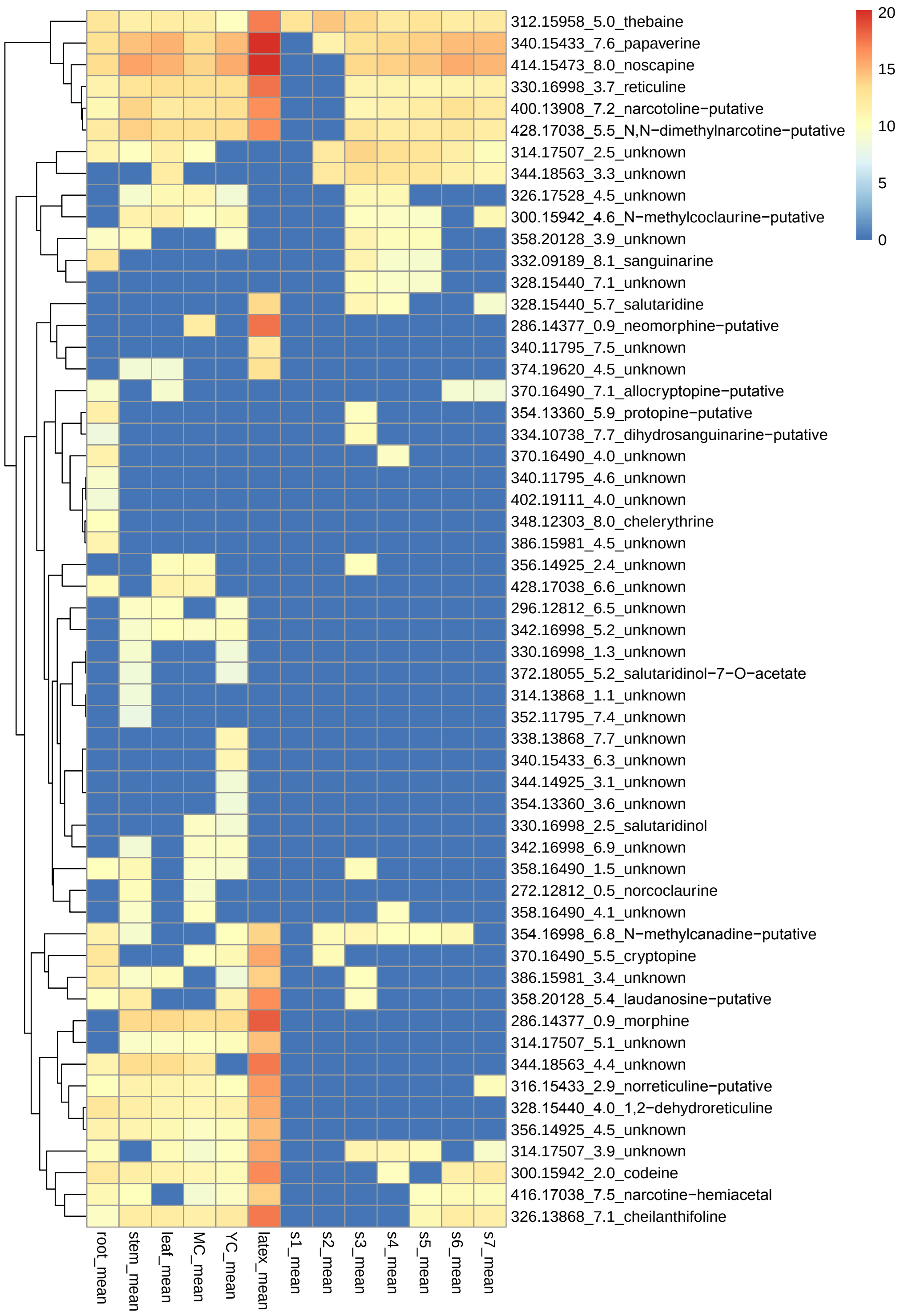

Supplementary Figure 14. Heatmap of all known and unknown alkaloid abundances from six tissues and seven developmental stages quantified from Orbitrap analysis. The heatmap colours range from 0 (blue) to 20 (red) of log1p[alkaloid content]. Source data are provided as a Source Data file.

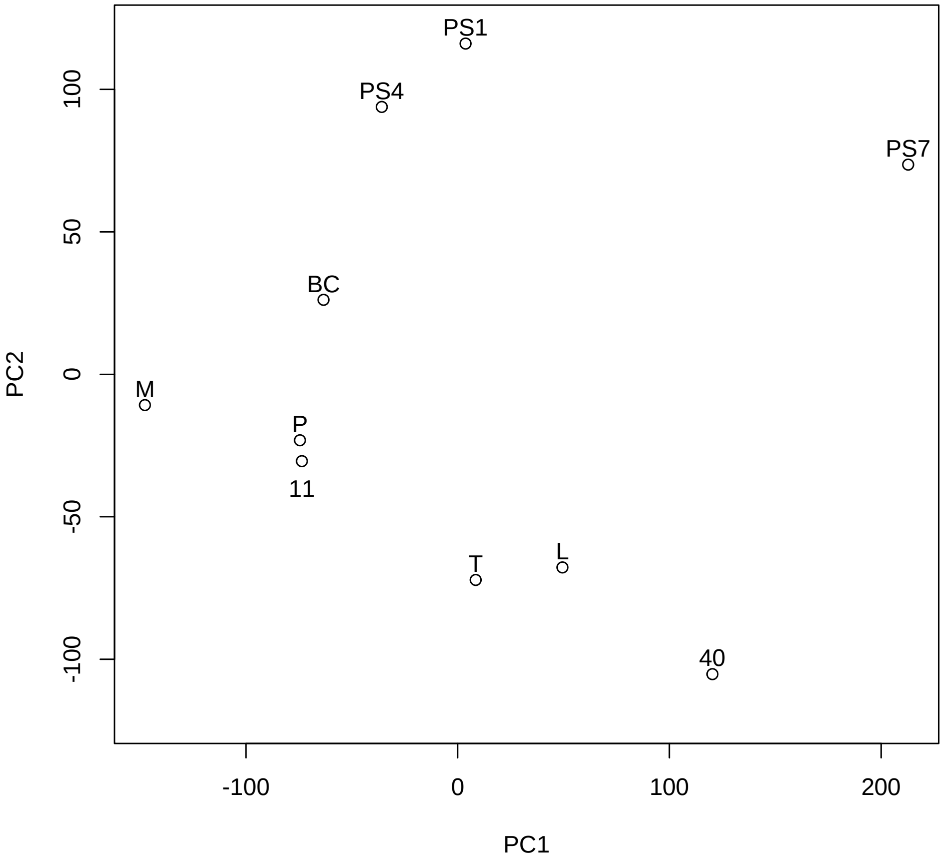

Supplementary Figure 15. Principal components analysis of copy number variation in the non-BIA genes identifies genome-wide similarity among cultivars. Analysis was run on 62,086 CNVs identified across the genome from non-BIA genes. Source data are provided as a Source Data file.

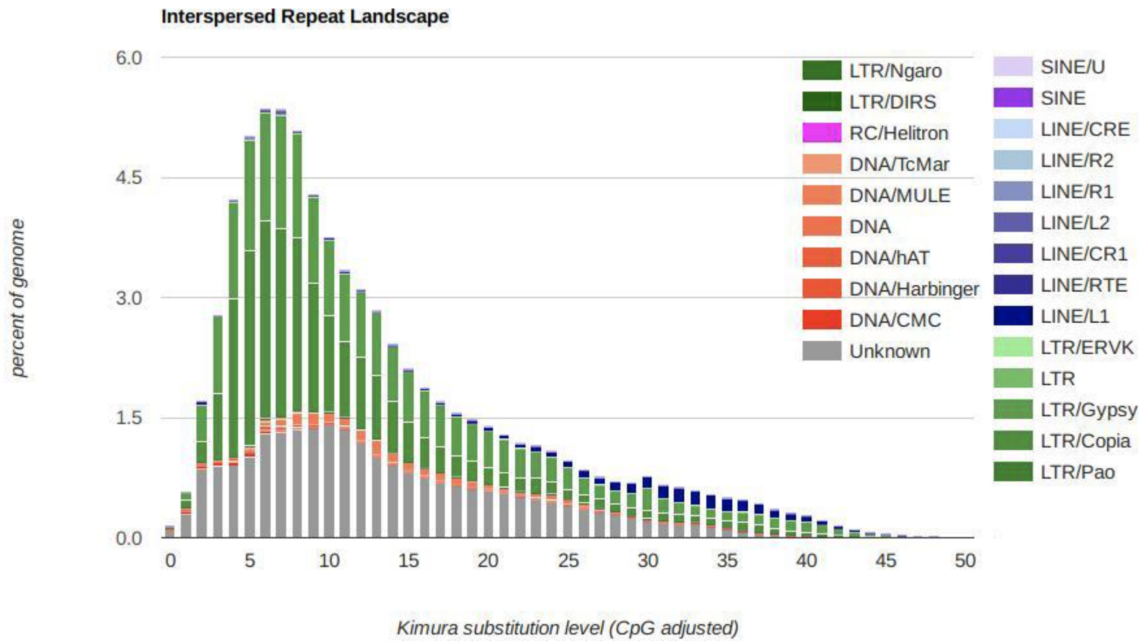

Supplementary Figure 16. Interspersed repeat landscape ordered by the Kimura substitution levels. Kimura distance provides an estimation of the age of the element insertion. Source data are provided as a Source Data file.

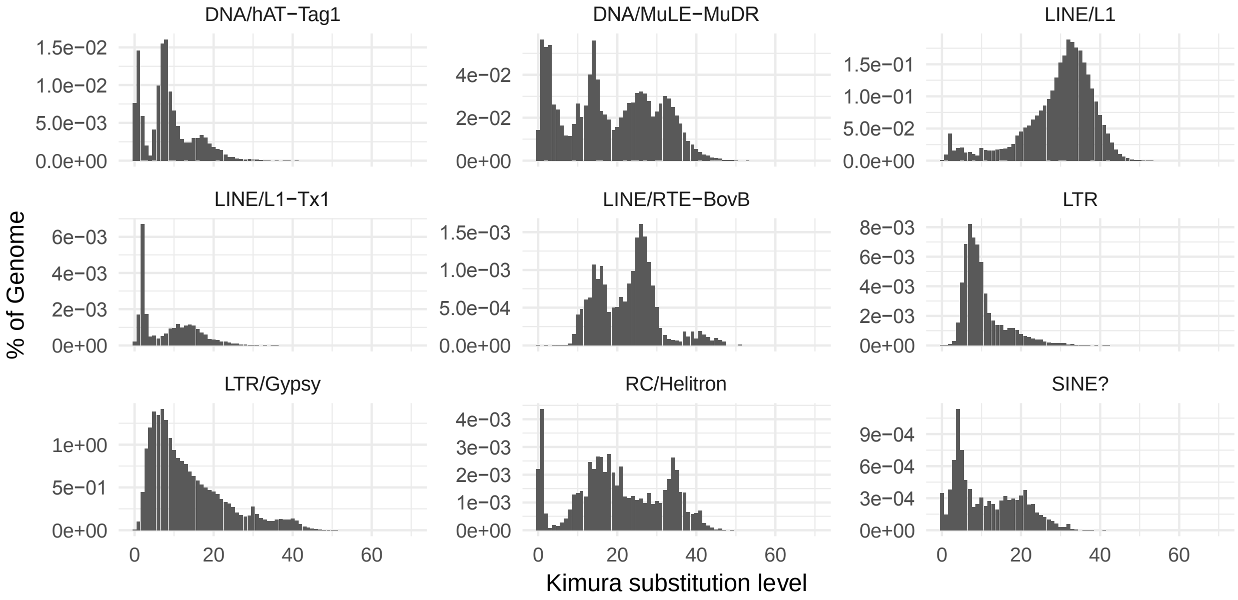

Supplementary Figure 17. Repeat landscapes for enriched TEs ordered by Kimura substitution level. Kimura distance provides an estimation of the age of the element insertion for the main TE classes identified as being enriched within at least on cluster (from Supplementary Table 10. Source data are provided as a Source Data file.

Supplementary Table 1. Improved assembly and annotation statistics of the opium poppy genome.

Number of contigs before Hi-C scaffolding	35732
Length of contig N50	7.6 Mb
Number of contig N50	104
Number of chromosome-scale scaffolds (cScafs)	11
Assembly size of cScafs	2.64 Gb
Number of all protein-coding genes (projected from Guo assembly)	63191

Supplementary Table 2. Statistics of the mapping results against the two genome assemblies with the Mate-Pair reads from 7-10kb jumping libraries of PS7 and HN1.

Source of the Mate-pair reads	Mapping	Guo (chr1-chr11)	Hi-C (chr1-chr11)	Guo_whole genome	Hi-C_whole genome
PS7 (~15x)	Properly paired	75.41%	85.18%	85.63%	85.60%
	With mate mapped to a different chromosome (mapQ>=5)	22991485	15652115	16053586	15619706
HN1 (~34.0x)	Properly paired	74.55%	85.05%	85.71%	85.67%
	With mate mapped to a different chromosome (mapQ>=5)	35676132	20268043	19650015	19154152

Supplementary Table 3. Mapping of PacBio reads across scaffold bridges in both genome assemblies.

	PS7 to Guo	HN1 to Guo	Both to Guo	PS7 to Hi-C	HN1 to Hi-C	Both to Hi-C
Total number of gaps	879	879	879	768	768	768
Number of gaps with reads mapping	38	45	34	185	199	146
Proportion of gaps	4.3%	5.1%	3.9%	24.1%	25.9%	19.0%

Note: Table shows the number of scaffold breaks in the Guo *et al.*[2] genome and the Hi-C assembly with at least one high-confidence PacBio read mapping across the gap, using libraries from PS7 and HN1 accessions (both > 10× coverage). Source data are provided as a Source Data file.

Supplementary Table 4. The effect of maximum distance to the nearest BIA gene neighbor on the amount of clustering in BIA genes.

Maximum distance to nearest neighbor (Mbp)	Number of genes in all clusters	Proportion of BIA genes in clusters	Number of clusters	Total size of all clusters (Mbp)	Proportion of genome occurring within clusters	*p*-value for global clustering test	*p*-value for incremental clustering test
0.001	0	0.00	0	0.0000	0.0000	NA	NA
0.0025	7	0.06	2	0.0147	0.0000	0	NA
0.005	9	0.08	3	0.0196	0.0000	0	0
0.0075	14	0.13	5	0.0428	0.0000	0	NA
0.01	27	0.25	10	0.1241	0.0000	0	0
0.02	40	0.37	15	0.2420	0.0001	0	NA
0.03	59	0.54	19	0.5964	0.0002	0	NA
0.04	62	0.57	19	0.7037	0.0003	0	NA
0.05	66	0.61	18	0.9254	0.0004	0	0
0.075	69	0.63	16	1.2319	0.0005	0	NA
0.1	76	0.70	17	1.7489	0.0007	0	0
0.2	81	0.74	17	2.4542	0.0009	0	NA
0.5	85	0.78	18	3.2656	0.0012	0	0
1	87	0.80	18	4.7634	0.0018	0	0.4284
5	93	0.85	19	16.1685	0.0061	0	0.1829
10	97	0.89	17	59.4910	0.0226	0	0.5556
20	99	0.91	14	121.1587	0.0459	0.052	NA
50	104	0.95	11	351.7468	0.1334	0.7449	0.6931
100	108	0.99	10	816.1021	0.3094	0.6254	0.8256

Note: The proportion of total genome size is calculated relative to the size of the 11 cScafs in the assembly. *P*-values are not corrected for multiple comparisons, and are for one-sided tests of the significance of observed clustering based on two approaches: the global approach randomizes gene locations and tests whether significantly more clustering is observed than under randomness at different thresholds; the incremental approach begins with the baseline clustering, randomizes the locations of remaining non-clustered genes, and tests whether significantly more clustering is observed under randomness for each additional distance (see Methods).

Supplementary Table 5. Effect of window size on clustering of BIA genes.

Maximum distance to nearest neighbor (Mbp)	Number of genes in all clusters	Proportion of BIA genes in clusters	Number of clusters	Total size of all clusters (Mbp)	Proportion of genome occurring within clusters	*p*-value for global clustering test	*p*-value for incremental clustering test
0.001	0	0.00	0	0.0000	0.0000	NA	NA
0.0025	2	0.03	1	0.0054	0.0000	0.0087	NA
0.005	4	0.05	2	0.0104	0.0000	0.0006	0
0.0075	6	0.08	3	0.0198	0.0000	0	NA
0.01	9	0.12	4	0.0418	0.0000	0	0.0003
0.02	14	0.18	5	0.0896	0.0000	0	NA
0.03	21	0.27	7	0.2125	0.0001	0	NA
0.04	24	0.31	8	0.2818	0.0001	0	NA
0.05	28	0.36	9	0.4159	0.0002	0	0
0.075	30	0.39	7	0.6512	0.0002	0	NA
0.1	38	0.49	10	1.0732	0.0004	0	0
0.2	43	0.56	11	1.6384	0.0006	0	NA
0.5	48	0.62	12	2.7307	0.0010	0	0.0002
1	50	0.65	12	4.2286	0.0016	0	0.46
5	56	0.73	14	14.4601	0.0055	0	0.3535
10	64	0.83	16	57.9647	0.0220	0	0.2155
20	67	0.87	14	119.6339	0.0454	0.0002	NA
50	72	0.94	11	350.2428	0.1328	0.3124	0.6711
100	76	0.99	10	814.9016	0.3089	0.4389	0.8284

Note: Statistics are shown for number of clusters and the inclusion of BIA genes when putative tandem duplicates are removed (all tests as per Supplementary Table 4).

Supplementary Table 6. Effect of spatial scale of analysis on clustering of BIA genes.

	Number of genes in cluster	Clustering distance								
		0.1	0.2	0.5	1	5	10	20	50	100
dopamine_4HPAA	5	0.00	0.00	0.00	0.00	0.00	0.40	0.40	0.60	1.00
morphine	4	0.00	0.00	0.00	0.25	0.50	1.00	1.00	1.00	1.00
noscapine	13	0.85	0.85	0.85	0.85	0.92	0.92	1.00	1.00	1.00
papaverine	5	0.40	0.40	0.40	0.40	0.60	0.60	0.60	1.00	1.00
reticuline	8	0.00	0.00	0.50	0.50	0.88	0.88	0.88	1.00	1.00
sanguinarine	13	0.31	0.46	0.46	0.46	0.54	0.62	0.69	0.85	0.92
thebaine	9	0.89	1.00	1.00	1.00	1.00	1.00	1.00	1.00	1.00
unknown	22	0.59	0.68	0.73	0.77	0.77	0.91	0.95	0.95	1.00
p-value		0.0005	0.0005	0.0005	0.0010	0.0005	0.0380	0.0185	0.0620	0.0005

Note: Table shows the proportion of genes in each pathway that are clustered with any other BIA gene, based on clustering distance. Bottom row shows the *p*-values for the χ^2 test of significance at each clustering distance.

Supplementary Table 7. Effect of spatial scale of analysis on clustering of BIA genes by pathway.

	Number of genes in cluster	Clustering distance								
		0.1	0.2	0.5	1	5	10	20	50	100
dopamine_4HPAA	5	0.00	0.00	0.00	0.00	0.00	0.00	0.00	0.00	0.40
morphine	4	0.00	0.00	0.00	0.00	0.00	0.00	0.00	0.00	0.00
noscapine	13	0.85	0.85	0.85	0.85	0.85	0.85	0.85	0.85	0.85
papaverine	5	0.40	0.40	0.40	0.40	0.40	0.40	0.40	0.40	0.40
reticuline	8	0.00	0.00	0.50	0.50	0.75	0.75	0.75	0.75	0.75
sanguinarine	13	0.15	0.31	0.31	0.31	0.31	0.31	0.31	0.62	0.69
thebaine	9	0.89	0.89	0.89	0.89	0.89	0.89	0.89	0.89	0.89
unknown	22	0.59	0.68	0.73	0.77	0.77	0.82	0.82	0.86	0.91
p-value		0.0005	0.0005	0.0010	0.0010	0.0005	0.0005	0.0005	0.0020	0.0120

Note: Table shows the proportion of genes in each pathway that are clustered with other genes in the same pathway, based on clustering distance. Bottom row shows the *p*-values for the χ^2 test of significance at each clustering distance.

Supplementary Table 8. Summary of re-sequencing on 10 opium poppy cultivars.

Chemotype	Estimated genome size (Gb)	Coverage	Other name
PS7	2.84	26.11	Roxanne
T	3.06	16.81	-
M	3.05	18.53	Marianne
BC	3.06	19.77	Bea's choice
L	3.00	20.22	Louisianna
PS1	2.96	21.95	Natasha
PS4	2.96	22.46	Veronica
11	3.12	21.66	-
40	3.09	23.30	-
P	3.04	25.80	Przemko
average	3.02		

Supplementary Table 9. CNVs enrichment for pathway genes in clusters relative to isolated pathway genes.

Clustering distance (Mb)	Number of genes in clusters	Number of genes not in clusters	Proportion of genes in cluster with CNV	Proportion of genes not in cluster with CNV	*p*-value	*p*-value with noscopine cluster excluded
0.1	76	33	0.711	0.273	0.000021	0.000268
0.2	81	28	0.679	0.286	0.000281	0.002124
0.5	85	24	0.659	0.292	0.001299	0.006949
1	87	22	0.667	0.227	0.000193	0.001206
5	93	16	0.634	0.250	0.00403	0.013941
10	97	12	0.629	0.167	0.002227	0.007003

Note: The p-value is for a χ^2 test for CNVs in pathway genes in clusters compared isolated pathway genes.

Supplementary Table 10. Enrichment of transposable elements in clusters.

Name of TE	Scaffold	Cluster start	Cluster stop	*p*-value	# of TEs	Bonferroni-adjusted cutoff	Genes or pathways involved
LINE/L1	cScaf1	149912189	149998809	0.0020	24	0.0100	T6ODM
DNA/hAT-Tag1	cScaf1	149912189	149998809	0.0001	12	0.0100	T6ODM
SINE	cScaf1	149912189	149998809	0.0011	3	0.0100	T6ODM
LTR/Gypsy	cScaf4	1245660	1390104	0.0120	50	0.0125	PR10 genes
LINE/RTE-BovB	cScaf4	37313304	37633765	0.0032	3	0.0083	6OMT, CYP80B1
RC/Helitron	cScaf4	53401724	53706630	0.0002	17	0.0063	TyrDC1
LINE/L1	cScaf4	53401724	53706630	0.0017	51	0.0063	TyrDC1
LINE/L1-Tx1	cScaf5	223293068	223555031	0.0019	3	0.0063	6OMT, CYP80B1
LTR	cScaf7	1513969	1578993	0.0085	3	0.0125	Thebaine cluster
DNA/hAT-Tag1	cScaf7	1513969	1578993	0.0070	3	0.0125	Thebaine cluster
DNA/MuLE-MuDR	cScaf8	197013213	197023633	0.0143	5	0.0250	COR

Note: Tests are conducted for clusters showing significant enrichment of one or more transposable elements (TEs) at copy number >=3, after Bonferroni correction for multiple comparisons, and *p*-values shown are for a one-sided test. Relationship between cScafs and *P. somniferum* chromosomes as defined by Guo *et al*.[2] is shown in Supplementary Table 12.

Supplementary Table 11. Mean age of all TEs in each cluster and *p*-value from T-test of significance.

cScaffold	Cluster start	Cluster stop	Number of TEs	*p*-value	Mean age in cluster
cScaf1	149912189	149998809	114	0.3106	17.5
cScaf1	155761464	155906984	116	0.0012	19.7
cScaf1	296685802	296775816	110	0.1430	18.0
cScaf11	2268808	2932559	624	0.4560	16.9
cScaf3	290603899	290697657	106	0.1332	17.8
cScaf4	1245660	1390104	155	0.0001	19.7
cScaf4	30930417	30937119	8	0.4504	19.5
cScaf4	37313304	37633765	257	0.2711	17.3
cScaf4	47639429	47726604	77	0.0330	19.3
cScaf4	53401724	53706630	268	0.0004	19.0
cScaf5	223293068	223555031	237	0.4236	17.1
cScaf5	239431287	239458931	25	0.0069	21.8
cScaf5	250291029	250333482	44	0.0751	19.4
cScaf6	22116151	22685996	522	0.0001	18.5
cScaf7	1513969	1578993	86	0.0086	19.3
cScaf8	140581640	140604055	27	0.1456	19.8
cScaf8	197013213	197023633	22	0.0513	22.0
cScaf10	165210099	165532548	236	0.0000	19.5

Note: *p*-values shown are for a two-sided test. Genome-wide TEs have a mean age of 16.6. Relationship between cScafs and *P. somniferum* chromosomes as defined by Guo *et al*.[2] is shown in Supplementary Table 12.

Supplementary Table 12. The relationship between cScaf number and Guo *et al.*[2] chromosome number.

Guo *et al.*[2] chromosome number	Hi-C cScaf number	Guo *et al.*[2] scaffold name
2	1	NC_039359.1
3	2	NC_039360.1
7	3	NC_039364.1
1	4	NC_039358.1
5	5	NC_039362.1
9	6	NC_039366.1
8	7	NC_039365.1
10	8	NC_039367.1
6	9	NC_039363.1
11	10	NC_039368.1
4	11	NC_039361.1

Supplementary References

1. Li, H. Minimap2: pairwise alignment for nucleotide sequences. *Bioinformatics* **34**, 3094–3100 (2018).
2. Guo, L. *et al.* The opium poppy genome and morphinan production. *Science* **362**, 343-347 (2018).
3. Song, B. *et al.* Complement genome annotation lift over using a weighted sequence alignment strategy. *Front. Genet.* doi: 10.3389/fgene.2019.01046 (2019).
