# Supplementary material for: Gene clustering and copy number variation in alkaloid metabolic pathways of opium poppy
Source: Nat Commun. 2020 Mar 4;11:1190. doi: 10.1038/s41467-020-15040-2 (PMC7055283; doi:10.1038/s41467-020-15040-2)
Supplement: Supplementary file 3 — Reporting Summary [file 41467_2020_15040_MOESM3_ESM.pdf]

nature research

Corresponding author(s): Sam Yeaman

Last updated by author(s): 20/11/31

Reporting Summary

Nature Research wishes to improve the reproducibility of the work that we publish. This form provides structure for consistency and transparency in reporting. For further information on Nature Research policies, see Authors & Referees and the Editorial Policy Checklist .

Statistics

For all statistical analyses, confirm that the following items are present in the figure legend, table legend, main text, or Methods section.

n/a	Confirmed	
☐	☒	The exact sample size (n) for each experimental group/condition, given as a discrete number and unit of measurement
☐	☒	A statement on whether measurements were taken from distinct samples or whether the same sample was measured repeatedly
☐	☒	The statistical test(s) used AND whether they are one- or two-sided<br>*Only common tests should be described solely by name; describe more complex techniques in the Methods section.*
☐	☒	A description of all covariates tested
☐	☒	A description of any assumptions or corrections, such as tests of normality and adjustment for multiple comparisons
☐	☒	A full description of the statistical parameters including central tendency (e.g. means) or other basic estimates (e.g. regression coefficient) AND variation (e.g. standard deviation) or associated estimates of uncertainty (e.g. confidence intervals)
☐	☒	For null hypothesis testing, the test statistic (e.g. F, t, r) with confidence intervals, effect sizes, degrees of freedom and P value noted<br>*Give P values as exact values whenever suitable.*
☒	☐	For Bayesian analysis, information on the choice of priors and Markov chain Monte Carlo settings
☒	☐	For hierarchical and complex designs, identification of the appropriate level for tests and full reporting of outcomes
☐	☒	Estimates of effect sizes (e.g. Cohen's d, Pearson's r), indicating how they were calculated

Our web collection on statistics for biologists contains articles on many of the points above.

Software and code

Policy information about availability of computer code

Data collection	Please see methods for a full description of analysis tools used. Most are open source standard bioinformatics tools, with custom R scripts used for data analysis. These were deposited on the Dryad archive here https://doi.org/10.5061/dryad.m0cfxpnz9
Data analysis	Data analysis scripts are deposited on Dryad: https://doi.org/10.5061/dryad.m0cfxpnz9<br>Software and versions used in the manuscript were:<br>Kmergenie (v 1.7048)<br>GCE (v.1.0.0)<br>BWA-MEM (v0.7.12)<br>samtools (v1.5, with htslib 1.5)<br>bedtools (v2.27.1)<br>BioEdit (v7.1)<br>Juicebox Assembly Tool (v1.9.1)<br>LACHESIS (v2017)<br>SAMBLASTER<br>GCE (v.1.0.0)<br>Kmergenie (v 1.7048)<br>SEDEF (v.1.1-23)<br>RepeatMasker (v.4.0.7)<br>Maker-P<br>samtools (v1.3.1)<br>CNVnator (v0.3.2)<br>FastQC (v 0.11.7)<br>Hisat2 (v2.1.0)

Stringtie (v1.3.5)
Thermo X-Calibur (v3.1)
gmap (v2017-06-20)
BLAST (v2.5.0+)
htslib (v1.5)
GTH (v1.7.0)
fastp (v0.19.5)
Trimmomatic (v0.38)

For manuscripts utilizing custom algorithms or software that are central to the research but not yet described in published literature, software must be made available to editors/reviewers. We strongly encourage code deposition in a community repository (e.g. GitHub). See the Nature Research guidelines for submitting code & software for further information.

Data

Policy information about availability of data

All manuscripts must include a data availability statement. This statement should provide the following information, where applicable:
- Accession codes, unique identifiers, or web links for publicly available datasets
- A list of figures that have associated raw data
- A description of any restrictions on data availability

Genome assembly and other data, including alkaloid content, RNA-seq read counts and Hi-C contact matrices, which are necessary to generate all of the figures, tables, and datapoints are available on Dryad archive: https://doi.org/10.5061/dryad.m0cfxpnz9
Sequence reads are deposited here: NCBI Sequence Read Archive under BioProject PRJNA508405

Field-specific reporting

Please select the one below that is the best fit for your research. If you are not sure, read the appropriate sections before making your selection.

[x] Life sciences   [ ] Behavioural & social sciences   [ ] Ecological, evolutionary & environmental sciences

For a reference copy of the document with all sections, see nature.com/documents/nr-reporting-summary-flat.pdf

Life sciences study design

All studies must disclose on these points even when the disclosure is negative.

Sample size

There were no power analyses done before any sequencing. For the re-sequencing, we focused on maximizing the number of individuals we could sequence given the available budget and accessions available.
For the metabolite analysis, the selection of sample sizes was based on extensive experience with respect to the variation inherent to each of the experimental systems described in our manuscript. For these analyses, 3 fully independent biological replicates (i.e. individual plants) were used, and the experiments were conducted at two different times with similar results. Data was included for only one of the experiments to avoid confounding technical and biological replicates.

Data exclusions

No data were excluded from any analysis except for as described in "sample size" query above, where only one of two technical replicates was included from the metabolite analysis, although similar results were found in both cases.

Replication

As described above, metabolite quantification experiments were conducted twice with similar results, so only one was included. For all sequencing analysis and sampling, no replication was performed that was not documented in the paper.

Randomization

For metabolite analysis, plants from each of the experimental groups were selected randomly. Plants grown for transcriptome, Hi-C, and shotgun sequencing were randomly selected from available seeds.

Blinding

For all sequence related analysis (RNAseq, shotgun re-sequencing), there was no blinding, as the people running the analysis were aware of which treatment or experimental group each sequence came from. However, we do not feel that this is relevant for this particular application, as there is limited potential for the experimenter to affect the data when the same computational approach was used for all samples.
For the metabolite analysis, there was also no blinding, but again, as this is a highly automated process, there was no opportunity for the operator to have any biasing influence on the outcome.

Reporting for specific materials, systems and methods

We require information from authors about some types of materials, experimental systems and methods used in many studies. Here, indicate whether each material, system or method listed is relevant to your study. If you are not sure if a list item applies to your research, read the appropriate section before selecting a response.

Materials & experimental systems

n/a	Involved in the study
☒	☐ Antibodies
☒	☐ Eukaryotic cell lines
☒	☐ Palaeontology
☒	☐ Animals and other organisms
☒	☐ Human research participants
☒	☐ Clinical data

Methods

n/a	Involved in the study
☒	☐ ChIP-seq
☒	☐ Flow cytometry
☒	☐ MRI-based neuroimaging
