# Supplementary material for: Gene clustering and copy number variation in alkaloid metabolic pathways of opium poppy
Source: Nat Commun. 2020 Mar 4;11:1190. doi: 10.1038/s41467-020-15040-2 (PMC7055283; doi:10.1038/s41467-020-15040-2)
Supplement: Supplementary file 4 — Description of Additional Supplementary Files [file 41467_2020_15040_MOESM4_ESM.docx]

**Description of Additional Supplementary Files**

File name: Supplementary Data 1
Description: Names and abbreviations of BIA pathway genes.

File name: Supplementary Data 2
Description: Correspondence between pathway gene positioning in our assembly and in Guo assembly. Relationship between cScafs and *P. somniferum* chromosomes as defined by Guo *et al*. is shown in Supplementary Table 12
