# Supplementary material for: Gene clustering and copy number variation in alkaloid metabolic pathways of opium poppy
Source: Nat Commun. 2020 Mar 4;11:1190. doi: 10.1038/s41467-020-15040-2 (PMC7055283; doi:10.1038/s41467-020-15040-2)
Supplement: Supplementary file 2 — Peer Review [file 41467_2020_15040_MOESM2_ESM.pdf]

Editorial Note: Parts of this peer review file have been redacted as indicated to remove third-party material where no permission to publish could be obtained.

Reviewers' comments:

Reviewer #1 (Remarks to the Author):

This manuscript is a nicely executed and written report of the assembled opium poppy genome. It presents a resource that will be generally useful to the medicinal plant research community. It corroborates and compliments well a recent report in Science on the opium poppy genome. This reviewer believes that the analysis of clustering of both validated and putative biochemical pathway genes will be of particular interest to those in the natural product biosynthesis field. I cannot comment on the quality of the assembly, and thus leave that to other reviewers. All-in-all, the results presented in this manuscript are, as are all reports on plant genome sequences, most useful.

Reviewer #2 (Remarks to the Author):

This is an interesting study in that it reports on the genome assembly of poppy and through a re-sequencing approach, identified structural variants in BIA biosynthetic pathway that are correlated with differential alkaloid profiles. The study and data will certainly provide a foundation for further in vivo production of opioids and for evolutionary studies on secondary metabolism. The manuscript was clearly written for a more abbreviated journal than Nature Communications and thus, the focus of the text is on the genome, the biosynthetic pathways, and comparative analyses among the poppy cultivars via the metabolite and re-sequencing data.

The details of the genome assembly assessment are missing. Without any assessment of the quality (other than a BUSCO score which is so-so), it is hard to know if the genome is well assembled. The authors should have re-aligned Illumina reads to the genome, aligned RNA-seq reads to the genome and reported alignment metrics. The estimated flow cytometry size should be compared with the assembly size as well. How many chromosomes does poppy have? There seemed to be no validation of the HiC read generated pseudomolecules. Were known genetic markers aligned to confirm its integrity?

This lack of information on the input and output of the libraries/reads/process of the genome assembly is atypical. While this can be boring, it is best to show what was used for input/parameters and the metrics of the intermediate and final assembly.

It is surprising to me that a 2.6 Gb has a mere 42.6% transposable elements. This is highly suspicious. The maize genome (2.3 Gb) is 85% transposable elements. How robust was the repeat identification process? The annotation also seems out of line with 84,000 genes. I suspect a lot of these are transposable elements or single exon artifacts from the MAKER-P pipeline. This should be reviewed and the annotation should be improved if it is contaiminated with transposable element-related genes.

The numbers of CNV in the BIA pathway is surprising. It would have been helpful if the authors validated their CNVnator results with read depth assessments and/or allelic variant review to ensure that these are not artifacts. Could it be that the 20X libraries were not of sufficient depth to accurately call CNVs? Or are the genomes so diverged the reads did not align well?

There is a lot of emphasis on pseudogenes, which are at a rather high rate in the BIA pathway. Are these simple assembly errors? Or is the definition of pseudogene used inappropriate? Perhaps these are induced under conditions not sampled in this study? Or are at low levels and were under-sampled in this study. There is no summary of the RNAseq samples used; this would have been helpful to understand if the libraries were under-sampled especially as at some point, 454 datasets were used. Were the RNAseq studies done in replicate?

In figure 2, the cultivars should be listed in the same order in panel a and b.

More detailed figures of the BIA pathway in the reference genotype and the re-sequecned cultivars would be extremely powerful to highlight the evolution of the clusters and the divergence between cultivars.

There is no section on data availability, either raw or final datasets (genome, RNA, metabolite).

Reviewer #3 (Remarks to the Author):

In the manuscript, titled “Gene clustering and copy number variation in alkaloid metabolic pathways of opium poppy”, authors present a high-quality genome assembly of Opium poppy. Authors used this draft genome to investigate existence of gene cluster around 199 core genes possibly involved in the biosynthesis of noscapine, morphine and other alkaloids. Authors next investigated copy number variations using draft genome and Illumina-based sequencing for nine different varieties of Opium with different chemo-diversity and alkaloid profiles. Authors reported dynamic evolution of Opium poppy genome with CNVs linked to genes involved in alkaloid biosynthesis explaining the chemo-diversity within studied species of Opium poppy. Authors also observed existence and tight clustering of 58% of the biosynthetic genes leading to morphine and other alkaloid biosynthesis.
This is an important study and authors have done a nice job, experiments are done properly and enough controls are included. Authors have too many data but I find a lot of them not being properly used or rather just mentioned in the paper but no interpretation. Method described in this paper is far from complete and require extensive rewriting and need to include details of all analysis that authors have provided.
Unfortunately, two weeks earlier, Opium poppy genome was published in science (titled-“The opium poppy genome and morphine production”). I feel that this article has described genome way better, and their genome assembly is very impressive (contigs and scaffold N50 as 1.77 and 204Mb). Compared to that, this genome assembly is better at least at contig level (4+Mb) surprisingly, authors have not described scaffold N50 at all. Since this article has been published, it does make sense to compare genome assembly quality with the one that authors have achieved. Assembly approach for science paper and this one is very different, and focus is completely different as well. Therefore, I feel that this article holds the merit to go for improvement and it does provide new findings for the advancement of this field. In my opinion, despite of impressive study, this work needs extensive revision and additional analysis required for its publication. My reasons for above mentioned sentence is as follows-
1. Authors need to explain method used in this article extensively. This is really a big problem and need attention from authors. Majority of this manuscript is based on reporting gene clusters in Opium, yet what algorithm used is not mentioned. Authors have just said gene cluster algorithm used (method, line 119; supplementary method, line 85), but not mentioned about it at all. Did they created their own or used published ones such as plantiSMASH or so on? I somehow believe that authors simply looked positioning for 199 biosynthetic genes that they identified in the 11 chromosome, and looked for genes within 100kb. But not clear if this is correct assumption, if they looked up or down stream around each target genes and so on.
2. Their definition of gene cluster comes at the very end, that too in the method section and not clear at the same time. Since authors have spent so much time on explaining gene clustering, it makes sense to explain their version of definition and justification for the same. Also, it will be great if authors consider defining it somewhere at the beginning, so that readers will be able to follow entire results.
3. Method for genome assembly, particularly parameters used are completely missing. Did authors used default parameters? Authors did mention in method section (line 235) about assembler name and kmer-71, yet method described is not enough to repeat the assembly process. Did authors used corrected PacBio reads, what assembly conditions were tested? Authors mentioned about getting

scaffolding using HiC, but software used (I am assuming they used proximity software) and parameters used are completely missing. Even if final scaffolding were outsourced, it does make sense to include relevant parameters as much as possible.

4. Similarly, authors mentioned about nine different Opium species with different alkaloid profile. But no information of introduction or origin details for any of these. One can find little detail in the supplimentry datasheet (S5-S6) but that too not complete. Its not clear how the names such as 11, 40, BC, L, M, P, PS1 and so on were derived, their origin, why they picked these lines. If these lines were used before then should add the reference.

5. Although authors have listed 199 biosynthetic genes that they focused in this study associated with alkaloid biosynthetic pathways, it will be really helpful if they could provide pathway maps as well. Its not easy to follow entire study just by reading numbers. Since this study is particularly focused on secondary metabolism, including pathway maps seems essential to me.

6. Authors have provided no details about Pacbio sequencing. I mean, what they used, RSII or sequel, how many cells were used, what library preparation method (what library size, 20kb, 40 kb?), what was the N50-raw reads. All these informations are essential for readers to get a sense about genome quality, and authors have failed miserably in this aspect. This study completely lacks proper description of method and important information that provide confidence in the foundation of this study, that is genome assembly.

7. To my biggest surprised, authors have not included a table to describe genome assembly statistics. Authors did mention about number of contigs, contigs N50 in the supplementary information (supplementary method, line 8 onwards), what is scaffold N50, what are scaffold sizes, how many gaps in the genome assembly, size of gaps? This is essential information, and is completely ignored by the authors. Another aspect is HiC contact map. Authors used HiC to get scaffolding and reported 16103 contigs being placed in cScafs. But then authors also mentioned that 641 contigs although assigned could not be maped, which to me does not make sense. If these contigs are not mapped, then are they still on that cScafs or are removed? If this is a case of misassembly then how authors validated their final assembly? Authors need to explain this point either in the supplementary information or main text. Also, although authors have directly started talking about gene clusters and CNVs analysis, whole study foundation is this genome assembly and authors must mention, describe properly and in enough details about genome assembly characteristics, quality and other properties in the main text including how many contigs missed from the final assembly.

8. HiC contact map is essential to have an idea on how well authors were able to get final assembly, and it also gives idea on any possibility of mis-assembly. For me, absence of this analysis and plot makes me question the genome assembly, and I wish to have a look on contact map of HiC datasets to the final assembly. Authors could use so many free tools available to do this. Authors did mention about enrichment score, but I do not agree that this is enough information and for me, visualization is more important.

9. In supplementary information, line14-15, "….and 171 contigs could not be placed with high confidence in any of the clusters". I am assuming authors here mean cScafs. Authors have used term cluster throughout the manuscript to describe gene cluster, and therefore this discrepancy is not acceptable. Authors should check entire manuscript and resolve it.

10. What is expected genome size? This is most fundamental information that is missing here. Either kmer based or flow-cytometer based or atleast use a reference to say estimated genome size for the plant that authors have sequenced.

11. I believe that supplementary method, line 101, "The results suggested…" it should be 199 and not 198. Authors should verify and correct this.

12. Authors performed expression analysis for several tissues of Opium poppy at different developmental stages but not explained different aspects of it in detail. This part is disappointing to me as several information from this analysis will be useful. For example, are genes that are part of a cluster are co-expressed, and how much percentage of clustered genes that authors identified are co-expressed. Further, does expression and metabolome datasets are following similar trend? Is there any uncharacterized gene that are co-expressed with BIAs and are clustered? To my surprise, authors have quality expression and metabolome datasets (I am fine with single replicate transcription dataset as long as interpretation is just trend and expression is verified through RT-PCR), yet they have not

put any effort to analyze and interpret to further strengthen their research outcome and conclusion. This is another part that I feel authors need to do lots of work. In my opinion, just representing expression in form of heat map without interpretation is not enough.

13. This is minor point but will like to ask authors about metabolome study. Authors in the method section describe that they used 100mg fresh weight of tissue for each replicate for metabolite extraction. Yet they have described quantification of metabolites in the unit of nmol/g (dry weight). Does this mean that authors then freeze dried these tissues before metabolite extraction or 100mg is actually dried sample weight and not fresh weight? Based on my understanding, different tissues will have different water content and hence freeze dried tissue is most appropriate to achieve a common baseline for comparative metabolome analysis, especially when one focuses on different tissue types of same plant for differential metabolome analysis. For identification of new metabolites, of course this is not essential. I am wondering if method described was not correct or how authors will explain figure and method (Extended data figure 4, 5).

14. It will be wonderful if authors could propose list of genes that are clusters and are promising candidates to be involved in BIAs biosynthesis. Authors should explain in detail and more rational as why they think that way.

Broad overview of the revisions involved this resubmission

Upon comparing our previous draft assembly with that of the Guo et al. assembly, we found that mapping rates with our genome were ~10% lower, indicating that our assembly was lower in quality. This is unsurprising, given the higher depth of long-read sequencing and bacterial artificial chromosomes used by Guo et al. However, we noted that the Guo et al. assembly used a very low-powered linkage mapping approach to scaffold their contigs, whereas we had used a Hi-C approach to scaffolding that should yield higher power. We decided to proceed by fragmenting the Guo et al. assembly where contigs had been joined by linkage mapping, and re-scaffolding the contigs using our Hi-C approach.

This approach yielded a substantially improved assembly: whereas 75% of our Illumina reads mapped to the Guo et al. assembly, this improved to 85% with our re-scaffolded assembly. Our assembly also scaffolded contigs containing 39 BIA genes that had previously been unmapped. Taken together, we are therefore confident that this assembly represents the best currently available draft genome for this species.

Using this new assembly, we conducted an extensive re-analysis of most of our main work: we extended the study of clustering of benzylisoquinoline alkaloid (BIA) genes on the chromosome-scale scaffolds, mapped our RNAseq reads to the new assembly and re-analysed patterns of expression, and re-analysed the study of enrichment of transposable elements in the BIA gene clusters. We also added new analyses on gene co-expression within vs. among the BIA pathway gene clusters and studied covariation between gene expression and alkaloid production.

Responses to reviewer points (in blue)

Reviewers' comments:

Reviewer #1 (Remarks to the Author):

This manuscript is a nicely executed and written report of the assembled opium poppy genome. It presents a resource that will be generally useful to the medicinal plant research community. It corroborates and compliments well a recent report in Science on the opium poppy genome. This reviewer believes that the analysis of clustering of both validated and putative biochemical pathway genes will be of particular interest to those in the natural product biosynthesis field. I cannot comment on the quality of the assembly, and thus leave that to other reviewers. All-in-all, the results presented in this manuscript are, as are all reports on plant genome sequences, most useful.

Thank you for the positive feedback.

Reviewer #2 (Remarks to the Author):

This is an interesting study in that it reports on the genome assembly of poppy and through a re-sequencing approach, identified structural variants in BIA biosynthetic pathway that are correlated with differential alkaloid profiles. The study and data will certainly provide a foundation for further in vivo production of opioids and for evolutionary studies on secondary metabolism. The manuscript was clearly written for a more abbreviated journal than Nature Communications and thus, the focus of the text is on the genome, the biosynthetic pathways, and comparative analyses among the poppy cultivars via the metabolite and re-sequencing data.

The details of the genome assembly assessment are missing. Without any assessment of the quality (other than a BUSCO score which is so-so), it is hard to know if the genome is well assembled. The authors should have re-aligned Illumina reads to the genome, aligned RNA-seq reads to the genome and reported alignment metrics. The estimated flow cytometry size should be compared with the assembly size as well. How many chromosomes does poppy have? There seemed to be no validation of the HiC read generated pseudomolecules. Were known genetic markers aligned to confirm its integrity?

This lack of information on the input and output of the libraries/reads/process of the genome assembly is atypical. While this can be boring, it is best to show what was used for input/parameters and the metrics of the intermediate and final assembly.

We agree and regret that this information was missing from the previous manuscript, which was partly omitted for reasons of length. We have made sure to extend the discussion of these metrics where relevant to the new assembly.

There was broad correspondence between the scaffolding that we conducted using Hi-C and the linkage mapping done by Guo et al. (2018), as is now shown in the Supplementary materials Figure S4, and we have compared the read mapping percentages between these two genomes (75% vs. 85% for Guo et al. vs. our re-scaffolded assembly, Table S1), as well as reporting a range of quality metrics in the main text.

It is surprising to me that a 2.6 Gb has a mere 42.6% transposable elements. This is highly suspicious. The maize genome (2.3 Gb) is 85% transposable elements. How robust was the repeat identification process?

We thank the reviewer for pointing out this issue, as we discovered a bug in our pipeline for annotating the transposable elements, and we agree that 42.6% was unreasonably low. As we are using the contigs from the Guo et al. (2018) assembly, the TE content is not affected by the Hi-C scaffolding we conducted, and the results from their paper on TE content still apply. However, we note that we have expanded the study of TEs here, examining the age of various elements (Figure S16) and the patterns of TE enrichment within the BIA clusters (Table S10) and their potential involvement in tandem duplications (Figure S5).

The annotation also seems out of line with 84,000 genes. I suspect a lot of these are transposable elements or single exon artifacts from the MAKER-P pipeline. This should be reviewed and the annotation should be improved if it is contaminated with transposable element-related genes.

This no longer applies to the new assembly.

The numbers of CNV in the BIA pathway is surprising. It would have been helpful if the authors validated their CNVnator results with read depth assessments and/or allelic variant review to ensure that these are not artifacts. Could it be that the 20X libraries were not of sufficient depth to accurately call CNVs? Or are the genomes so diverged the reads did not align well?

We conducted some spot-checking of the results from CNVnator and it appears to be working properly as designed. We note that this is a very commonly used approach and that its algorithms are undoubtedly superior to any ad-hoc analyses using depth and/or allelic variants that we might have coded ourselves.

There is a lot of emphasis on pseudogenes, which are at a rather high rate in the BIA pathway. Are these simple assembly errors? Or is the definition of pseudogene used inappropriate? Perhaps these are induced under conditions not sampled in this study? Or are at low levels and were under-sampled in this study. There is no summary of the RNAseq samples used; this would have been helpful to understand if the libraries were under-sampled especially as at some point, 454 datasets were used. Were the RNAseq studies done in replicate?

Generally, premature stop codons or frameshift mutations provide strong evidence of a gene being a pseudogene, while lack of detectable expression provides only weak evidence, as it may be that we simply didn’t sample the tissue/timepoint/condition that is important for gene expression. We have de-emphasized the discussion of pseudo genes, as it wasn’t central to our analysis here.

In figure 2, the cultivars should be listed in the same order in panel a and b.

This has been fixed in the modified version.

More detailed figures of the BIA pathway in the reference genotype and the re-sequecned cultivars would be extremely powerful to highlight the evolution of the clusters and the divergence between cultivars.

We have added a figure showing the BIA pathway to the main materials (Figure 1) and a more detailed figure in the supplementary materials (Figure S1).

There is no section on data availability, either raw or final datasets (genome, RNA, metabolite).

This has been added to the end of the main materials.

Reviewer #3 (Remarks to the Author):

In the manuscript, titled "Gene clustering and copy number variation in alkaloid metabolic pathways of opium poppy", authors present a high-quality genome assembly of Opium poppy. Authors used this draft genome to investigate existence of gene cluster around 199 core genes possibly involved in the biosynthesis of noscapine, morphine and other alkaloids. Authors next investigated copy number variations using draft genome and Illumina-based sequencing for nine different varieties of Opium with different chemo-diversity and alkaloid profiles. Authors reported dynamic evolution of Opium poppy genome with CNVs linked to genes involved in alkaloid biosynthesis explaining the chemo-diversity within studied species of Opium poppy. Authors also observed existence and tight clustering of 58% of the biosynthetic genes leading to morphine and other alkaloid biosynthesis.
This is an important study and authors have done a nice job, experiments are done properly and enough controls are included. Authors have too many data but I find a lot of them not being properly used or rather just mentioned in the paper but no interpretation. Method described in this paper is far from complete and require extensive rewriting and need to include details of all analysis that authors have provided.

We agree that the main text was far too brief for a comprehensive treatment of this topic, and it has been expanded in this now longer-format article. We have tried to expand and clarify the methods throughout the paper as well.

Unfortunately, two weeks earlier, Opium poppy genome was published in science (titled- "The opium poppy genome and morphine production"). I feel that this article has described genome way better, and their genome assembly is very impressive (contigs and scaffold N50 as 1.77 and 204Mb). Compared to that, this genome assembly is better at least at contig

level (4+Mb) surprisingly, authors have not described scaffold N50 at all. Since this article has been published, it does make sense to compare genome assembly quality with the one that authors have achieved. Assembly approach for science paper and this one is very different, and focus is completely different as well. Therefore, I feel that this article holds the merit to go for improvement and it does provide new findings for the advancement of this field. In my opinion, despite of impressive study, this work needs extensive revision and additional analysis required for its publication. My reasons for above mentioned sentence is as follows-

When we compared our previous hybrid assembly to the Guo et al. assembly, we found that the rates of read mapping were ~10% lower in our genome than in their genome, which means that our assembly was of lower quality. Rather than push forwards with an inferior draft assembly, we opted to improve the best existing resource, using our Hi-C scaffolding to improve the scaffolding they had previously conducted on the linkage mapping. We have reported these assembly metrics more comprehensively now and are confident that our new assembly represents the best available draft genome.

1. Authors need to explain method used in this article extensively. This is really a big problem and need attention from authors. Majority of this manuscript is based on reporting gene clusters in Opium, yet what algorithm used is not mentioned. Authors have just said gene cluster algorithm used (method, line 119; supplementary method, line 85), but not mentioned about it at all. Did they created their own or used published ones such as plantiSMASH or so on? I somehow believe that authors simply looked positioning for 199 biosynthetic genes that they identified in the 11 chromosome, and looked for genes within 100kb. But not clear if this is correct assumption, if they looked up or down stream around each target genes and so on.

Yes, we had previously simply looked 100kb upstream and downstream of each BIA candidate gene to see if there was at least one other candidate gene within this window. We have now extended this approach to explore a range of clustering sizes, and implemented two different test of significance (a less conservative “global” test and a more conservative “incremental” test).

We elected to use this approach because it focuses explicitly on the importance of spatial positioning of known genes on chromosomes, without using any information on gene expression or enzyme co-expression. We did this because we did not want to risk excluding interesting candidates because of lack of information about their enzyme function or lack of observed co-expression. While it is a excellent algorithm for some applications, the strengths of PlantiSmash are not well-suited to our purposes, because it is based on using existing databases of enzymes known to be involved in biosynthesis and gene co-expression

data to detect clusters. We felt that this approach would be overly restrictive, as it would exclude genes from poppy that are not yet part of these databases. Also, because gene co-expression is only one of several potential drivers of cluster formation, we did not want to limit our discovery of clusters to only those that included genes with evidence for co-expression. Finally, if we had used gene co-expression to identify clusters, we could not study patterns of expression within vs. among clusters without such patterns being confounded by the data used in the clustering algorithm. As such, we felt that a simple and transparent heuristic for cluster identification was more appropriate.

2. Their definition of gene cluster comes at the very end, that too in the method section and not clear at the same time. Since authors have spent so much time on explaining gene clustering, it makes sense to explain their version of definition and justification for the same. Also, it will be great if authors consider defining it somewhere at the beginning, so that readers will be able to follow entire results.

This is a good point, we can see that it wasn't particularly clear in the manuscript as previously written. We have clarified this where the analysis of clustering is first introduced.

3. Method for genome assembly, particularly parameters used are completely missing. Did authors used default parameters? Authors did mention in method section (line 235) about assembler name and kmer-71, yet method described is not enough to repeat the assembly process. Did authors used corrected PacBio reads, what assembly conditions were tested? Authors mentioned about getting scaffolding using HiC, but software used (I am assuming they used proximity software) and parameters used are completely missing. Even if final scaffolding were outsourced, it does make sense to include relevant parameters as much as possible.

This is no longer relevant, as we opted to not use the previous assembly.

4. Similarly, authors mentioned about nine different Opium species with different alkaloid profile. But no information of introduction or origin details for any of these. One can find little detail in the supplimentry datasheet (S5-S6) but that too not complete. Its not clear how the names such as 11, 40, BC, L, M, P, PS1 and so on were derived, their origin, why they picked these lines. If these lines were used before then should add the reference.

We have added information to the methods about the origin of these strains. Unfortunately there is not a lot of information available about the origins of some of these strains, but we have added references and information where possible.

5. Although authors have listed 199 biosynthetic genes that they focused in this study associated with alkaloid biosynthetic pathways, it will be really helpful if they could provide pathway maps as well. It's not easy to follow entire study just by reading numbers. Since this study is particularly focused on secondary metabolism, including pathway maps seems essential to me.

We have added a table to the supplementary materials to clarify the gene name usage (Table S2) as well as a pathway diagram showing the genes in the main pathways (Figure S1).

6. Authors have provided no details about Pacbio sequencing. I mean, what they used, RSII or sequel, how many cells were used, what library preparation method (what library size, 20kb, 40 kb?), what was the N50-raw reads. All these informations are essential for readers to get a sense about genome quality, and authors have failed miserably in this aspect. This study completely lacks proper description of method and important information that provide confidence in the foundation of this study, that is genome assembly.

This is no longer relevant, as we opted to not use the previous assembly.

7. To my biggest surprised, authors have not included a table to describe genome assembly statistics. Authors did mention about number of contigs, contigs N50 in the supplementary information (supplementary method, line 8 onwards), what is scaffold N50, what are scaffold sizes, how many gaps in the genome assembly, size of gaps? This is essential information, and is completely ignored by the authors. Another aspect is HiC contact map. Authors used HiC to get scaffolding and reported 16103 contigs being placed in cScafs. But then authors also mentioned that 641 contigs although assigned could not be maped, which to me does not make sense. If these contigs are not mapped, then are they still on that cScafs or are removed? If this is a case of misassembly then how authors validated their final assembly? Authors need to explain this point either in the supplementary information or main text. Also, although authors have directly started talking about gene clusters and CNVs analysis, whole study foundation is this genome assembly and authors must mention, describe properly and in enough details about genome assembly characteristics, quality and other properties in the main text including how many contigs missed from the final assembly.

This is no longer relevant, as we opted to not use the previous assembly, but we have taken care to be more explicit about the methods and assembly details in the current manuscript.

8. HiC contact map is essential to have an idea on how well authors were able to get final assembly, and it also gives idea on any possibility of mis-assembly. For me, absence of this analysis and plot makes me question the genome assembly, and I wish to have a look on contact map of HiC datasets to the final assembly. Authors could use so many free tools available to do this. Authors did mention about enrichment score, but I do not agree that this is enough information and for me, visualization is more important.

This has been added as a new figure in the supplementary materials (Figure S2).

9. In supplementary information, line14-15, "….and 171 contigs could not be placed with high confidence in any of the clusters". I am assuming authors here mean cScafs. Authors have used term cluster throughout the manuscript to describe gene cluster, and therefore this discrepancy is not acceptable. Authors should check entire manuscript and resolve it.

We apologize for this oversight, this should have been "cScaf", however this is no longer relevant in the revised draft.

10. What is expected genome size? This is most fundamental information that is missing here. Either kmer based or flow-cytometer based or atleast use a reference to say estimated genome size for the plant that authors have sequenced.

We note that a previous paper used flow cytometry to get an estimation of genome size of 3.16 Gbp (Kyrylenko et al. 2005; Nuclear genome size and karyotype analysis in Papaver for BAC library construction, DOI: 10.7124/bc.0006E5), and we have added the k-mer based method of inference for the 9 re-sequenced genomes, which we report on Table S8 which have a mean value of 3.02 Gbp, which is reasonably close to the flow cytometry estimate. We do not discuss this aspect extensively, as we have not altered the contig assembly produced by Guo et al. 2018.

11. I believe that supplementary method, line 101, "The results suggested…" it should be 199 and not 198. Authors should verify and correct this.

This is no longer relevant in the revised manuscript.

12. Authors performed expression analysis for several tissues of Opium poppy at different developmental stages but not explained different aspects of it in detail. This part is disappointing to me as several information from this analysis will be useful. For example, are genes that are part of a cluster are co-expressed, and how much percentage of clustered genes that authors identified are co-expressed. Further, does expression and metabolome datasets are following similar trend? Is there any uncharacterized gene that are co-expressed with BIAs and are clustered? To my surprise, authors have quality expression and

metabolome datasets (I am fine with single replicate transcription dataset as long as interpretation is just trend and expression is verified through RT-PCR), yet they have not put any effort to analyze and interpret to further strengthen their research outcome and conclusion. This is another part that I feel authors need to do lots of work. In my opinion, just
representing expression in form of heat map without interpretation is not enough.

This is an excellent point. We had originally limited our analysis of the co-expression aspects of this data because of limited space in the short-format article we had prepared. We have now expanded this extensively, adding novel analyses on co-expression, comparing patterns of co-expression within clusters to patterns of co-expression both among clusters and among randomly-chosen pairs of neighbouring genes not found in clusters.

13. This is minor point but will like to ask authors about metabolome study. Authors in the method section describe that they used 100mg fresh weight of tissue for each replicate for metabolite extraction. Yet they have described quantification of metabolites in the unit of nmol/g (dry weight). Does this mean that authors then freeze dried these tissues before metabolite extraction or 100mg is actually dried sample weight and not fresh weight? Based on my understanding, different tissues will have different water content and hence freeze dried tissue is most appropriate to achieve a common baseline for comparative metabolome analysis, especially when one focuses on different tissue types of same plant for differential metabolome analysis. For identification of new metabolites, of course this is not essential. I am wondering if method described was not correct or how authors will explain figure and method (Extended data figure 4, 5).

We agree that dry weight (not fresh weight) needs to be used to normalize metabolome data, and this was the case for our study. We have changed the section in the supplementary materials to make this more clear ("Metabolite Analysis").

14. It will be wonderful if authors could propose list of genes that are clusters and are promising candidates to be involved in BIAs biosynthesis. Authors should explain in detail and more rational as why they think that way.

This is a great point, and we have added extensive discussion of this aspect in the revised manuscript, and have added a list of all 109 BIA genes and their locations in the genome (Table S3).

Reviewers' comments:

Reviewer #2 (Remarks to the Author):

This manuscript is greatly improved from the previous version. I have some specific comments on the manuscript that should be addressed.

Line 119: Just using mate pair reads to assess quality is insufficient to make any statements on the quality of an assembly, especially since the authors are comparing 2 scaffolding approaches for the same underlying contig set. Concordance with other datasets such as genetic maps, synteny, etc should be pursued to establish how well this new assembly represents known information about the poppy genome.

Line 366: It is still surprising that only 68% of a 3 Gb genome is transposable elements. Is the rest genes? And intergenic space?

Line 405-410: No evidence was provided for deletions. This could be an insertion and then IBD regions as it is clear the origins of these poppy accessions is unknown.

Line 781: Where will the genome assembly be released? Dryad?

Table 1 can be deleted completely. It is difficult to read and not essential to the manuscript as the information is presented elsewhere.

Figure 3 a-c. The figure is so small that it is impossible to read what is in the figure. Color choices could be improved for contrast.

Figure 4 all panels: The figure is so small it cannot be read.

Figure S2: The contact map is a bit messy justifying the need for additional QC of the scaffolded assembly.

All supplemental figures would benefit from expanded figure legends.

Addition of a table of the metrics of the final genome assembly with number of chromosomes, number of scaffolds, numbers of contigs, N50s, number of genes, etc would greatly help the reader interpret the quality of the genome.

Reviewer #3 (Remarks to the Author):

This article is a revision of the previously submitted article, titled "Gene clustering and copy number variation in alkaloid metabolic pathways of Opium poppy", where authors described Poppy genome, explained wide-spread gene clusters and copy number variation analysis using multiple species with varying levels of alkaloids production. Compared to the previously submitted article, where they used their own dataset to assemble the genome, in this revised manuscript, authors used recently published Poppy genome, used HiC datasets to improve scaffolding of the genome and then performed subsequent analysis on the same line as the previous version. In response to my concerns, authors were successful to address most of them and now results do look more useful for downstream validation and advancing the present knowledge. But I think that it is difficult to read this article based

on the first submission as the main foundation for this study, plant genome, is changed. I do believe that some of the interpretations from this study is useful and probably important, but I have many concerns as mentioned below (My only major concern is final genome assembly, rest analysis seems good to me).
1. I do not understand why Authors choose to discard their own assembly. I know that authors argued that Guo et al Poppy genome has used high depth of Pacbio coverage (65x compared to their 10x coverage in the first version), I did notice that authors claimed their genome assembly contig N50 as 4Mb plus while Gua et al assembly N50 is 1.7Mb. Scaffolding is just going to arrange the contigs to pseudo-molecule but it does not add any contiguity. So, I wonder if the authors decided to improve the genome assembly, why did not they used their original one, and used HiC to significantly improve genome quality.
2. Another point is reusing assembly datasets from Guo et al article and combining with your dataset to improve genome quality. I do not see why authors should abandon their analysis completely. Did the authors tried and see no advantage?
3. Using a published genome, and adding HiC based scaffolding to include previously unassigned contigs/scaffold to the pseudomolecule is a good step, but I am not sure if that qualifies a study to be published in a journal like Nature communication where the expectation from studies is high. I know that the study is not just genome improvement, but thats the foundation and almost 50% of this study.
4. Authors compared Poppy genome published by Guo et al and their newly scaffolded genome. As authors mentioned, although they observed a lot of similarities, in almost all cases, authors observed inversion along chromosomes (Fig S4) and in many cases genes/contigs/scaffolds assigned to different pseudo molecule than the published report. Authors argued that linked libraries and HiC are a different method to perform scaffolding, and hence this difference. But the question remains is which assembly should be trusted? How authors can say that assigning genes/contigs/scaffolds to a new pseudomolecule is correct? Based on this study, it is not possible to ascertain. If authors could show physical evidence such as FISH analysis which could prove that indeed that’s the case, then that’s the ideal scenario and real improvement. Such analysis will then improve our knowledge of Poppy genome, and as a researcher, I will be confident to use it. At this stage, I do not know how and why I should trust this genome over published Guo et al Poppy genome. According to my opinion, HiC based scaffolding is not enough improvement, but adding physical evidence is essential to make this data more useful and informative for the research community.
5. All analysis except metabolome and copy number variance analysis could be done with nearly the same conclusion using Guo et al published genome. In this study, indeed, authors have done an amazing job to explain clusters and have drawn nice interpretation, but except few instances where authors could assign genes to pseudomolecule, I can not see anything new that can not be done using old assembly. Further, authors mentioned about strict and loose criteria to define a gene cluster, but that sounds too unrealistic, especially expecting gene clusters over 1Mb. In best of my knowledge, I do not know any reported clusters for a plant till date within this long-range genomic space.
6. Also, the authors used their HiC dataset to scaffold Guo et al results, but are these two species are clonal. If not, then the difference that authors reported (Fig S4) is because of that. If yes, then the whole strategy is wrong as using HiC data for non-clonal assembly could result in wrong final assembly

Reviewers' comments:

Reviewer #2 (Remarks to the Author):

This manuscript is greatly improved from the previous version. I have some specific comments on the manuscript that should be addressed.

Line 119: Just using mate pair reads to assess quality is insufficient to make any statements on the quality of an assembly, especially since the authors are comparing 2 scaffolding approaches for the same underlying contig set. Concordance with other datasets such as genetic maps, synteny, etc should be pursued to establish how well this new assembly represents known information about the poppy genome.

We have searched extensively for useful datasets that could help us evaluate the fit of our scaffolding approach. Unfortunately, very little work has been done on linkage mapping in this species nor any closely related species. We contacted the authors of one linkage map we did find published, by Straka and Nothnagel (2008; Journal of Herbs, Spices, and Medicinal Plants; https://doi.org/10.1300/J044v09n02_a), but unfortunately their approach used random primers and electrophoresis for genotyping, which does not allow for any simple approach to compare their map to our genome. We have added further verifications of our scaffolding using other sources of independent sequence-based evidence, as described in the overview above.

Line 366: It is still surprising that only 68% of a 3 Gb genome is transposable elements. Is the rest genes? And intergenic space?

We agree that this does seem low, but we note that this percentage just refers to the parts of the genome that can clearly be identified as particular TE's. It is likely that a substantial part of the remaining 32% is derived from very old TE insertions that have evolved sufficiently to no longer clearly bear a signature of their origin. We have clarified this in the text at line 391-394.

Line 405-410: No evidence was provided for deletions. This could be an insertion and then IBD regions as it is clear the origins of these poppy accessions is unknown.

While we agree that it is often not possible to ascertain whether a given difference in sequence content is an insertion or deletion, especially with small indels, in these cases entire genes are missing with no detectable sequence from any copy of T6ODM or from the noscapine genes in the accessions being discussed. If these polymorphisms were due to insertions, they would have to come from somewhere, and we would have detected reads that would still map to the source of the duplication. Thus, it is more clear to call these deletions and we prefer to leave the text as it currently stands here.

Line 781: Where will the genome assembly be released? Dryad?

Yes, we will deposit this genome assembly on Dryad and include the accession number in the paper if it is eventually accepted for publication.

Table 1 can be deleted completely. It is difficult to read and not essential to the manuscript as the information is presented elsewhere.

Thank you for catching this, it should not have been included as it duplicates table S4, and has been removed.

Figure 3 a-c. The figure is so small that it is impossible to read what is in the figure. Color choices could be improved for contrast.
Good point, we are sorry for the poor presentation with these figures. We have increased the font size of these figures (now Figure 3), separated A-C from panel D, which is now Figure 4. We have also changed the grey dots in panel B to "hollow" circles, as this provides better contrast.

Figure 4 all panels: The figure is so small it cannot be read.

We have divided the old Figure 4 into two separate figures (Figure 5 & 6) and modified the text sizes to improve readability.

Figure S2: The contact map is a bit messy justifying the need for additional QC of the scaffolded assembly.

We contacted Shawn Sullivan, who works at Phase Genomics and conducted the Hi-C library prep and sequencing for us. Regarding the quality of the Hi-C contact map, Dr. Sullivan stated:

"*Qualitatively, I'd respectfully disagree with the reviewer's comment on the appearance of the heatmap. I've seen thousands of these and this is a pretty nice, clean one. The main diagonal especially is clean, which is a good sign. If the reviewer's comments are more about the off-diagonal signal, there are two things that might help explain what's going on. First, there are secondary diagonal lines in several of the off-diagonal regions. These are things we often see between homologous chromosome scaffolds that have been well assembled and scaffolded. They usually arise from Hi-C reads mapping to homologous, syntenic sequences. Second, there are some small enhancements in the inter-chromosomal Hi-C signal in the corners of some of the off-diagonal boxes. That is a phenomenon we often see that is associated with genuine biology, having to do with the way the chromatin is organized in the cell. When present on its own, the telomere bouquet architecture can frequently exhibit this signal, and when seen with centromeric Hi-C signal (which appears as a grid-like punctate pattern in the center of the off-diagonal boxes), the Rabl configuration frequently produces that signal.*"

We do agree that it is important that we perform additional assessment about the scaffolding accuracy, so we conducted further bioinformatic checks of our scaffolding joins using long-read PacBio data from two different individuals (HN1 and PS7), as described above. This sequence data was previously used in our original assembly but was not used in the most recent Hi-C assembly that we conducted using the Guo et al. contigs. Thus, these reads constitute an independent source of data about scaffolding that we can use to validate our Hi-C joins, compared to the linkage-map joins made in the Guo et al. genome.

All supplemental figures would benefit from expanded figure legends.

Thank you for pointing this out, we have extended the supplemental figure legends wherever they were lacking information.

Addition of a table of the metrics of the final genome assembly with number of chromosomes, number of scaffolds, numbers of contigs, N50s, number of genes, etc would greatly help the reader interpret the quality of the genome.

This information has now been added to a new Table S1 in the supplementary materials.

Reviewer #3 (Remarks to the Author):

This article is a revision of the previously submitted article, titled "Gene clustering and copy number variation in alkaloid metabolic pathways of Opium poppy", where authors described Poppy genome, explained wide-spread gene clusters and copy number variation analysis using multiple species with varying levels of alkaloids production. Compared to the previously submitted article, where they used their own dataset to assemble the genome, in this revised manuscript, authors used recently published Poppy genome, used HiC datasets to improve scaffolding of the genome and then performed subsequent analysis on the same line as the previous version. In response to my concerns, authors were successful to address most of them and now results do look more useful for downstream validation and advancing the present knowledge. But I think that it is difficult to read this article based on the first submission as the main foundation for this study, plant genome, is changed. I do believe that
some of the interpretations from this study is useful and probably important, but I have many concerns as mentioned below (My only major concern is final genome assembly, rest analysis seems good to me).

We are glad that the remaining analysis looks good, as it is this aspect of the paper that we feel is the strongest and most important contribution. That said, we also want to make sure that the improvements in genome assembly that are made by our Hi-C approach are properly assessed, so we have extended our quality control assessment, as described in the Overview above and in detail below.

1. I do not understand why Authors choose to discard their own assembly. I know that authors argued that Guo et al Poppy genome has used high depth of Pacbio coverage (65x compared to their 10x coverage in the first version), I did notice that authors claimed their genome assembly contig N50 as 4Mb plus while Gua et al assembly N50 is 1.7Mb. Scaffolding is just going to arrange the contigs to pseudo-molecule but it does not add any contiguity. So, I wonder if the authors decided to improve the genome assembly, why did not they used their original one, and used HiC to significantly improve genome quality.

Unfortunately, the 4Mbp N50 size that we had reported in the original assembly was a mistake that was off by an order of magnitude. The N50 of this original assembly was actually ~400kbp, not ~4Mbp. The PI (Dr. Yeaman) takes responsibility for not noticing this mistake. Given that we also found a lower % of reads mapping to our original assembly than the Guo et al. assembly, we did not feel that it made sense to continue with our inferior assembly. It was for this reason that we decided to start from scratch, redoing all of our analyses using the best available draft (i.e. the Guo et al. assembly), and improving this draft as much as we could with our Hi-C reads. We were most interested in what these genomes reveal about the clustering of BIA genes and about the variation in CNV's, gene expression, and metabolic profiles, and were therefore most motivated to use whatever genome assembly was "state-of-the-art".

Any mistake as important as this will inevitably bring legitimate questions from reviewers about quality control. The PI (Dr. Yeaman) very much regrets how this mistake could have arisen, and enlisted the help of one of his senior lab members who was not part of the original study (Dr. Qiushi Li) to spearhead the extension of the Hi-C scaffolding and re-analysis of all the data, as well as two additional lab members (Dr. Pooja Singh and Sonja Dunemann), who helped with additional analysis and quality checking.

Despite this small but very important error in N50 reporting, we were happy to see that most of the main results that we reported in our original paper (clustering of BIA pathway genes, CNV's that were linked to noscapine and thebaine production) were not sensitive to which assembly we used.

2. Another point is reusing assembly datasets from Guo et al article and combining with your dataset to improve genome quality. I do not see why authors should abandon their analysis completely. Did the authors tried and see no advantage?

While we could have used the PacBio data from our original assembly to also further scaffold the contigs from the Guo et al., we did some preliminary testing and did not find that it yielded much improvement. As indicated by the coverage of our PacBio data, now reported in Table S3, very few of the gaps were covered by PacBio reads and so this wouldn't have improved things much. We suspect that any of the regions that were not assembled in the Guo et al. genome were likely highly repetitive and therefore resistant to improvement this way. Given that our reads would only constitute an increase of total PacBio read depth from 60x to 70x (combined reads from Guo et al. and our sequencing), we decided to avoid the complications involved in any further scaffolding using the PacBio data, and instead used this data as a validation step only.

3. Using a published genome, and adding HiC based scaffolding to include previously unassigned contigs/scaffold to the pseudomolecule is a good step, but I am not sure if that qualifies a study to be published in a journal like Nature communication where the expectation from studies is high. I know that the study is not just genome improvement, but thats the foundation and almost 50% of this study.

We respectfully disagree here, and note that our study goes well beyond a genome assembly, with extensive study of clustering of BIA pathway genes and an analysis linking copy number variation, gene expression, and metabolic profiling, which has never yet been done in opium poppy, and seldom if ever done in plants for such a high complexity secondary metabolic pathway. The improved genome allows us to conduct a more rigorous assessment of clustering than was possible using the Guo et al. genome, as we are now able to place all pathway genes on scaffolds, including 39 genes/paralogs that were previously unplaced. While this can appear from some angles as an "incremental" improvement in the genome, we feel that the rest of the study goes well beyond what can be learned from merely assembling a genome.

4. Authors compared Poppy genome published by Guo et al and their newly scaffolded genome. As authors mentioned, although they observed a lot of similarities, in almost all cases, authors observed inversion along chromosomes (Fig S4) and in many cases genes/contigs/scaffolds assigned to different pseudo molecule than the published report. Authors argued that linked libraries and HiC are a different method to perform scaffolding, and hence this difference. But the question remains is which assembly should be trusted? How authors can say that assigning genes/contigs/scaffolds to a new pseudomolecule is correct? Based on this study, it is not possible to ascertain. If authors could show physical evidence such as FISH analysis which could prove that indeed that's the case, then that's the ideal scenario and real improvement. Such analysis will then improve our knowledge of Poppy genome, and as a researcher, I will be confident to use it. At this stage, I do not know how and why I should trust this genome over published Guo et al Poppy genome. According to my opinion, HiC based scaffolding is not enough improvement, but adding physical evidence is essential to make this data more useful and informative for the research community.

We agree that every genome assembly is a draft, and we readily admit there will be many errors in our assembly as well. We consulted extensively with colleagues about the potential of using FISH or other newer approaches such Oligopaints, but decided that such approaches would take too long to develop to be feasible, as we haven't yet worked with these protocols ourselves. Preliminary investigation suggested this would take at least 6 months to get working and would not yield a high degree of confidence – for example, it would be difficult to assess many of the putative inversions that the reviewer notes using FISH.

Nonetheless, the reviewer makes an excellent point that we should have as much confidence in these results as possible, and establish more rigorously whether the scaffolding joins that we made are accurate. As described above in the Overview, we opted to use our PacBio reads to assess the accuracy of scaffolding of our Hi-C genome as compared to the original Guo et al. assembly.

5. All analysis except metabolome and copy number variance analysis could be done with nearly the same conclusion using Guo et al published genome. In this study, indeed, authors have done an amazing job to explain clusters and have drawn nice interpretation, but except few instances where authors could assign genes to pseudomolecule, I can not see anything new that can not be done using old assembly. Further, authors mentioned about strict and loose criteria to define a gene cluster, but that sounds too unrealistic, especially expecting gene clusters over 1Mb. In best

of my knowledge, I do not know any reported clusters for a plant till date within this long-range genomic space.

We note that 39 genes/paralogs/pseudogenes were not placed on chromosome-scale scaffolds in the Guo et al. assembly, representing 36% of all of the BIA pathway genes that we included in our analysis. By contrast, all 109 mapped BIA pathway genes are included in chromosome-scale scaffolds in our assembly. Respectfully, it simply is not true that this analysis could have been conducted to such a high standard using the Guo et al. genome, with 36% missing data.

We also note that searching for gene clusters over large distances than 1Mb is very justified given one of the putative explanations for cluster evolution: that selection favours a reduction in recombination among co-adapted loci, which can favour the fixation of rearrangements that build clusters and thereby reduce recombination (Yeaman 2013; PNAS). By this explanation, substantial benefits due to linkage among genes can accrue when recombination rates (r) are reduced below the strength of selection on the loci involved (s). Thus, if selection of ~10% is acting on two genes that are unlinked, a rearrangement that reduces their rate of recombination below ~10% (i.e. 10 cM) could be favoured. Given the large genome size in poppy (chromosomes are >150Mbp), meaningful clustering by this mechanism could occur over several Mbp. We had previously discussed this on line 442, and we agree with the reviewer that this is a much larger clustering distance than has been reported previously. Both of our “global” and “incremental” analyses showed that there was more clustering at this scale than would be expected by chance, which we feel provides strong evidence of its importance.

6. Also, the authors used their HiC dataset to scaffold Guo et al results, but are these two species are clonal. If not, then the difference that authors reported (Fig S4) is because of that. If yes, then the whole strategy is wrong as using HiC data for non-clonal assembly could result in wrong final assembly.

This species/these accessions are not clonal, so we will address the reviewer’s concern that the differences between the assemblies shown in Figure S4 could arise from differences between the accession we used for Hi-C scaffolding compared to the accession used by Guo et al. We agree that some of the differences in the two assemblies shown in Figure S4 could be true differences in the genomes of these accessions, but it is also possible that they are bioinformatic artifacts/errors. Some combination of both explanations is likely. As described above, we found much better mapping of PacBio reads to our Hi-C genome (~25%, compared to 4.7% of joins in the Guo et al. assembly), and more importantly, very little difference between the number of joins with reads that mapped in the PS7 and HN1 cultivars (199 vs. 185 joins mapped, with 146 of these having coverage in both cultivars, out of 768 breaks; Table S3. This suggests that the differences in cultivars used in the two studies does not introduce many of the differences between assemblies, and that our assembly likely represents a much more accurate picture of the genome. We expanded our assessment of the scaffolding accuracy and the potential reason for these differences on line 127-133.

Reviewers' comments:

Reviewer #3 (Remarks to the Author):

My comments are submitted as attached document. I have few images that I have included in the text which was not possible to include here. Please have a look at the attached document with my report-

In the second revision" of the manuscript, tilted, "Gene clustering and copy number variation in alkaloid metabolic pathways of opium poppy", authors have done a decent job while answering concerns for my comments as well as second reviewer. I do still have few issues and feel that it requires attention. I am also responding in behalf of second reviewes comments. Below are texts with the image that I wanted to include.

Reviewer #2 comments-

1. I understand authors comment on comparison between quality of two genomes and lack of genetic maps. In Figure S4, authors showed that several genes depicted as red where newly assigned to this genome, while blue ones were placed on different chromosomes. I understand red ones, and that's an achievement that authors claim and showed by comparing genome. I am concern about the ones that were placed on different chromosomes. How to explain that? Is it because the two species or cultivar used for genome sequencing are different and hence rearrangement? Is this misassemble in previous assembly that were corrected in this study, or is this misassembly in the previous one. As authors have attempted to improve this genome, this clarification is essential and does put doubts in my mind as which one I should trust. Again, I am not contesting the fact that many new genes were assigned to scaffolds, but then how to know if these assignments are correct. Evidence is therefore required here. For example, if authors could randomly pick few of the genes that were misplaced on different chromosomes, and show through PCR that indeed their position in their genome is the correct one, that would give more confidence on this assembly. I guess that's the cheapest and fastest way to address this. Authors could also use BioNano sequencing to provide orthogonal evidence of their assembly, which will be even better.

2. Figure S2 and quality of HiC. Well, I understand challenges in terms of paralogs and actual biology that could provide no so clean HiC map. But I still believe that Reviewer #2 comment is valid and this HiC maps does justify additional QC as suggested by Reviewer #2 and #3. The main reason is this-

How authors would explain encircled regions of this HiC map. I accept that diagonal is relatively neat and does qualify as a decent alignment of contigs. But what these regions means? Segmental duplications? Paralogs? But these looks like the entire region seems to have undergone rearrangements, so what authors think about it? The shown region here are relatively neat and seems to have diagonal too, almost entire length of a big genome segment.

Rest, I am satisfied with other comments from the authors.

Reviewer #2 comments-

1. I must admit that this is really surprising to know the mistake that authors have now accepted in terms of explaining contig N50 value. I completely understand that authors did a honest mistake by mentioning contig N50 as 4Mb instead of 0.4Mb. But then, authors in the first revision, did resequencing and reassembly. Authors must have admitted this mistake in their first revision, and that would have been acceptable to me. But authors only revealed after asking same thing for second time. As a reviewer, I do trust authors that all information has been provided in good faith as I cannot see all data and analysis. But in this case, I am obviously not confident. I am not sure if the final Contig N50 said this time is 7Mb or 0.7Mb. I am sorry for such a rude statement, but hope authors

understand my point. Again, most of the results that were mentioned in the first submitted report is unchanged. So, I am bound to have questions in terms of such mistakes remains in this study.

2. About proposed FISH analysis, the reason to propose this was for many genes that were assigned to different chromosomes in Guo et al., genome is now assigned to another place. So, experimental evidence seems a rational expectation to solve this discrepcy. Probably, this is due to different cultivar genomes (Highly unlikely, but not impossible), but atleast author need to acknowledge this and then discuss somewhere. This genome is supposed to be an improvement, and then community will need to choose one of these two for all future studies. So, this is least one could expect from authors.

3. Authors have mentioned about an algorithm that were used to identify global and local gene clusters. But is that algorithm is be made available for users? That is not part of supplementary information, isn't? I think that it is important to provide that info.

Rest of my concerns were addressed by the authors, and I will thank them for their effort.

Dear Reviewer,

Thank you for your careful consideration of our manuscript and for the constructive feedback. In this revision, we have fixed one problem with a figure, changed some text, and added a supplementary figure to clarify an issue that you raised, as described below.

I have outlined the responses to specific points below, including our new attempts at a PCR-based validation and a review of the PacBio validation we reported in the previous revision (but which was not mentioned in your last review), which clearly shows that our assembly has made a substantial improvement over the Guo et al. assembly. As we describe below, any PCR-based assessment of a draft genome assembly will be much less conclusive than the PacBio validation we already conducted, and further validation using FISH or BioNano would require months of testing and optimization, putting it beyond the scope of the current paper. We strongly feel that the validation of our refined genome assembly provides sufficient grounding for the main aims of our paper, which are to assess patterns of gene clustering in the benzylisoquinoline alkaloid pathway, and covariation between expression, metabolite production, and genome architecture.

We acknowledge that future draft assemblies will further correct inaccuracies and refine our understanding of this important plant genome, and we have included extensive supplementary information to facilitate comparison with future assemblies, including our updated assembly and files indicating the location of all annotated genes, as well as the scripts and data necessary to generate all of the figures, tables, and datapoints in our manuscript.

We think that this updated manuscript now addresses all of the issues that you had previously raised, and we thank you again for helping us to greatly improve this paper.

Sincerely,

Sam Yeaman

In the second revision" of the manuscript, tilted, "Gene clustering and copy number variation in alkaloid metabolic pathways of opium poppy", authors have done a decent job while answering concerns for my comments as well as second reviewer. I do still have few issues and feel that it requires attention. I am also responding in behalf of second reviewers comments-

Reviewer #2 comments-

1. I understand authors comment on comparison between quality of two genomes and lack of genetic maps. In Figure S4, authors showed that several genes depicted as red where newly assigned to this genome, while blue ones were placed on different chromosomes. I understand red ones, and that's an achievement that authors claim and showed by comparing genome. I am concern about the ones that were placed on different chromosomes. How to explain that? Is it because the two species or cultivar used for genome sequencing are different and hence rearrangement? Is this misassemble in previous assembly that were corrected in this study, or is this misassembly in the previous one. As authors have attempted to improve this genome, this clarification is essential and does put doubts in my mind as which one I should trust. Again, I am not contesting the fact that many new genes were assigned to scaffolds, but then how to know if these assignments are correct. Evidence is therefore required here. For example, if authors could randomly pick few of the genes that were misplaced on different chromosomes, and show through PCR that indeed their position in their genome is the correct one, that would give more confidence on this assembly. I guess that's the cheapest and fastest way to address this. Authors could also use BioNano sequencing to provide orthogonal evidence of their assembly, which will be even better.

> We appreciate your concerns here, but we are curious why you didn't mention the previous validation efforts that we reported in our most recent submission? As reported there, we conducted validation using PacBio sequence data from reads used by Guo et al. to assemble their genome (from the HN1 cultivar) as well reads we had sequenced from the PS7 cultivar (the same cultivar we used for Hi-C), which we reported in Table S3. Importantly, the PS7 PacBio reads were not used anywhere in the assembly process leading to either the Guo et al. or our Hi-C assembly, and therefore provide independent

data (these reads were used in the Masurca assembly included in our very first manuscript, which was abandoned in our first revision).

This validation exercise showed that PacBio data supported the joins made in our genome **4-5 times more often** than it supported joins made in the Guo et al. genome. Specifically, we found that the HN1 and PS7 datasets mapped to the Guo et al. genome in only 5.1% and 4.3% of the breakpoint joins, whereas the HN1 and PS7 datasets mapped to our Hi-C genome in 25.9% and 24.1% of the breakpoint joins. Based on this assessment alone, it should be absolutely clear that our genome provides a superior assembly, and that this is true based on the sequence information from either of the cultivars. We have changed some of the wording on page 7 to clarify this.

In addition to this previous validation, we have also now performed several attempts at validation using PCR amplifications near breakpoints in the genome assemblies, which are outlined in detail in the Appendix at the end of this response to reviews. Based on the very low success rate for these PCR-based attempts to validate either the Guo et al. assembly or our Hi-C assembly, it is clear that this is not a particularly useful approach to validation, likely because of the highly repetitive nature of the genome. We therefore conclude that the PacBio validation described above provides much clearer results. Because these results show that our assembly has made an improvement over the previous version, we strongly feel that this has provided sufficient support for the rest of the paper and that no further validation activities are necessary. We readily acknowledge that there will be errors in both of these assemblies, and these errors will inevitably be corrected by future drafts of the genome. To facilitate comparison of our results with future assemblies, we have submitted a Dryad archive that includes complete annotations and tables, as well as analysis scripts needed to generate all figures, tables, and datapoints (Dryad archive can be accessed here: https://datadryad.org/stash/share/XDIjSHUe8V7bupu1451dB7R3Hs2xj6V9kQZ4uAVlI-s).

2. Figure S2 and quality of HiC. Well, I understand challenges in terms of paralogs and actual biology that could provide no so clean HiC map. But I still believe that Reviewer #2 comment is

valid and this HiC maps does justify additional QC as suggested by Reviewer #2 and #3. The main reason is this- How authors would explain encircled regions of this HiC map. I accept that diagonal is relatively neat and does qualify as a decent alignment of contigs. But what these regions means? Segmental duplications? Paralogs? But these looks like the entire region seems to have undergone rearrangements, so what authors think about it? The shown region here are relatively neat and seems to have diagonal too, almost entire length of a big genome segment.

You bring up an interesting point here, and we have modified the manuscript accordingly. These regions off the main diagonal are likely the result of the whole genome duplication that was the main subject of the Guo et al. (2018) paper. When a whole chromosome has experienced a duplication some time in the past, there will be a residual signal of similarity along the diagonals with other chromosomes that share homologous regions, caused by uncertainty in the mapping of the Hi-C reads in areas where there is high identity. Consistent with this explanation, we have matched up the chromosomal regions that Guo et al. (2018) identified as being homologous with the ancestral eudicot chromosome that had duplicated, and found that all of the cases with substantial off-diagonals in our Hi-C map correspond to the same chromosomes implicated in tandem duplications, as shown below (the coloured boxes around the chromosomes in Fig. S11 from the Guo et al. paper match the highlighted regions in the Hi-C contact map). Thus, the Hi-C is working appropriately. To clarify this issue, we have included a new Figure (S5) showing the left-hand panel of the image below, as well as adding some text to the main manuscript on page 6.

Rest, I am satisfied with other comments from the authors.

Reviewer #2 comments-

1. I must admit that this is really surprising to know the mistake that authors have now accepted in terms of explaining contig N50 value. I completely understand that authors did a honest mistake by mentioning contig N50 as 4Mb instead of 0.4Mb. But then, authors in the first revision, did resequencing and reassembly. Authors must have admitted this mistake in their first revision, and that would have been acceptable to me. But authors only revealed after asking same thing for second time. As a reviewer, I do trust authors that all information has been provided in good faith as I cannot see all data and analysis. But in this case, I am obviously not confident. I am not sure if the final Contig N50 said this time is 7Mb or 0.7Mb. I am sorry for such a rude statement, but hope authors understand my point. Again, most of the results that were mentioned in the first submitted report is unchanged. So, I am bound to have questions in terms of such mistakes remains in this study.

> We understand that this may cause some uncertainty, but we would like to re-affirm that there has been no dishonesty nor intent to misrepresent anything on our part. We did not bring this up because our initial draft assembly from our first submission where this mistake occurred is no longer being used in our current paper, so going into a lengthy discussion of the problems with our first assembly did not seem warranted. I apologize for any uncertainty that this may have caused, and in hindsight, we should have included a lengthier discussion of the reasons for changing our approach in the first revision. We are including all raw data and analysis scripts in this resubmission if you wish to check them.

2. About proposed FISH analysis, the reason to propose this was for many genes that were assigned to different chromosomes in Guo et al., genome is now assigned to another place. So, experimental evidence seems a rational expectation to solve this discrepcy. Probably, this is due to different cultivar genomes (Highly unlikely, but not impossible), but atleast author need to acknowledge this and then discuss somewhere. This genome is supposed to be an improvement, and then community will need to choose one of these two for all future studies. So, this is least one could expect from authors.

We have provided substantial evidence that our genome is an improvement over the previous assembly using the PacBio data, as discussed above (Table S3). Conducting further PCR or BioNano validations at this point would take months of refinement and testing, which is beyond the scope of this paper.

3. Authors have mentioned about an algorithm that were used to identify global and local gene clusters. But is that algorithm is be made available for users? That is not part of supplementary information, isn't? I think that it is important to provide that info.

We did not previously include the raw data and scripts in a public archive because we wanted to avoid being "scooped" before publication. As mentioned above, all raw data and scripts are now included in a Dryad archive. The "global" and "incremental" algorithms are included in "table_s6_s7_test_enrichment_clusters_pathway_genes_permute_all_sizes_random_trial.R" and "table_s6_s7_test_spatial_clustering_incremental.R", respectively.

Rest of my concerns were addressed by the authors, and I will thank them for their effort.

Thank you very much for your time. Our manuscript has greatly improved after incorporating your helpful comments.

Summary of changes made in this revision:

- Added a new Figure S5 (as described above) and text describing off-diagonal signal in Hi-C data.
- Clarified text relating to the accessions used in our PacBio validations.
- Fixed a mistake in the plotting of Figure 6a, which had been mis-plotted when the axis was rescaled in the last submission for better clarity and the mistake was not noticed. The correct version was originally plotted as Figure 2a in our first re-submission, so this change simply restores the correct plotting that was originally done.
- A few minor wording changes and updating of supplementary figure numbers.

APPENDIX: PCR validation

We attempted PCR-based validation around two types of breakpoints that were joined by our Hi-C assembly and/or by the Guo et al. assembly: (1) amplification spanning the gaps on both ends of the contigs that were placed in different locations in the Guo + Hi-C genomes (i.e. those shown in Figure S4); (2) amplification spanning gaps that also had PacBio reads mapping across them in both genomes.

Ideally, validation 1 would provide some indication of which assembly is more accurate, while validation 2 would show a high level of support for both assemblies, as such gaps were bridged in the same way by two different kinds of technology (linkage mapping and Hi-C) and were also subsequently validated by PacBio mapping. As it was not possible for us to obtain the DNA for the HN1 cultivar, we used DNA extracted from cultivar PS7 (the one used for our Hi-C) to evaluate both assemblies.

The primer sequences used and their corresponding locations in the genome are included as a supplementary table for review only (Primsers_Validation1, Primers_Validation2).

[above]: Table showing regions of the two assemblies where mismatches occurred in contig placement (joint number corresponds to the lane naming in the gel image).

Validation 1: This gel image shows amplifications for 7 primer pairs from our Hi-C assembly ("PGA assembly"), as well as primers designed for 3 known gene regions included as positive controls ("PC#"), on the top lanes, and 5 primer pairs from the Guo et al. assembly ("NC assembly"), on the bottom lanes. Some of the regions flanking the joins are full of repetitive sequences, in which case we couldn't find any specific sequences suitable for primers. The expected sizes of each of the amplifications based on each genome assembly are listed on the right hand side of the image.

Mismatch Regions	Hi-C_position	Adjacent_joints	Guo_position	Adjacent_joints
1	PGA_scaffold9	PGA_scaffold9_joint23:47834991-47935091	NC_039364.1	NC_039364.1_joint32:134432896-134538116
	(cScaf_10)	PGA_scaffold9_joint24:52098294-52198394		NC_039364.1_joint33:138701319-138803662
2	PGA_scaffold9	PGA_scaffold9_joint25:52797111-52897211	NC_039364.1	NC_039364.1_joint36:139427525-139531356
	(cScaf_10)	PGA_scaffold9_joint26:70925017-71025117		NC_039364.1_joint37:157559162-157660354
3	PGA_scaffold8	PGA_scaffold8_joint1:452076-552176	NC_039358.1	NC_039358.1_joint106:244111398-244212398
	(cScaf _9)	PGA_scaffold8_joint2:4790508-4890608		terminal
4	PGA_scaffold10	terminal	NC_039368.1	NC_039368.1_joint64:125407408-125508740
	(cScaf _11)	PGA_scaffold10_joint1:1515491-1615591		NC_039368.1_joint65:126974231-127076367
5	PGA_scaffold10	PGA_scaffold10_joint2:13083338-13183438	NC_039368.1	NC_039368.1_joint66:138544114-138647538
	(cScaf _11)	PGA_scaffold10_joint3:14679651-14779751		terminal

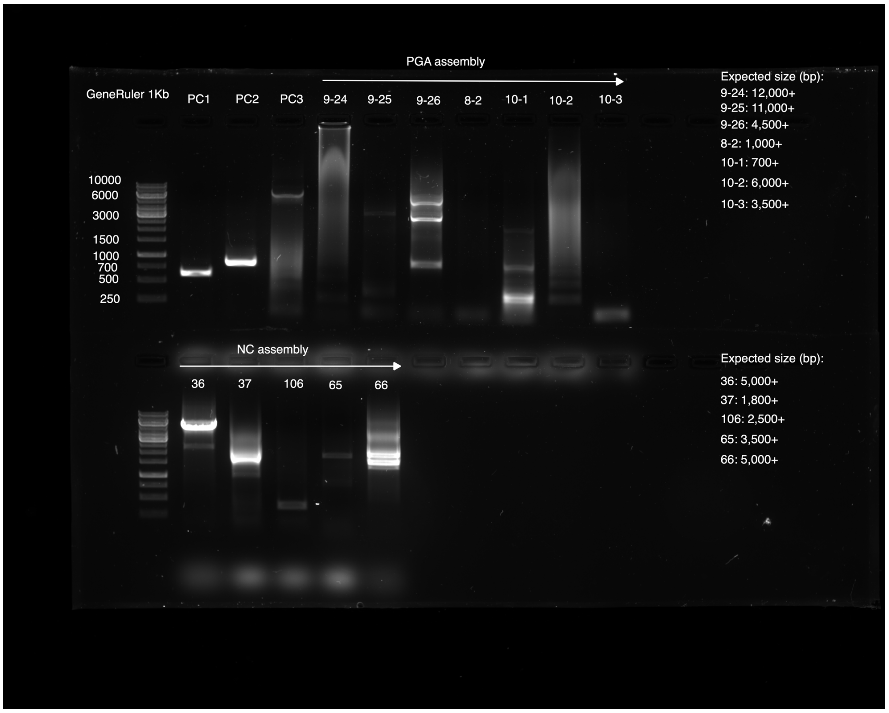

As is clear from this image, positive controls bands were amplified in PC1, PC2 and PC3. Although the PC3 band was relatively dim, it was detected at the expected position at ~7000+ bp. By contrast, none of the amplifications spanning the gaps in either of the assemblies provides clear support for any of joins. Potential amplifications occur for NC36 & NC37 (Guo genome joins), and for PGA9-26 & PGA10-1 (Hi-C joins), but further optimization and substantial conformational analysis would be required. Given the highly repetitive nature of these difficult-to-assemble regions of the genome and the time constraints for resubmission, we opted to instead attempt a different approach to validation (#2, below).

Validation 2: The gel images below show amplifications for primer pairs designed on either side of assembly joins where there was agreement between the Guo et al. assembly and the Hi-C assembly, and also have reads mapping from the PacBio validation data. For each amplification, the expected sizes are listed at the bottom of the image. In theory, these amplifications should therefore work very well if this approach is suitable for providing good validations, as 3 other unrelated and independent technologies support these joins. Instead, we see only 2 of the 16 test amplifications showing clear bands at the expected size (0_61 and 4_59, which both happen to locate

in relatively low repetitive regions), as well as a few suggestive bands occurring of incorrect size (note that the positive control in the first gel image [PC] shows a clear but faint band at the expected position, which would likely be clearer with further optimization of the reactions).

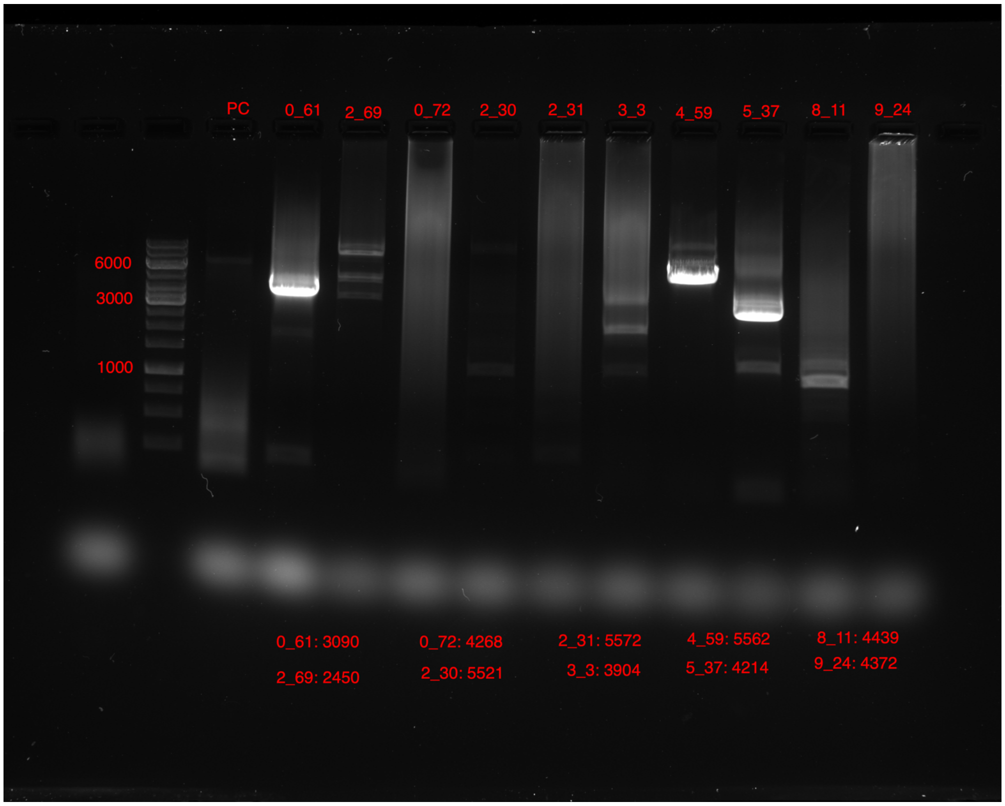

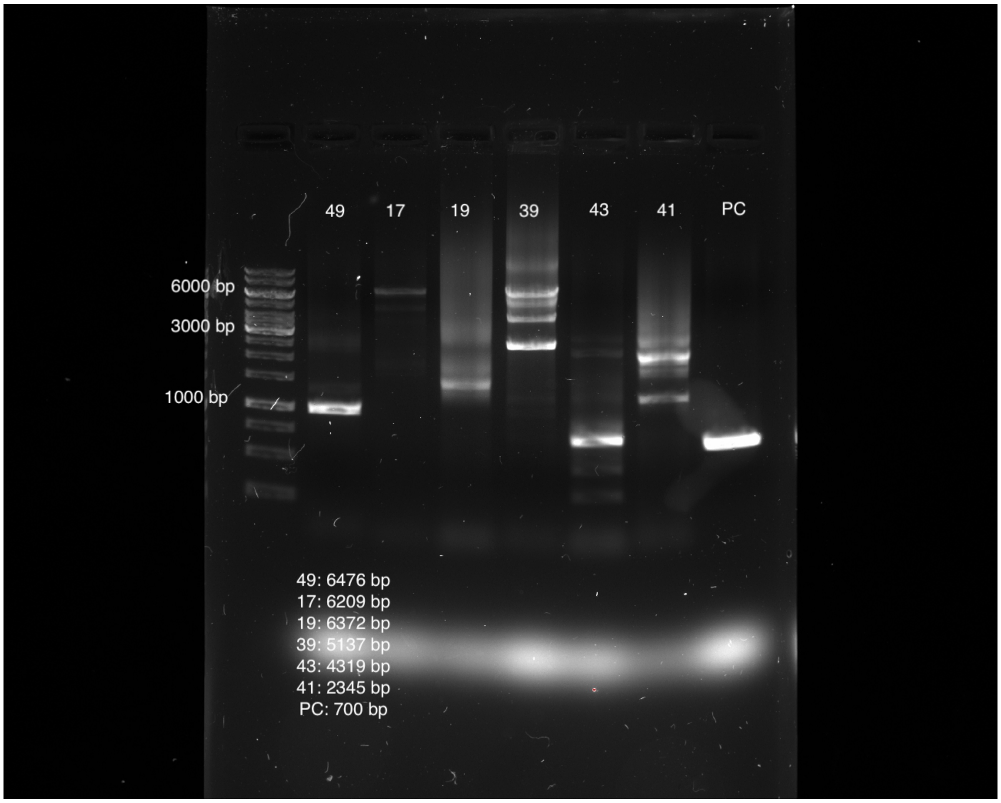

Taken together, these images show that PCR-based validation approaches do not provide conclusive evidence. It is possible that the approach could be developed into a reliable methodology with substantial work, but we feel that this effort is beyond the scope of our paper. We must again emphasize that our PacBio validation provided the means to assess whole-assembly error rates across all joins, rather than picking and choosing a few breakpoints for validation, and that this validation clearly showed that our Hi-C assembly had improved the accuracy of the joins overall.

For reference, we have included below IGV images showing the assembly regions covered in Validation (2), which shows the highly repetitive nature of these regions.

<table>
<tr><th>Gap Regions</th><th>IGV</th><th>Primer Premier 5</th></tr>
<tr><td>PGA_scaffold0_joint49:169838236-169938336</td><td>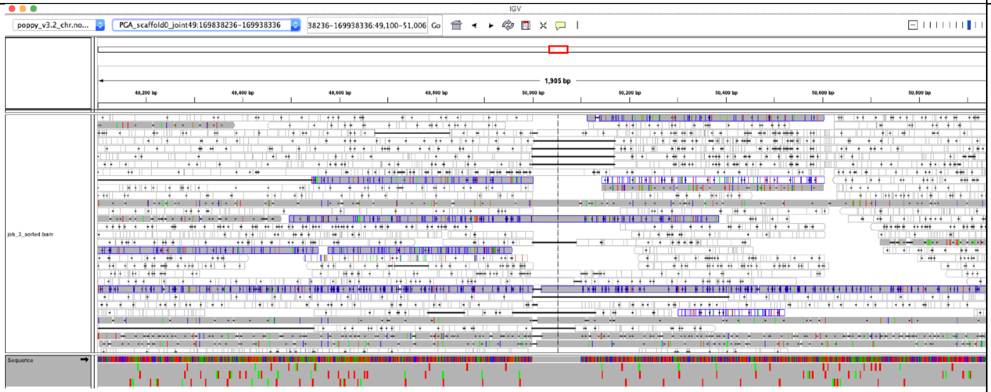</td><td>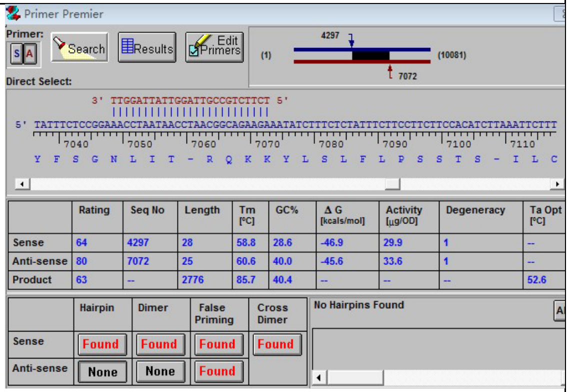
</td></tr>
<tr><td>PGA_scaffold0_joint61:235976187-236076287</td><td>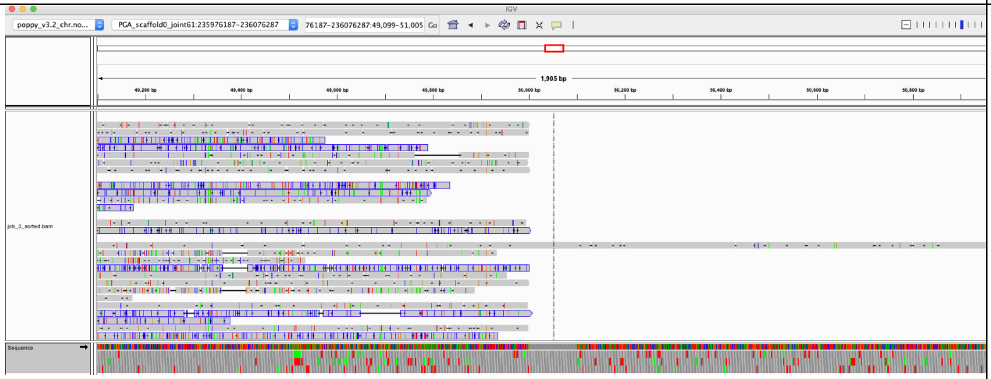</td><td>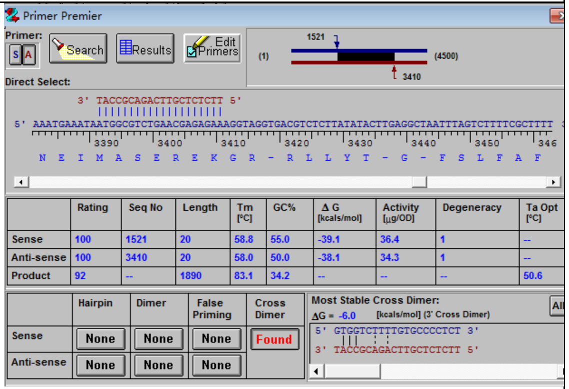
</td></tr>
</table>

PGA_scaffold0_joint72:289675116-289775216

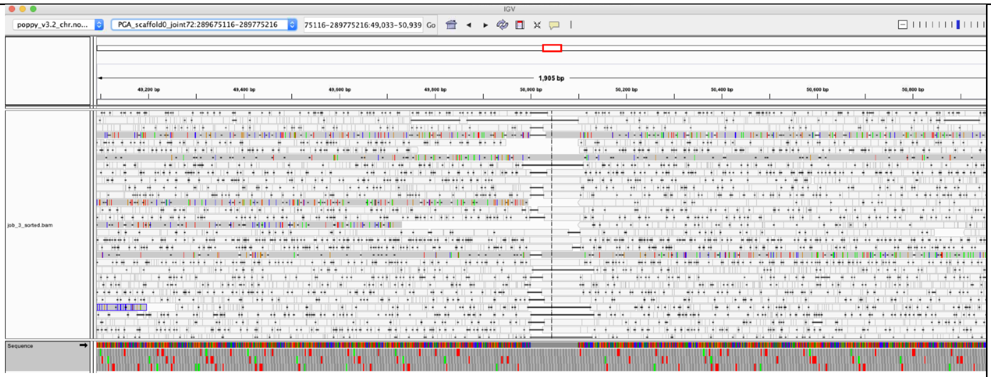

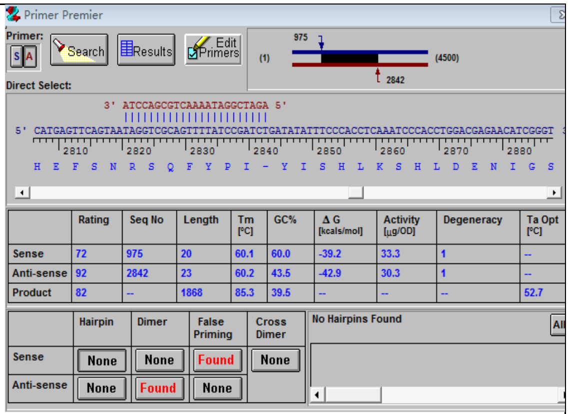

PGA_scaffold2_joint17:25194620-25294720

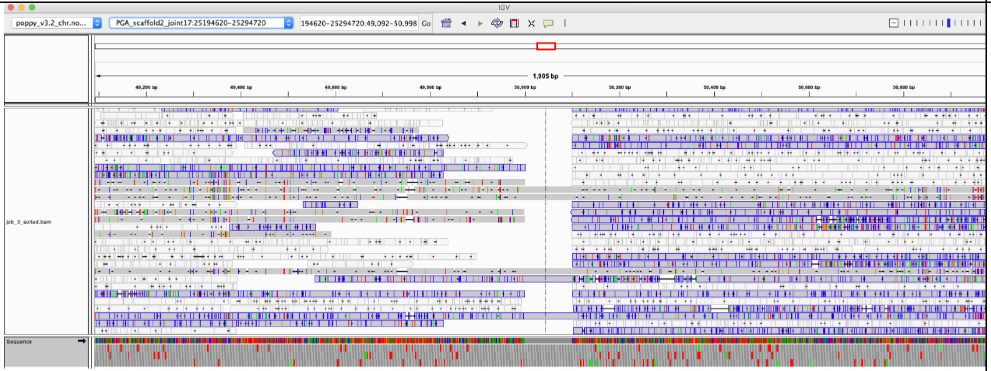

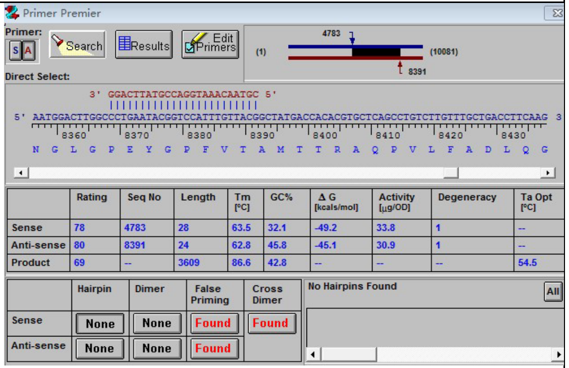

PGA_scaffold2_joint19:36829277-36929377

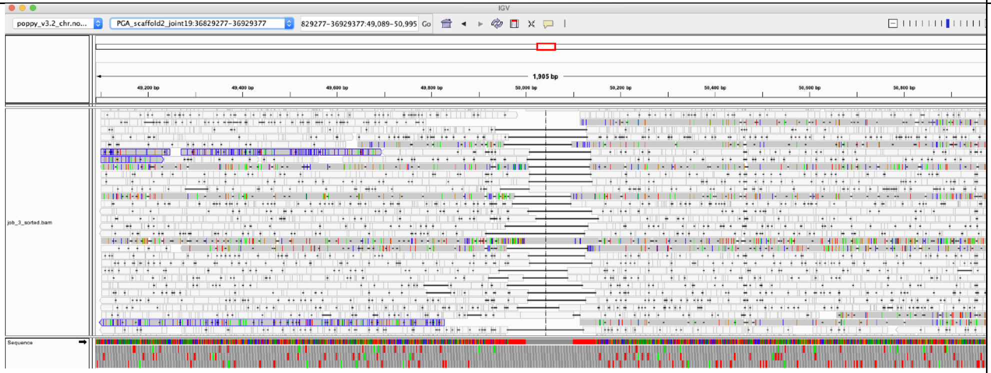

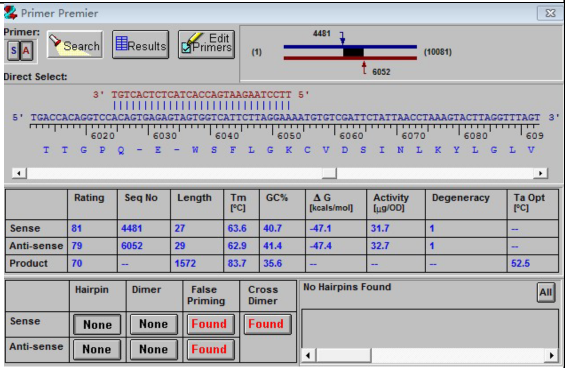

PGA_scaffold2_joint30:75904353-76004453	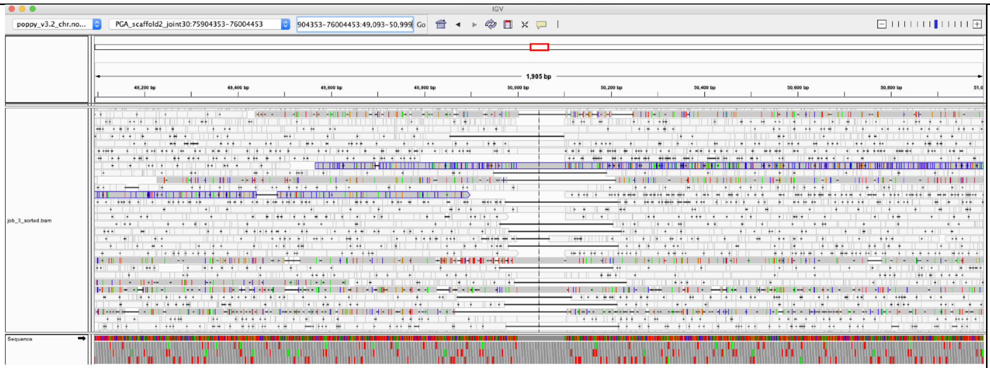	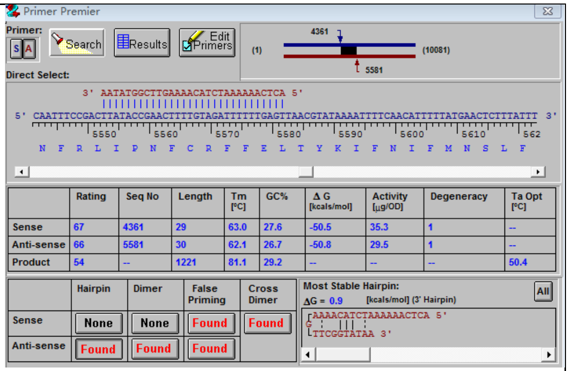
PGA_scaffold2_joint31:77476746-77576846	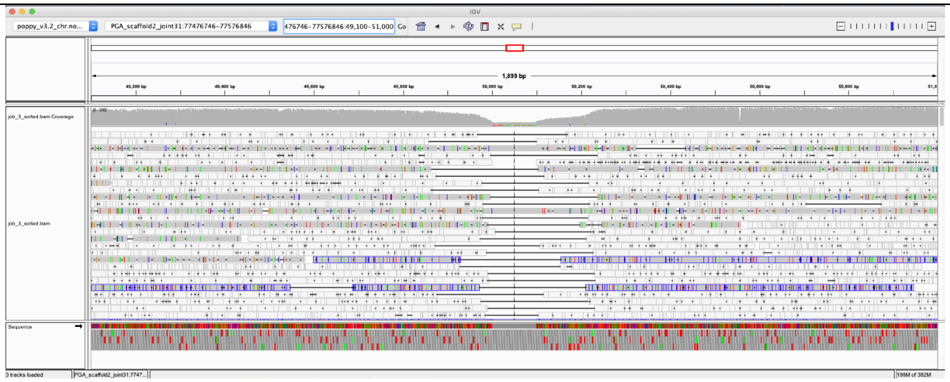	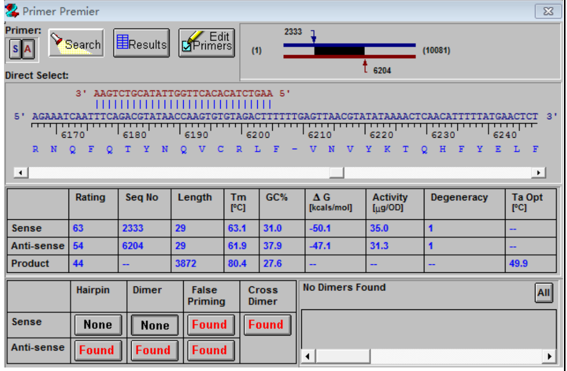
PGA_scaffold2_joint39:104165655-104265755	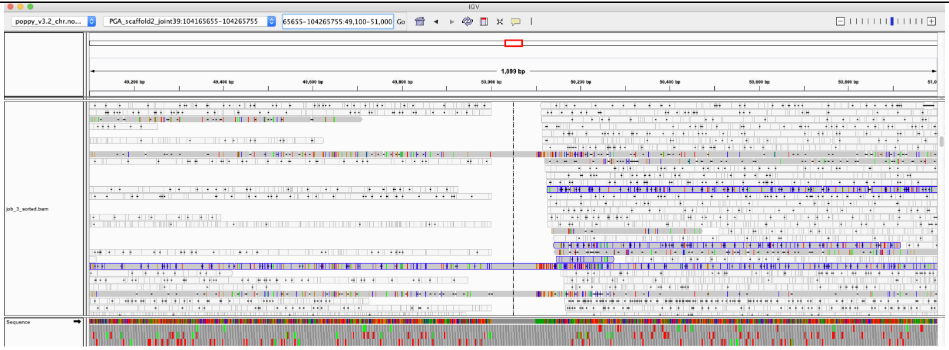	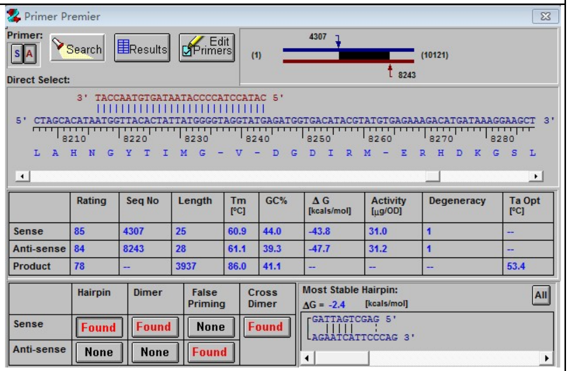

PGA_scaffold2_joint69:243029818-243129918

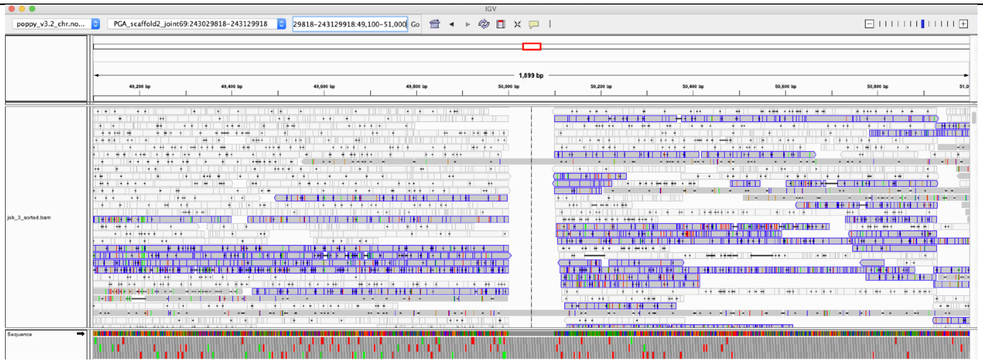

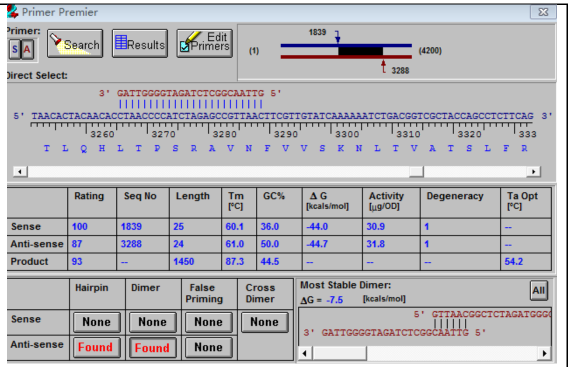

PGA_scaffold3_joint3:6328104-6428204

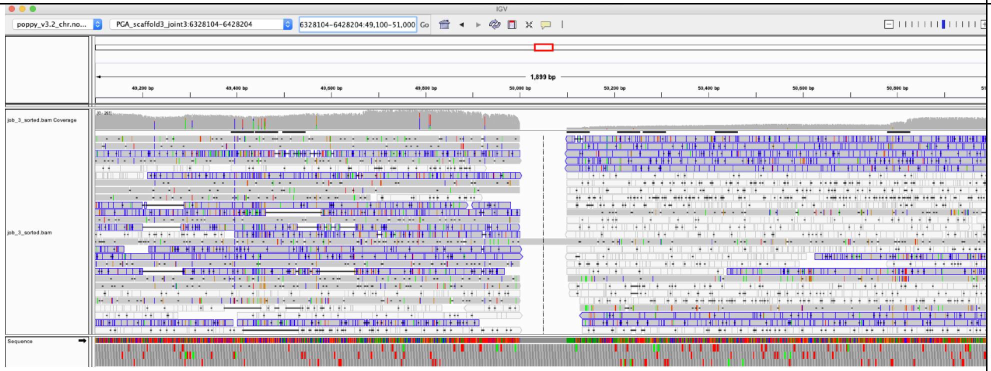

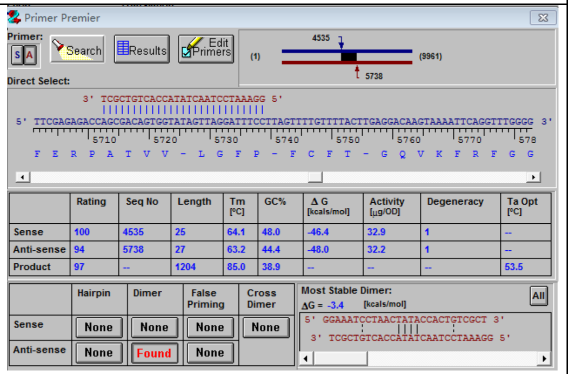

PGA_scaffold3_joint43:131897145-131997245

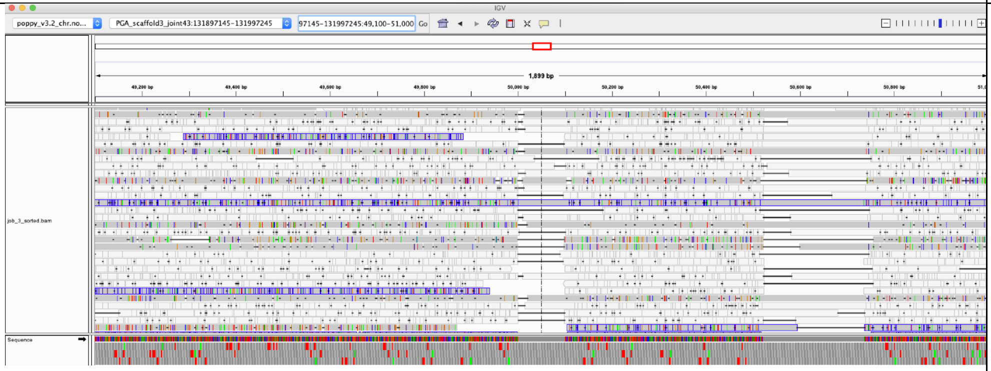

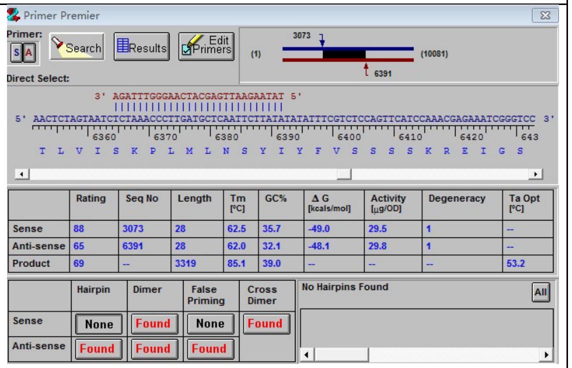

PGA_scaffold4_joint59:225790664-225890764	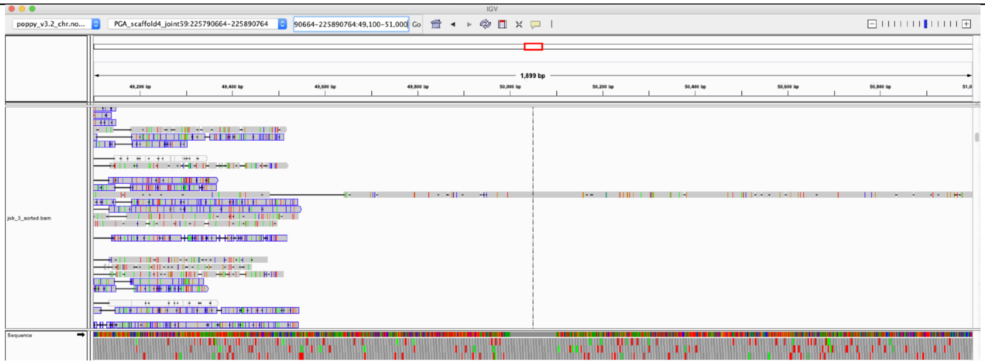	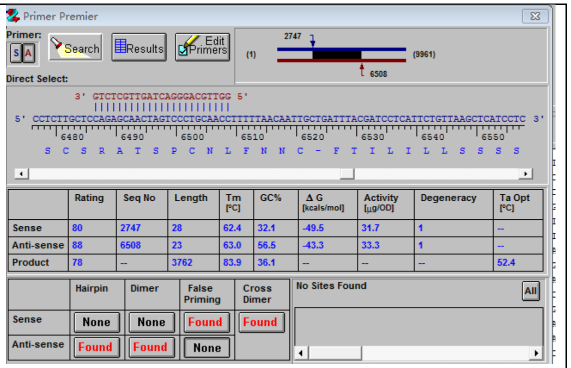
PGA_scaffold5_joint37:101221260-101321360	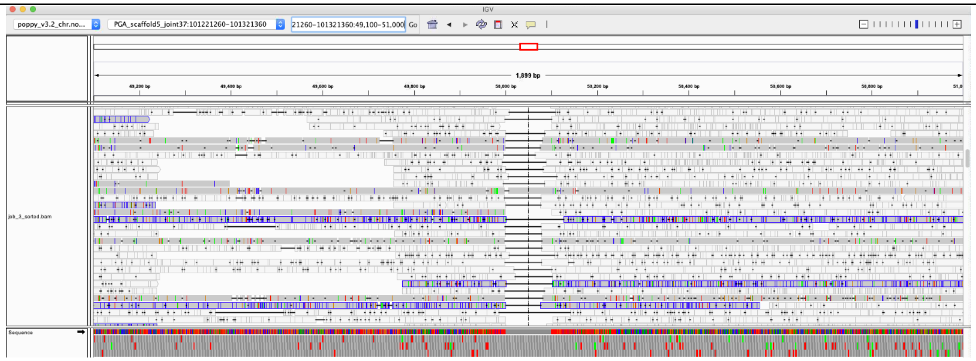	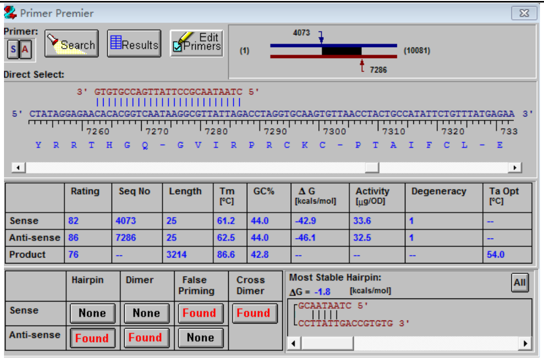
PGA_scaffold5_joint41:124544034-124644134	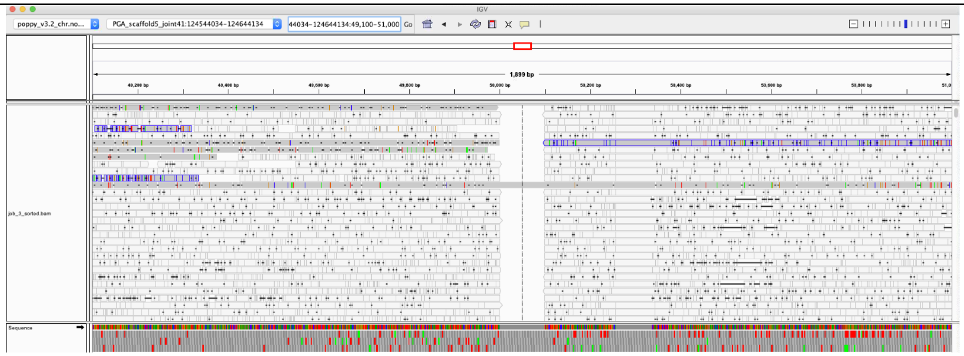	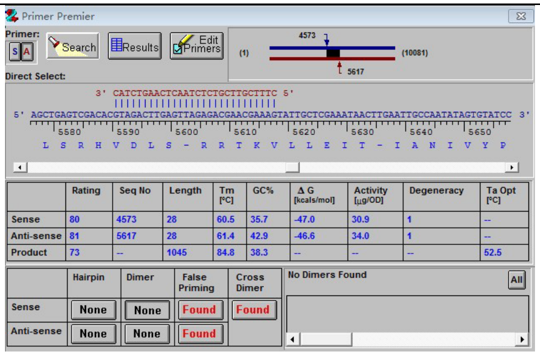

PGA_scaffold8_joint11:49344937-49445037

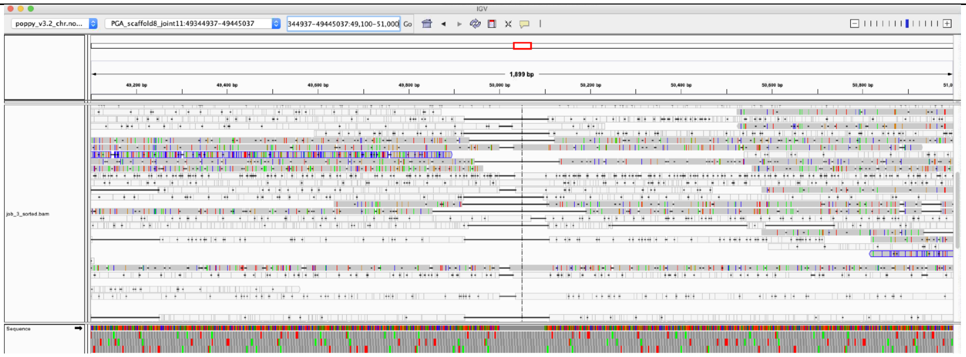

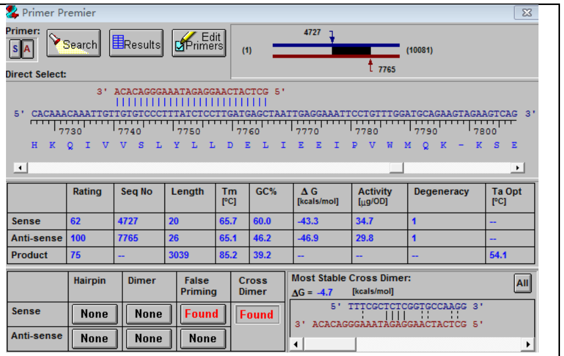

PGA_scaffold9_joint24:52098294-52198394

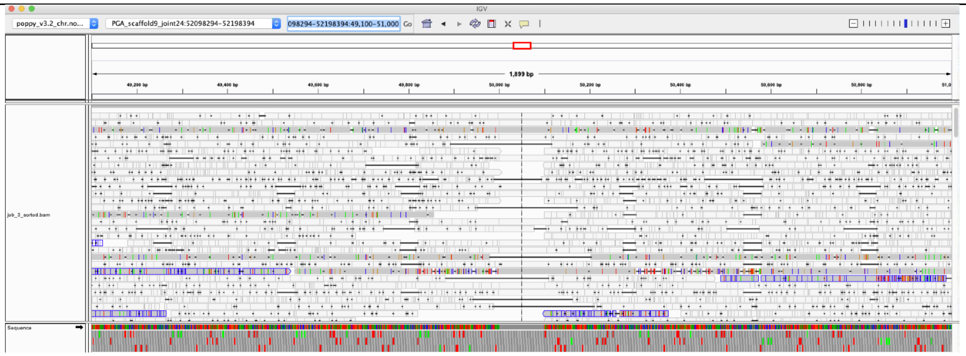

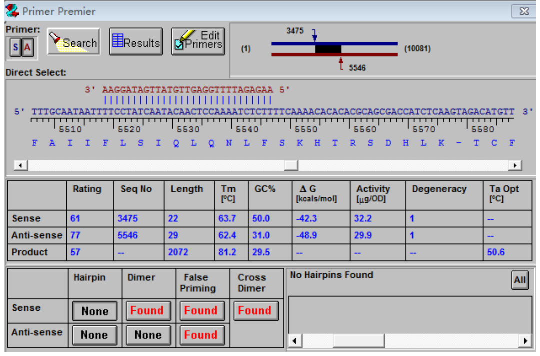

REVIEWERS' COMMENTS:

Reviewer #3 (Remarks to the Author):

Thank you so much for your response and attemptes to clarify all my concerns.

First of all, the reason for asking a comparison between the submitted genome assembly and one that was published by Guo et al was because I am curious if there are any structural variants that authors could report. I admit missing on taking a note on the alignment that authors reported justifying their claim on the quality of the genome assembly. My apologies. I agree with the authors comment that indeed the current genome assembly is an improvement over Guo et al work, with many contigs being assigned to scaffolds now. The modification in the text bring this point quite clearly and I am satisfied with the authors response.

REVIEWERS' COMMENTS:

Reviewer #3 (Remarks to the Author):

Thank you so much for your response and attemptes to clarify all my concerns.

First of all, the reason for asking a comparison between the submitted genome assembly and one that was published by Guo et al was because I am curious if there are any structural variants that authors could report. I admit missing on taking a note on the alignment that authors reported justifying their claim on the quality of the genome assembly. My apologies. I agree with the authors comment that indeed the current genome assembly is an improvement over Guo et al work, with many contigs being assigned to scaffolds now. The modification in the text bring this point quite clearly and I am satisfied with the authors response.

Thank you for your hard work helping us improve this paper.
